# *In vivo* and *in vitro* investigations provide insights into maleidride biosynthesis in fungi†

**DOI:** 10.1039/d5ra02147b

**Published:** 2025-07-04

**Authors:** Katherine Williams, Catherine E. Spencer, Kate M. J. de Mattos-Shipley, Anjali D. Shah, Trong-Tuan Dao, Jonathan A. Davies, David M. Heard, Zhongshu Song, Ashley J. Winter, Matthew P. Crump, Christine L. Willis, Andrew M. Bailey

**Affiliations:** a School of Biological Sciences, Life Sciences Building, University of Bristol 24 Tyndall Avenue Bristol BS8 1TQ UK andy.bailey@bristol.ac.uk; b School of Applied Sciences, University of West of England Coldharbour Lane Bristol BS16 1QY UK katherine12.williams@uwe.ac.uk; c School of Chemistry, University of Bristol Cantock's Close Bristol BS8 1TS UK

## Abstract

Maleidrides are a family of polyketide-derived natural products isolated from filamentous fungi, that can exhibit significant bioactivities. These compounds are classified according to the size of their central carbocyclic ring, to which one or more maleic anhydride moieties are attached. The studies described herein provide important insights into maleidride biosynthesis, in particular the pathways to the nonadrides scytalidin and castaneiolide, and the octadride zopfiellin. We propose a supportive role for isochorismatase-like enzymes, which are commonly encoded within maleidride biosynthetic gene clusters, in facilitating α-ketoglutarate dependent dioxygenase-mediated catalysis. This is evidenced by gene deletions as well as enzyme assays, for two maleidride biosynthetic pathways: that of zopfiellin, from *Diffractella curvata*; and of scytalidin, from *Scytalidium album*. These experiments collectively underscore the significance of the isochorismatase-like enzymes in the catalytic process of α-ketoglutarate dependent dioxygenases. Feeding studies with either scytalidin or an unsaturated analogue to *D. curvata* Δ*zopPKS* both gave the 5,6-diol, castaneiolide and the structure was confirmed by NMR and X-ray crystallography. Furthermore, a putative biosynthetic gene cluster for castaneiolide biosynthesis was identified from a *de novo* genome assembly of the native producer, *Macrophoma castaneicola*.

## Abstract 2

Isochorismatase-like enzymes play a supportive role in α-ketoglutarate dependent dioxygenase mediated catalysis to increase maleidride diversity.

## Graphical abstract

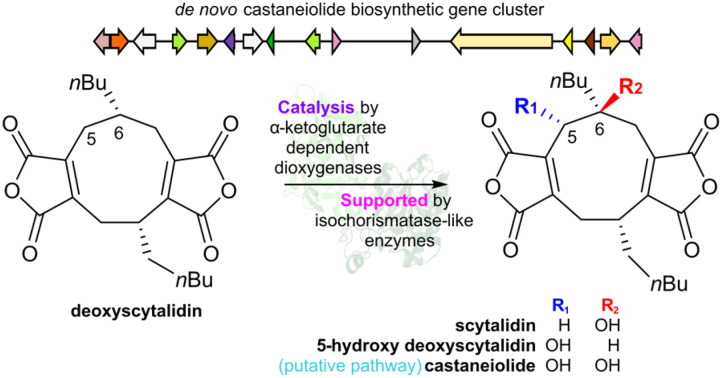

## Introduction

**Fig. 1:**
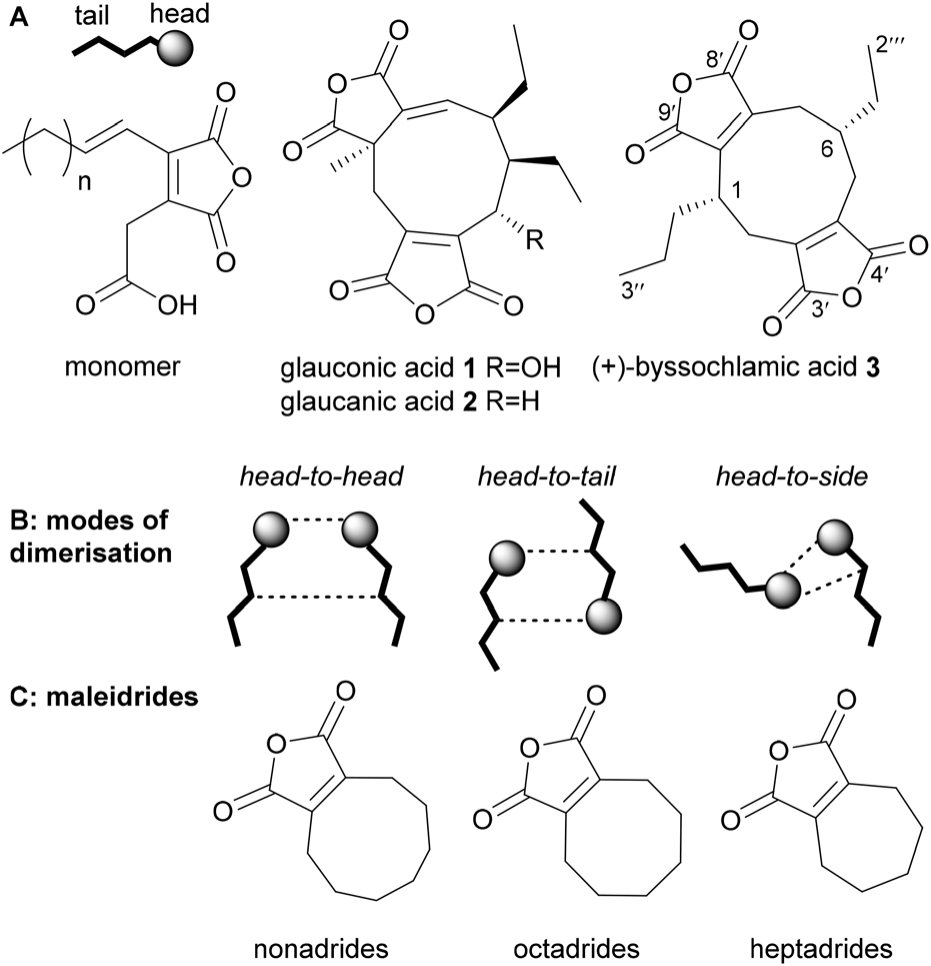
(A) Structures of the first characterised nonadrides, and the monomer required for the dimerisation step. (B) Pictorial representation of the various modes of dimerisation. (C) Core structure of the maleidrides. Figure amended from ref. 11.

The biogenesis of the maleidride family of fungal natural products has intrigued scientists since the early 1960s, when the first structural elucidation of several nonadrides (a class of maleidrides) was achieved.^1^ These were glauconic and glaucanic acids 1 and 2, and (+)-byssochlamic acid 3.¶¶The numbering systems used for the maleidrides varies greatly in the literature and shows no consistency. We have previously proposed a common, and more systematic system based on the size of the ring (1–9, 1–8, 1–7 as appropriate) beginning at the carbon alpha to the maleic anhydride ring, which gives the lowest numbers to the side chains. The maleic anhydride carbons would be numbered with a prime, appropriate to the ring numbering, hence 3′, 4′ and 8′, 9′ for byssochlamic acid 3, with 1′′, 2′′, *etc.* for the first side chain, and numbering from the ring junction and 1′′′, 2′′′, *etc.* for the second side chain.^13^^1^ The unusual structures of these compounds led Sutherland and co-workers^1,2^ to propose that the biosynthesis of the nonadrides may involve a dimerisation of two monomers in different ‘modes’ (Fig. 1B).

Subsequent labelling studies have confirmed this hypothesis.^3,4^ Recently, significant progress has been made towards understanding the genetic and enzymatic basis behind the biosynthesis of the nonadrides.^5–10^

Maleidrides are classified according to the size of the carbocyclic ring to which one or more maleic anhydride moieties are attached. They include the nonadrides consisting of a nine-carbon central carbocyclic ring, octadrides (with an eight-carbon core), and heptadrides (seven carbons) (Fig. 1C). Many maleidride natural products possess potent bioactivities, making them attractive pharmaceutical or agrochemical lead compounds.^11^

**Fig. 2:**
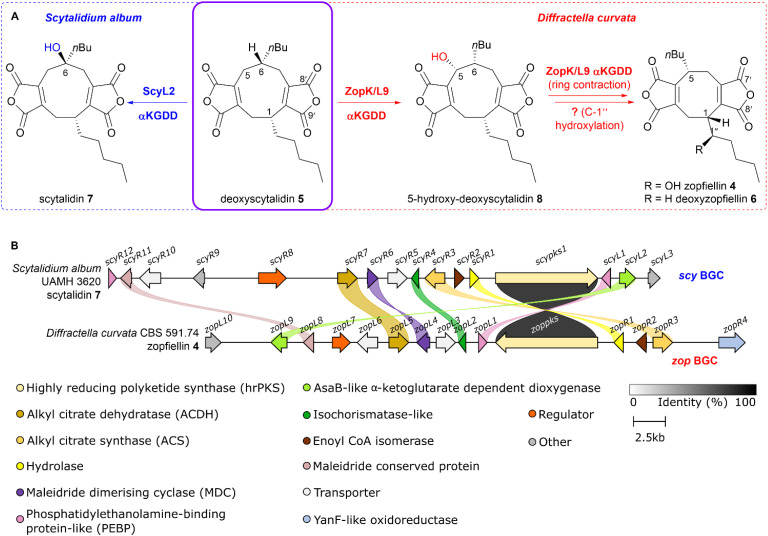
(A) Divergent biosynthetic pathways in *S. album* and *D. curvata*, from the common intermediate, deoxyscytalidin 5.^13^ (B) clinker^14^ comparison between the zopfiellin 4 and scytalidin 7 BGCs. Links between homologous genes are shown using their specific colour, except for the PKSs where the links are shown according to the percentage identity (see identity scale bar). Coloured links do not denote specific percentage identities, only that sequence homology is above 25%. BGCs are aligned on the PKS and links between transport and regulatory genes have been removed for clarity.

Until recently, the biogenesis of the octadrides, and how this integrates within the broader maleidride biosynthetic pathway, has remained obscure. However, studies reported in 2020 have begun to provide insight into the biosynthetic pathway of the octadride, zopfiellin 4 (Fig. 2A).^12,13^

Interestingly, it has been shown in the biosynthesis of zopfiellin 4, that the nonadride and octadride pathways are linked by a multi-step oxidative ring contraction from the nonadride precursor, deoxyscytalidin 5, to the octadride deoxyzopfiellin 6.^13^ Deoxyscytalidin 5 is a common intermediate in both the zopfiellin 4 pathway in *Diffractella curvata* and the biosynthetic pathway to the nonadride scytalidin 7 in *Scytalidium album* (Fig. 2A).^13^

During our previous investigations into the divergent pathways to zopfiellin 4 and scytalidin 7, the genomes of both *D. curvata* and *S. album* were sequenced and the maleidride biosynthetic gene clusters (BGCs, *zop* BGC and *scy* BGC, Fig. 2B) were identified, showing each contained a highly reducing polyketide synthase (hrPKS). The PKS is the core synthase within the maleidride pathway, catalysing formation of the polyketide chain, which is subsequently condensed with oxaloacetate to produce the maleidride monomer. The *zopPKS* and *scyPKS* were deleted from each BGC confirming the identity of these clusters.^13^

The gene encoding an α-ketoglutarate dependent dioxygenase (αKGDD–*scyL2*), situated within the *scy* BGC was also deleted, confirming that it is responsible for 6-hydroxylation of deoxyscytalidin 5 to produce scytalidin 7 (Fig. 2A).^13^

Our *in vitro* studies on the αKGDD ZopL9 from the zopfiellin 4 pathway^13^ also revealed that ZopL9 was capable of 5-hydroxylation of deoxyscytalidin 5 to produce 5-hydroxy-deoxyscytalidin 8 and ring contraction to deoxyzopfiellin 6 (Fig. 2A). 5-Hydroxy deoxyscytalidin 8 was confirmed as an intermediate in the zopfiellin 4 pathway, where enzyme assays with ZopL9 and 8 showed turnover to deoxyzopfiellin 6.^13^ However, we observed an apparent low catalytic efficiency with this enzyme for this conversion.^13^ These results are consistent with the *in vitro* experiments reported by Oikawa and coworkers^12^ on the orthologous ZopK. The low efficiency of the ring contraction step during the *in vitro* conversion of nonadride 8 to an octadride seems incongruent with the apparently high metabolic flux through the zopfiellin 4 pathway in *D. curvata* itself and suggests that an additional factor may be required for this step to proceed efficiently.||*Diffractella curvata* Δ*zopR4* is a genetically impure deletion strain which still contains some WT nuclei.

Within the zopfiellin 4 and scytalidin 7 BGCs, several genes remain where encoded function is difficult to predict given current knowledge of maleidride biosynthesis.^15^ For example, within both clusters are genes encoding: isochorismatase-like enzymes (ICM-like), *zopL2* and *scyR4*; enoyl CoA isomerase-like enzymes, *zopR2* and *scyR2*; and maleidride conserved proteins, *zopL8* and *scyR11*. Solely within the zopfiellin 4 BGC is a gene encoding a YanF-like oxidoreductase, *zopR4* (Fig. 2B). Herein we describe investigations of these enzymes leading to a deeper understanding of maleidride biosynthesis, and includes the identification of the factor responsible for supporting the activity of the maleidride αKGDDs.

## Results and discussion

### Investigation of isochorismatase-like enzymes

**Fig. 3:**
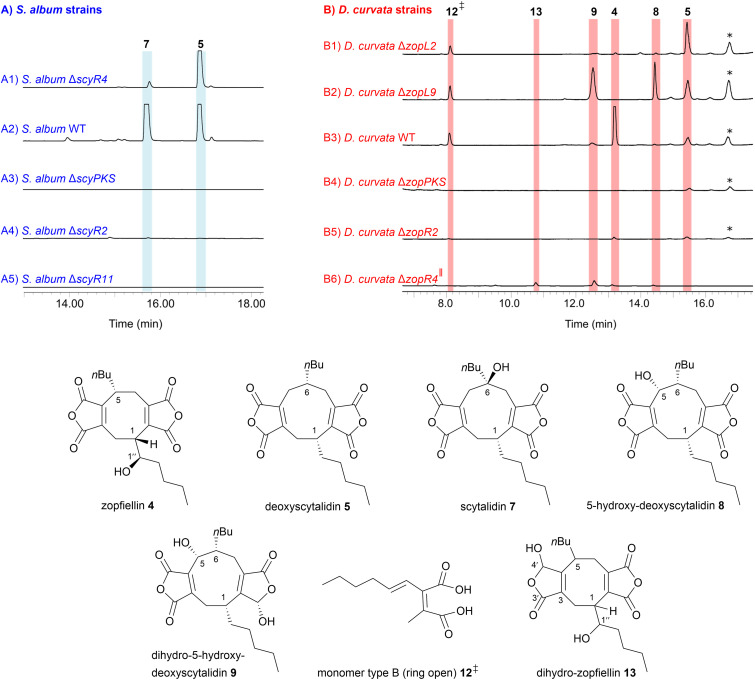
(A) ELSD chromatograms of extracts from gene disruptions in *S. album* (5–95% MeCN : H_2_O gradient). (A1) Deletion of the ICM-like hydrolase gene, *scyR4* reduces production of the mature maleidride scytalidin 7 and leads to the accumulation of the pathway intermediate, deoxyscytalidin 5. (A2) *S. album* WT. (A3) Deletion of *scy*PKS. (A4) Deletion of the enoyl CoA isomerase, *scyR2* significantly reduces scytalidin 7 biosynthesis. (A5) Deletion of the gene encoding the maleidride conserved protein, *scyR11* appears to completely abolish scytalidin 7 biosynthesis. (B) ELSD chromatograms of extracts from gene disruptions in *D. curvata* (40–95% MeCN : H_2_O gradient). (B1) Deletion of the ICM-like hydrolase gene, *zopL2* reduces production of the mature maleidride zopfiellin 4 and leads to the accumulation of the pathway intermediate, deoxyscytalidin 5. (B2) Deletion of the αKGDD encoding gene, *zopL9* leads to a loss of zopfiellin 4 production with the accumulation of 5-hydroxy-deoxyscytalidin 8 and the reduced compound 9. (B3) *D. curvata* WT. (B4) Deletion of *zopPKS*. (B5) Deletion of the enoyl CoA isomerase, *zopR2* significantly reduces zopfiellin 4 biosynthesis. (B6) Deletion of the oxidoreductase encoding gene, *zopR4*|| (incomplete genetic purity) reduces production of the mature maleidrides, however low titres of dihydro-zopfiellin 13 and dihydro-5-hydroxy-deoxyscytalidin 9 are evident. *unrelated metabolite. ‡ putative compound predicted from MS and UV data, see ESI, Fig. S10† for details.

Using methods described previously,^13^ deletions of the genes encoding ICM-like enzymes from both the scytalidin 7 and zopfiellin 4 BGCs were undertaken giving strains *S. album* Δ*scyR4* and *D. curvata* Δ*zopL2*. Analysis of the metabolic profiles of these strains by LC-MS revealed that cultures of *S. album* Δ*scyR4* gave mainly deoxyscytalidin 5 along with traces of scytalidin 7 (Fig. 3A1) whilst *D. curvata* Δ*zopL2* also accumulated deoxyscytalidin 5 and traces of 5-hydroxy-deoxyscytalidin 8 and zopfiellin 4 (Fig. 3B1). However, titres appeared significantly reduced compared to WT strains (Fig. 3A2 and B3) and both knock-out strains accumulated deoxyscytalidin 5 as the major compound. Based on these results, we hypothesised that the subsequent steps in the biosynthetic pathways of both compounds, namely 6-hydroxylation by ScyL2 to produce scytalidin 7, and the multi-step oxidative ring-contraction by ZopL9 to produce deoxyzopfiellin 6 (Fig. 2A), are supported in some way by the action of these homologous ICM-like enzymes.

Experiments by Oikawa and co-workers^12^ provide further evidence for this hypothesis: heterologous production of deoxyzopfiellin 6 did not include the *zopL2* ICM-like orthologue, *zopQ*. Expression of the monomer-forming and dimerisation genes alongside the αKGDD in the host *Aspergillus oryzae* produced deoxyscytalidin 5 as the major compound. Only small amounts of 5-hydroxy-deoxyscytalidin 8 and deoxyzopfiellin 6 were produced, again suggesting gaps in our current understanding of the pathway.^12^

**Fig. 4:**
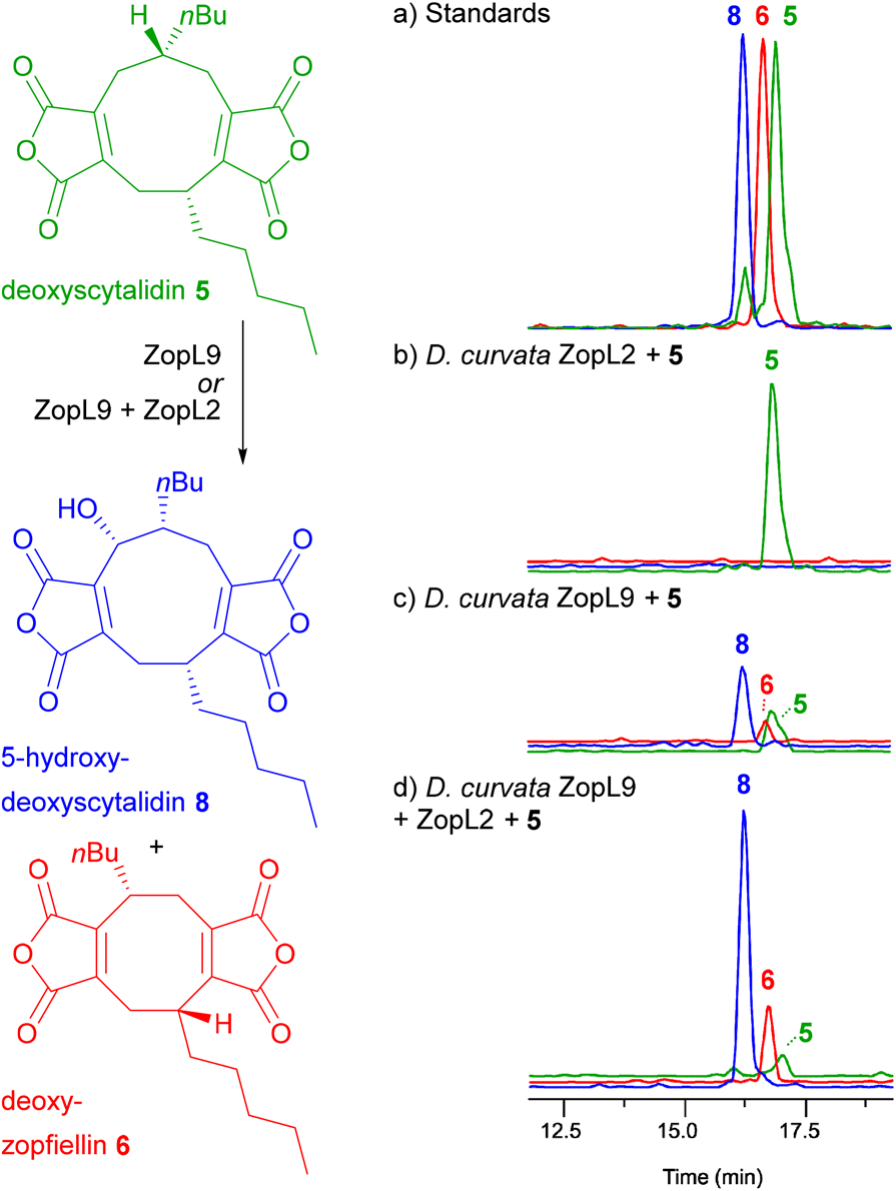
EIC ES^−^ (5–95% MeCN : H_2_O gradient) analysis of extracts from enzyme assays with ZopL9, ZopL2 and deoxyscytalidin 5. Trace (a) standards of deoxyscytalidin 5, 5-hydroxy-deoxyscytalidin 8 and deoxyzopfiellin 6. Trace (b) ZopL2 (ICM-like) plus deoxyscytalidin 5. No turnover observed. Trace (c) ZopL9 (αKGDD) plus deoxyscytalidin 5. Turnover to both 5-hydroxy-deoxyscytalidin 8 and deoxyzopfiellin 6 observed. Trace (d) ZopL9 (αKGDD) plus ZopL2 (ICM-like) plus deoxyscytalidin 5. Turnover to both 5-hydroxy-deoxyscytalidin 8 and deoxyzopfiellin 6, with increased titres, with little deoxyscytalidin 5 substrate remaining.

To determine the precise impact of ZopL2 upon the αKGDD reaction, *in vitro* assays using ZopL9 were conducted as previously,^13^ but with the addition of equal amounts of purified recombinant ZopL2 (Fig. 4). Compared to the assay with ZopL9 alone, where significant amounts of the substrate deoxyscytalidin 5 remain, and production of the octadride deoxyzopfiellin 6 is low (Fig. 4, trace c), assays with ZopL9 and ZopL2 show that most of the substrate 5 is consumed, yielding higher titres of deoxyzopfiellin 6 (Fig. 4, trace d). These results clearly demonstrate the importance of ICM-like enzymes in supporting maleidride αKGDD function. To further explore this interaction, analytical size-exclusion chromatography (SEC) of ZopL2, ZopL9, and an equimolar mixture of the two was conducted. Results (see ESI, Fig. S15†) show no evidence for association between ZopL9 and ZopL2, leaving the mechanism by which ZopL2 supports catalytic activity unresolved.

### Further investigation of αKGDD ZopL9

**Fig. 5:**
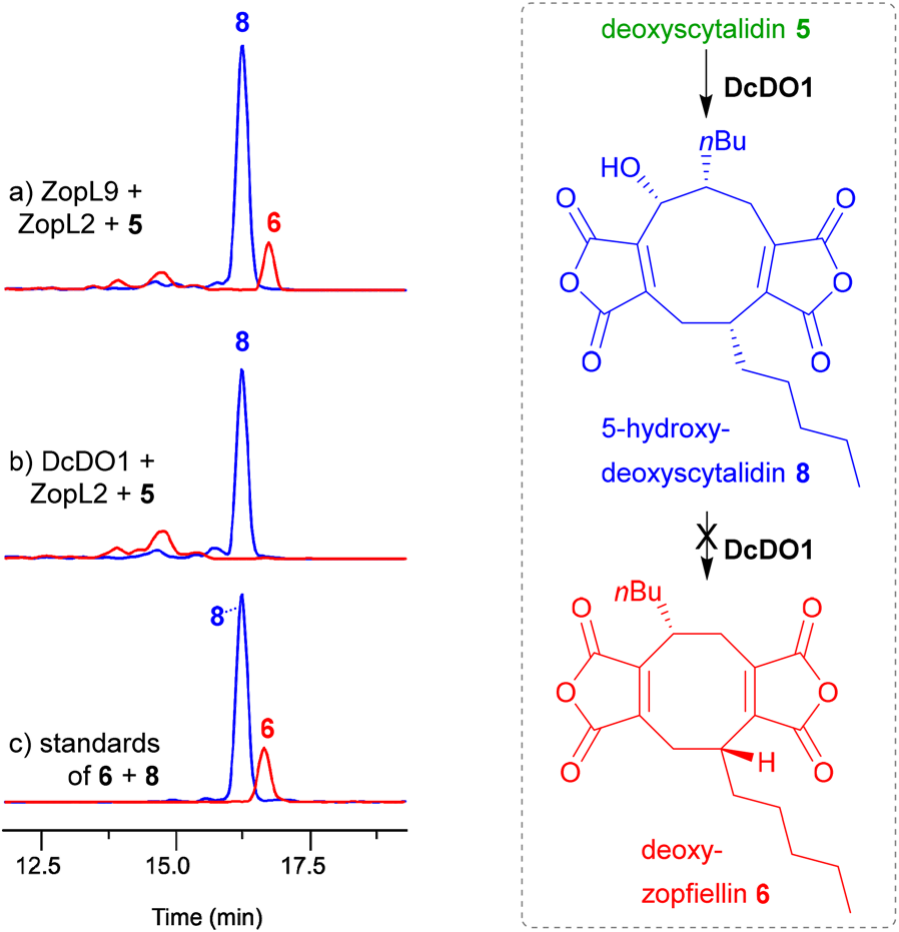
EIC ES^−^ (5–95% MeCN : H_2_O gradient) analysis of extracts from enzyme assays comparing the activity of ZopL9 to DcDO1. Trace (a) Production of both 5-hydroxy-deoxyscytalidin 8 and deoxyzopfiellin 6 catalysed by *D. curvata* ZopL9 plus ZopL2 with substrate deoxyscytalidin 5. Trace (b) production of only 5-hydroxy-deoxyscytalidin 8 catalysed by the homologue *D. curvata* DcDO1 plus ZopL2 with substrate deoxyscytalidin 5. Trace (c) standards of deoxyzopfiellin 6 and 5-hydroxy-deoxyscytalidin 8.

To further investigate the αKGDD from the zopfiellin 4 pathway, *zopL9* was deleted to create strain *D. curvata* Δ*zopL9*. This led to a total loss of zopfiellin 4 production, and in contrast to the previous *in vitro* work,^13^ unexpectedly gave an accumulation of 5-hydroxy-deoxyscytalidin 8 and the reduced compound, dihydro-5-hydroxy-deoxyscytalidin 9 (Fig. 3B2). This initially appeared to be in direct conflict with the identification of 5-hydroxy-deoxyscytalidin 8 as a product of ZopL9. However, searching the genome of *D. curvata* identified a likely *zopL9* homologue, *dcdo1* (60% protein identity between DcDO1 and ZopL9 (see ESI,† Fig. S11†)), which we reasoned may complement the hydroxylase activity of ZopL9 in the knock-out strain. *In vitro* assays with purified recombinant DcDO1, and the accessory enzyme ZopL2, demonstrated that this enzyme can indeed perform hydroxylation of deoxyscytalidin 5 to 5-hydroxy-deoxyscytalidin 8 (Fig. 5, trace b). An apparent inability of DcDO1 to perform the ring contraction step catalysed by ZopL9 (Fig. 5, traces a and b) means that the biosynthetic pathway stalls at 5-hydroxy-deoxyscytalidin 8 in *D. curvata* Δ*zopL9* and the 8-membered ring is not formed (Fig. 3B2).

### Feeding studies

**Scheme 1:**
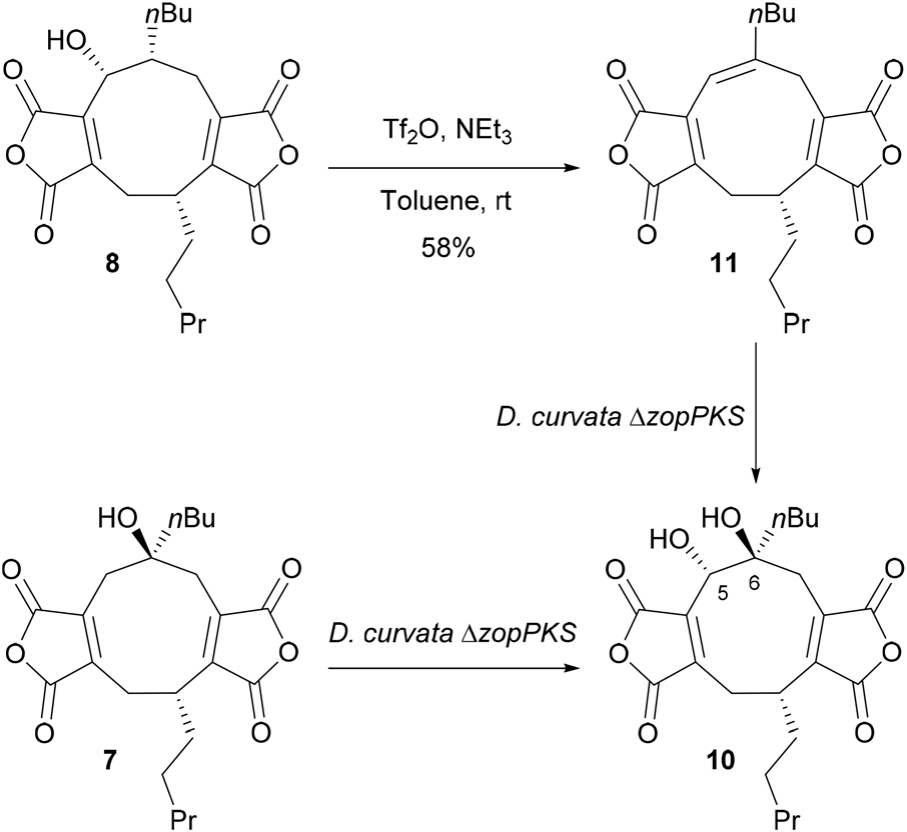
Synthesis of alkene 11 from 5-hydroxy-deoxyscytalidin 8 and feeding studies of 7 and 11 to *D. curvata* Δ*zopPKS*, to yield 5,6-diol 10.^13^

We have previously shown that feeding nonadride scytalidin 7 to *D. curvata* Δ*zopPKS* gives a 5,6-diol 10 but with no ring contraction.^13^ To gain further insights into the mechanism, we prepared 5,6-alkene 11 for feeding studies. Synthesis of alkene 11 began with isolation of 5-hydroxy-deoxyscytalidin 8, which was dehydrated using triflic anhydride giving alkene 11, in 58% yield as a single isomer (^1^H NMR, 5H, *δ* 6.27, s) (Scheme 1).

Alkene 11 was subsequently fed to the *D. curvata* Δ*zopPKS* strain and analysis of the metabolite profile by LC-MS showed that 11 was completely consumed, but neither deoxyzopfiellin 6 nor zopfiellin 4 were detected by LC-MS (see ESI, Fig. S42†). The major product had an *m*/*z* of 419 [M–H]^−^, consistent with a dihydroxylated product. The ^1^H NMR spectrum of a partially purified sample of the major metabolite was in accord with a 5,6-diol 10 having formed, with a characteristic signal for 5-H at *δ* 4.72; its ^13^C NMR spectrum showed a signal for a quaternary carbon at *δ* 78.4 assigned to C-6 while C-5 resonated at *δ* 70.0. However, assignment of the stereochemistry at C-5 and C-6 was hampered by the small amount of available material and the presence of a ring cleaved alcohol, previously isolated from a scytalidin feed to the *D. curvata* Δ*zopPKS* strain.^13^ The absence of any octadrides from the feeding study indicates that the alkene 11 is unlikely to be an intermediate in the biosynthesis of zopfiellin 4.

**Fig. 6:**
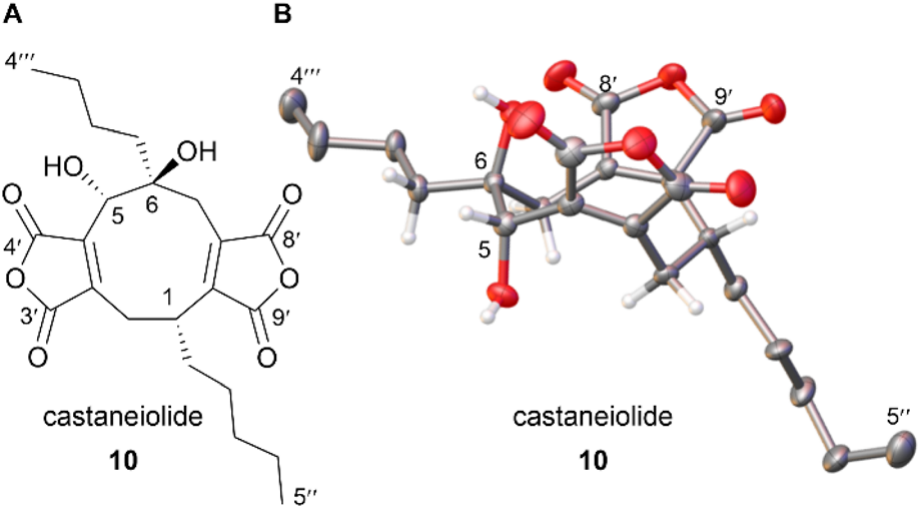
(A) Confirmed structure of the natural product castaneiolide 10. (B) Crystal structure of 10 with the anisotropic displacement parameters depicted at the 50% probability level. Disorder and hydrogens (except key hydrogens), omitted for clarity.

Castaneiolide 10 is a known 5,6-dihydroxylated nonadride, however, the biosynthetic pathway has not been explored through genetic and enzymatic studies. Castaneiolide 10 was originally isolated from *Macrophoma castaneicola*, known to cause ‘black root rot disease’ in chestnut trees (*Castanea sativa*) with the structure determined by NMR studies.^16^ A literature search indicated that the relative and absolute configuration of the natural product has not been reported. In order to fully assign the stereochemistry of castaneiolide 10 and investigate if diol 10 isolated from feeding alkene 11 and scytalidin 7 separately to *D. curvata* Δ*zopPKS* was castaneiolide 10 (Scheme 1), a sample of the natural product from the WT was required. Cultures of *M. castaneicola* strain M1-48 were grown in baffled shake flasks in MEB medium at 28 °C for 24 days and after purification by flash column chromatography, gave castaneiolide 10 (65 mg L^−1^) as a yellow solid. Extensive NMR studies, coupled with X-ray crystallography of castaneiolide 10 confirmed the absolute stereochemistry as 1*R*, 5*S*, 6*S*, where the two alkyl side chains are *syn* and the hydroxyl groups are *anti* (Fig. 6A), with castaneiolide 10 adopting a bowl-shaped structure (Fig. 6B). Comparisons of the ^1^H and ^13^C NMR spectra of castaneiolide 10 isolated from the WT fermentation and the diol 10, isolated from feeding both 7 and 11 to *D. curvata* Δ*zopPKS*, revealed the diol 10 was the same in each case (see ESI, Fig. S40 and S41†).

**Fig. 7:**
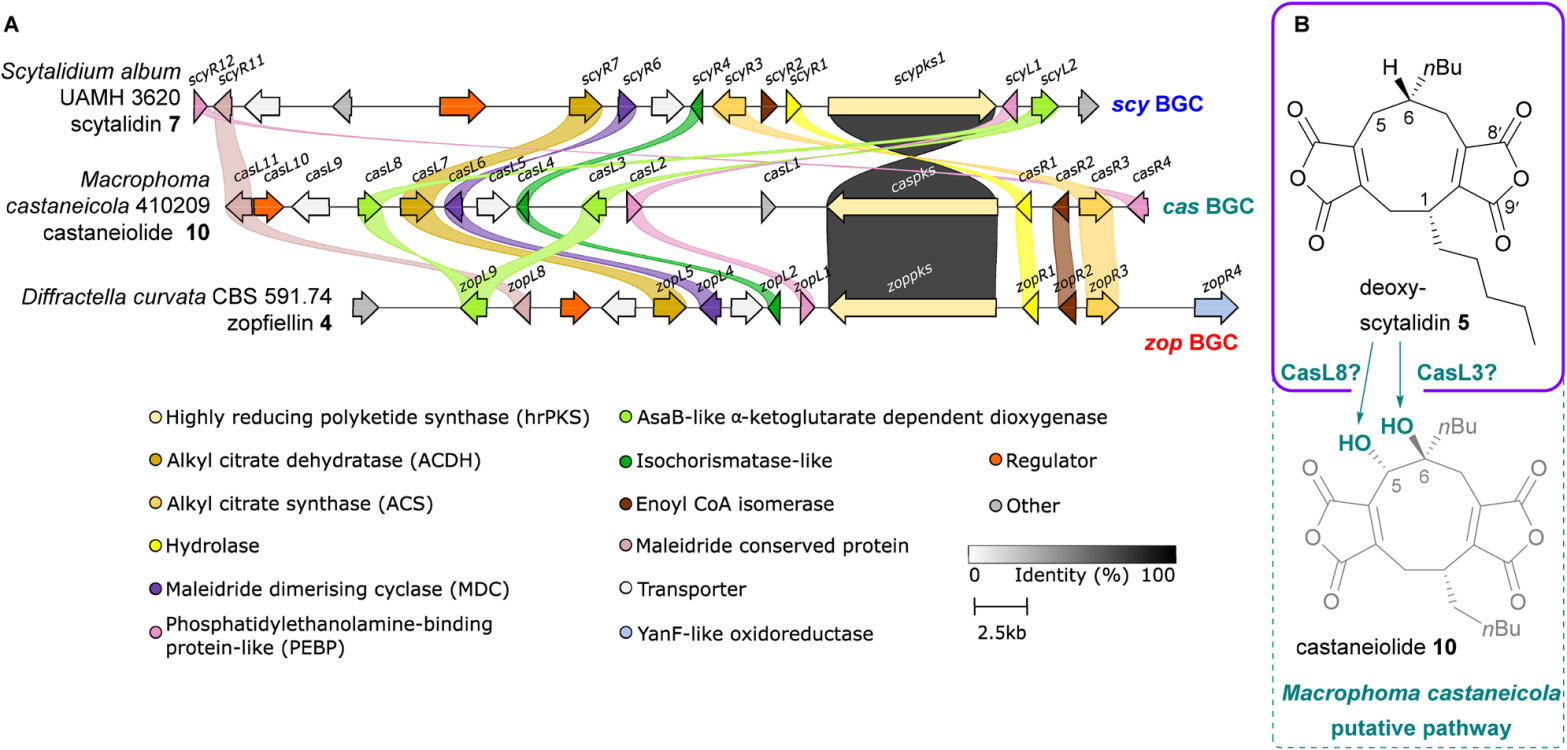
(A) Clinker^14^ comparison between the scytalidin 7 BGC, the putative castaneiolide 10 BGC, and the zopfiellin 4 BGC. Links between homologous genes are shown using their specific colour, except for the PKSs where the links are shown according to the percentage identity (see identity scale bar). Coloured links do not denote specific percentage identities, only that sequence homology is above 25%. BGCs are aligned on the PKS and links between transport and regulatory genes have been removed for clarity. (B) Based on the synteny between these three BGCs, we propose that deoxyscytalidin 5 is a likely intermediate in the castaneiolide 10 biosynthetic pathway.

*De novo* genome sequencing of *M. castaneicola* identified a putative maleidride gene cluster (*cas* BGC) through a screen for homologues to the core sequences of the byssochlamic acid 3 BGC.^6,15^ Predicted gene structures and functions for this cluster are detailed in the ESI, Table S1.† A clinker^14^ comparison of the scytalidin 7 and zopfiellin 4 BGCs with the putative castaneiolide 10 BGC highlighted significant similarities, along with high percentage identity between the PKSs (Fig. 7). These genetic similarities and structural relationships between 10, 4 and 7 suggest that deoxyscytalidin 5 is a precursor to castaneiolide 10, linking all three biosynthetic pathways (Fig. 7). The *cas* BGC contains two genes encoding αKGDDs: *casL3* and *casL8*. The high homology (see ESI, Fig. S13†) between the encoded CasL3 and ScyL2 indicates that CasL3 is responsible for the 6-hydroxylation necessary for the mature castaneiolide 10 structure, while the homology between CasL8 and ZopL9 suggests that CasL8 performs the 5-hydroxylation (Fig. 7b). Further molecular studies are needed to confirm these hypotheses.

### Further gene deletions

Three further gene disruptions were generated in *S. album* and *D. curvata*, to produce strains *S. album* Δ*scyR2*, *D. curvata* Δ*zopR2* and *S. album* Δ*scyR11*. These genes encode two enoyl CoA isomerase-like enzymes and a maleidride conserved protein respectively. Both types of genes are common to maleidride clusters, but their precise functions are hard to predict.^15^ Some evidence suggests that the enoyl CoA isomerase-like enzymes may be involved in producing an enoyl CoA substrate with the specific geometry required to satisfy the substrate requirements of the alkylcitrate synthase.^9,15^ The phenotype of the *S. album* Δ*scyR2* strain showed a significant, but not complete, reduction in the titres of both scytalidin 7 and deoxyscytalidin 5, which suggests that ScyR2 plays a pivotal, if not essential, role in scytalidin 7 biosynthesis (Fig. 3A4). The phenotype of the *D. curvata* Δ*zopR2* strain also showed an almost complete cessation of zopfiellin 4 biosynthesis (Fig. 3B5). Furthermore, a peak that is present in the *D. curvata* WT which corresponds to the ring open type B monomer 12 predicted for zopfiellin 4 biosynthesis is not apparent in the *D. curvata* Δ*zopR2* strain (Fig. 3B5).

These gene deletion experiments provide further evidence for a role for the enoyl CoA isomerases prior to monomer formation.^15^

The strain *S. album* Δ*scyR11* (deletion of the gene encoding the maleidride conserved protein) showed a complete cessation of both scytalidin 7 and deoxyscytalidin 5 production (Fig. 3A5). Homologues to *scyR11* are present in 44% (excluding highly similar BGCs) of confirmed and orphan maleidride BGCs.^15^ A gene encoding a homologue to the maleidride conserved proteins (*alnI*) is found within the asperlin BGC, which encodes another polyketide-based compound. Similarly, deletion of *alnI* fully eliminated production of asperlin.^17^ The precise role of ScyR11 is difficult to predict due to a lack of any fully characterised homologues, but the gene deletion phenotype suggests that it is crucial for the production of scytalidin 7.

Finally, the putative oxidoreductase from the zopfiellin 4 BGC, ZopR4 was investigated. In comparison to the scytalidin 7 BGC, *zopR4* is one of only two unique genes, the other being *zopL9* which encodes the αKGDD that performs the multi-step ring contraction reaction (Fig. 2B). This suggested that ZopR4 could be responsible for installing the hydroxyl group at C-1′′, which is absent in scytalidin 7.

To date, production of a genetically pure strain of *D. curvata* Δ*zopR4* proved impossible to construct. However, strain *D. curvata* Δ*zopR4*|| (still containing some WT nuclei), was analysed for its metabolic profile (Fig. 3B6). Increased amounts of a previously uncharacterised related peak were observed in the crude extract, which had a *m*/*z* of 391 [M–H]^−^, two mass units higher than zopfiellin 4, and a UV_max_ of 263 nm. The product was isolated using preparative HPLC and analysed by NMR revealing the compound was dihydro-zopfiellin 13, which has not previously been reported in the literature. A signal at *δ*_H_ 5.91 ppm which correlates in the HSQC to *δ*_C_ 97.3 ppm is characteristic of a reduced anhydride group.^18^ Additionally, it was seen that carbons 3′ and 4 were shifted downfield whilst carbon 3 had a lower chemical shift, when compared to the NMR spectrum of zopfiellin 4. The position of the reduction was determined from key HMBC correlations (see ESI, Fig. S46†). Whilst the stereochemistry of dihydro-zopfiellin 13 has not been determined, it is predicted that centres 1, 5 and 1′′ will follow the same stereochemistry as that of zopfiellin 4.

## Conclusions

**Scheme 2:**
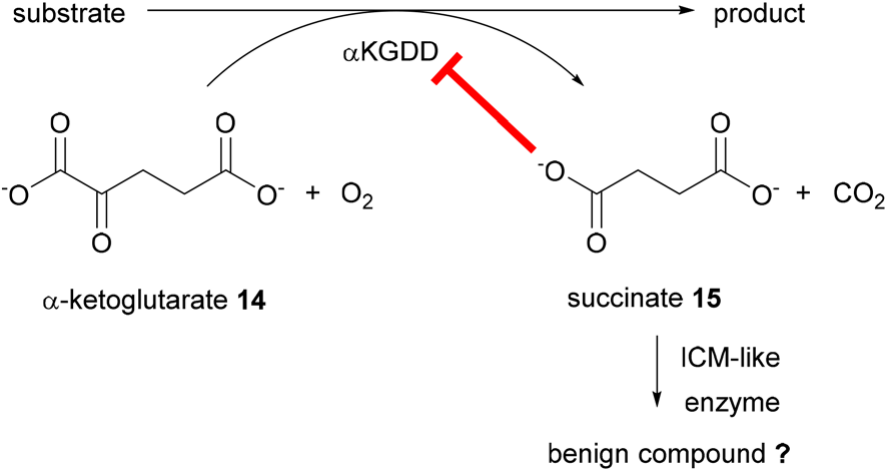
A plausible role for the maleidride ICM-like enzymes in supporting αKGDD activity by reducing the inhibitory succinate 15.

These experiments have provided new insight into the complex biosynthetic pathways to the maleidride family of fungal natural products. In particular, the isochoristmase-like enzymes have a significant role in the reactions catalysed by maleidride αKGDDs. Deletion of the ICM-like genes from either the scytalidin 7 or the zopfiellin 4 BGCs leads to accumulation of the substrate of the respective αKGDDs, deoxyscytalidin 5. Enzyme assays provide further evidence for this supportive role. Previous *in silico* analysis has predicted maleic acid as a substrate for the maleidride ICM-like enzymes,^15^ which could suggest a plausible supporting role for these enzymes. During αKGDD catalysis, α-ketoglutarate 14 is used as a co-substrate, and is converted to the by-product, succinate 15. Succinate 15 has been shown to be inhibitory to αKGDDs, thus leading to reduction in αKGDD activity.^19,20^ We propose that as succinate 15 is a known analogue of maleic acid,^21^15 could therefore be a substrate for the ICM-like enzymes, which may convert succinate 15 to a more benign compound, ultimately increasing the αKGDD activity (Scheme 2).

Of the maleidride BGCs that have been characterised, three further clusters contain αKGDD genes, the rubratoxin,^8^ cornexistin,^7^ and phomoidride^10^ BGCs.^15^

The rubratoxin gene cluster, which contains four αKGDDs, also contains an ICM-like gene, encoding RbtQ,^8^ which is highly homologous to ZopL2 and ScyR4 (over 68% identity for both, see ESI, Fig. S12†).^8^ However, no molecular studies have been conducted on RbtQ, therefore its influence on rubratoxin biosynthesis is currently unknown. Conversely, within the cornexistin and phomoidride BGCs, no homologous ICM-like genes are present.^15^ This is despite the significant role the αKGDD, TstK, plays in phomoidride biosynthesis,^10^ and the important C-2 hydroxylation catalysed by the cornexistin αKGDD, PvL5.^7^ Whilst the co-location of ICM-like and αKGDD genes is certainly not universal within other fungal natural product BGCs, there are some other examples, such as the BGC for viridicatin.^22^ The precise mechanism of their supportive role awaits further investigation.

The other genes investigated during this work have demonstrated significant impacts on maleidride biosynthesis. Deletion of either the enoyl co-A isomerase, or the gene encoding the maleidride conserved protein, showed a substantial decrease in the production of the mature maleidride, as well as pathway intermediates. Furthermore, dihydro-zopfiellin 13 was isolated from strain *D. curvata* Δ*zopR4*||. We predicted that ZopR4 would catalyse the side chain hydroxylation, however, oxidation of dihydro-zopfiellin 13 to zopfiellin 4 is not unreasonable given that the closest homologue to ZopR4 in the SwissProt database, YanF, is responsible for the oxidation of an alcohol to a ketone.^23^ However, given the impurity of the deletion strain, the role of ZopR4 is currently unsubstantiated. At present, the enzyme responsible for the conversion of deoxyzopfiellin 6 to zopfiellin 4, remains obscure, as no other candidate genes have been identified within the zopfiellin 4 BGC.

Castaneiolide 10 was isolated from the native producer, *M. castaneicola*, and fully characterised by NMR and X-ray crystallography. The stereochemistry of 10 has been fully elucidated as having a 1*R*, 5*S*, 6*S*-configuration, with the alkyl side chains as *syn* and the hydroxyl groups as *anti*. NMR comparisons confirmed that the 5,6-diols 10 isolated from feeds of either scytalidin 7 or alkene 11 to *D. curvata* Δ*zopPKS* are castaneiolide 10. Sequencing and assembly of the *M. castaneicola* genome revealed a putative maleidride biosynthetic gene cluster which demonstrates high homology with both the zopfiellin 4 and scytalidin 7 BGCs. This likely indicates a biosynthetic relationship between these three maleidrides.

In summary, this work not only contributes to a deeper understanding of maleidride biosynthesis, but also underscores the complexities in this intriguing group of fungal natural products. While much progress has been made, many features await further elucidation and exploration, offering fertile ground for future investigations.

## Author contributions

CES undertook a substantial portion of the investigative research, with significant contributions from KW, KMJdMS, and ADS, along with contributions from TTD, JAD, DMH, ZS, and AJW. KW, KMJdMS, CLW and AMB contributed to project conceptualisation. AMB, MPC, and CLW acquired funding and KW, AMB, and CLW provided supervision to PhD students. KW, CES, KMJdMS, and ADS wrote the original draft of the MS, with review and editing by KW, ADS, CLW and AMB.

## Conflicts of interest

There are no conflicts to declare.

## Supplementary Material

RA-015-D5RA02147B-s001

RA-015-D5RA02147B-s002

## Data Availability

See the ESI† for all experimental procedures. The sequences for *dcdo1* and DcDO1 can be found at accession PP598867 on NCBI. The castaneiolide BGC can be found at accession PV656010 on NCBI. Crystallographic data for 10 has been deposited at the CCDC under 2366244.
