# Supplementary material for: In vivo and in vitro investigations provide insights into maleidride biosynthesis in fungi
Source: RSC Adv. 2025 Jul 4;15(28):22799–806. doi: 10.1039/d5ra02147b (PMC12231587; doi:10.1039/d5ra02147b)
Supplement: RA-015-D5RA02147B-s001 [file RA-015-D5RA02147B-s001.pdf]

Supplementary Information (SI) for RSC Advances.
This journal is © The Royal Society of Chemistry 2025

Experimental and Supporting Information for:

In vivo and *in vitro* investigations provide insights into maleidride biosynthesis in fungi

Katherine Williams,†*[a,b] Catherine E. Spencer,†[c] Kate M. J. de Mattos-Shipley,†[a] Anjali D. Shah,†[a,c] Trong-Tuan Dao,[c,§] Jonathan A. Davies,[c] David M. Heard,[c] Zhongshu Song,[c] Ashley J. Winter,[c] Matthew P. Crump,[c] Christine L. Willis,[c] and Andrew M. Bailey*[a]

a. *School of Biological Sciences, Life Sciences Building, University of Bristol, 24 Tyndall Ave, Bristol BS8 1TQ, UK.*
b. *School of Applied Sciences, University of the West of England, Coldharbour Lane, Bristol, BS16 1QY.*
c. *School of Chemistry, University of Bristol, Cantock's Close, Bristol BS8 1TS, UK.*
§ *Department of Pharmacy, Duy Tan University, Danang 550000, Vietnam*

†Authors should be considered joint first authors
*Corresponding authors: Katherine12.Williams@uwe.ac.uk; Andy.Bailey@bristol.ac.uk

Table of Contents

Supplementary Figures 1

Supplementary Tables 2

1. General Procedures 3

2. NMR 3

3. Optical rotation data collection 3

4. HRMS 3

5. LC-MS 3

5.1. Analytical LC-MS 3

5.2. Preparative LC-MS 3

6. Strains 4

7. Fungal nucleic acid preparation 4

8. Genome sequencing and assembly 4

9. Bioinformatic annotation of castaneiolide BGCs 4

10. Growth and fermentation conditions 6

10.1. *Diffractella curvata* 6

10.2. *Scytalidium album* 6

10.3. *Macrophoma castaneicola* 6

11. Metabolite extraction procedures 6

11.1. *Diffractella curvata* 6

11.2. *Scytalidium album* 6

11.3. *Macrophoma castaneicola* 7

12. HPLC data 7

13. Gene deletions 8

13.1. *Scytalidium album* 8

13.1.1. ScyR4 8

13.1.2. ScyR2 8

13.1.3. ScyR11 9

13.2. *Diffractella curvata* 9

13.2.1. ZopL2 9

13.2.2. ZopL9 10

13.2.3. ZopR4 10

14. LC-MS analysis of deletion strains 11

15. Sequences 13

15.1. Accession numbers for relevant BGCs 13

15.2. Alignments 13

15.3. Percentage identity matrices 15
16. Enzyme expression and purification 15
16.1. General procedures 15
16.2. ZopL9 15
16.3. ZopL2 15
16.4. Analytical size exclusion experiment with ZopL9 and ZopL2 16
16.5. DcDO1 17
17. Enzyme assays 17
18. LC-MS analysis of enzyme assays 18
19. Synthesis 19
19.1. Synthesis of alkene 11 19
20. Isolated metabolites 22
20.1. Dihydro-5-hydroxy-deoxyscytalidin 9 22
20.2. Castaneiolide 10 28
20.3. Impure castaneiolide 10 from feeding alkene 11 to *D. curvata* Δ*zopPKS* 35
20.4. Dihydro-zopfiellin 13 36
21. X-ray crystallography 40
22. References 42

Supplementary Figures

Figure S1: Putative castaneiolide **10** BGC. 5
Figure S2: HPLC analysis of crude extract from *M. castaneicola* liquid culture in MEB 7
Figure S3: PCR analysis of the Δ*scyR4* transformants. 8
Figure S4: PCR analysis of the Δ*scyR2* transformants. 8
Figure S5: PCR analysis of the Δ*scyR11* transformants. 9
Figure S6: PCR analysis of the single Δ*zopL2* transformant. 9
Figure S7: PCR analysis of the single Δ*zopL9* transformant. 10
Figure S8: PCR analysis of the Δ*zopR4* transformants. 10
Figure S9: ELSD chromatogram analysis of crude extracts from maleidride BGC gene disruptions. 11
Figure S10: UV spectra (left) and mass spectra (right) of compound eluting at ~8 min RT from various *D. curvata* strains 12
Figure S11: Multiple sequence alignment using MUSCLE[15] of ZopL9 (QTE75983.1) compared to the homologue DcDO1 (WYX96522). 13
Figure S12: Multiple sequence alignment using MUSCLE[15] of ZopL2 (QTE75990.1), ScyR4 (QTE76004.1), and the modified sequence of TsRbtQ 14
Figure S13: Multiple sequence alignment using MUSCLE[15] of ScyL2 (QTE75998.1), ZopL9 (QTE75983.1), CasL3 and CasL8. 14
Figure S14:**A)** SDS-PAGE and **B)** MS characterisation of ZopL2. 15
Figure S15: Size Exclusion analysis of purified ZopL9 (pink), ZopL2 (blue), and an equimolar mixture of both proteins (black). 16
Figure S16: SDS-PAGE analysis of DcDO1. 17
Figure S17:EIC ES^- (5-95% MeCN:H_2O gradient) from enzyme assays with ZopL9, ZopL2, DcDO1 and deoxyscytalidin **5**. 18
Figure S18: ^{1}H NMR spectrum (400 MHz, $CDCl_3$) of **11**. 20
Figure S19: ^{13}C NMR spectrum (125 MHz, $CDCl_3$) of **11**. 20
Figure S20: HSQC spectrum (400 MHz, $CDCl_3$) of **11**. 21
Figure S21: HMBC spectrum (400 MHz, $CDCl_3$) of **11**. 21
Figure S22: Key HMBC correlations of **9**. 23
Figure S23: Key NOESY correlations of **9**. 24
Figure S24: ^{1}H NMR spectrum (500 MHz, $CDCl_3$) of **9**. 24
Figure S25: ^{1}H NMR spectrum (500 MHz, $CDCl_3$, drop D_2O) of **9**. 25
Figure S26: ^{13}C NMR spectrum (125 MHz, $CDCl_3$) of **9**. 25
Figure S27: COSY spectrum of **9**. 26
Figure S28: HSQC spectrum of **9**. 26
Figure S29: HMBC spectrum of **9**. 27
Figure S30: ADEQUATE spectrum of **9**. 27
Figure S31: NOESY spectrum of **9**. 28
Figure S32: ^{1}H NMR spectrum (600 MHz, $(CD_3)_2CO$) of **10.** 30
Figure S33: ^{13}C NMR spectrum (313 K, 151 MHz, $(CD_3)_2CO$) of **10.** 30
Figure S34: COSY spectrum of **10**. 31
Figure S35: HSQC spectrum of **10**. 31
Figure S36: HMBC spectrum of **10**. 32
Figure S37: NOESY spectrum of **10**. 32
Figure S38: Irradiated NOESY spectrum of **10**. 33
Figure S39: Key NOESY correlations of **10**. 33
Figure S40: Stacked ^{1}H NMR spectra of **10** 34

Figure S41: Stacked ^{13}C NMR spectra of **10** 34
Figure S42: Diode array traces (5-95% MeCN:H_2O gradient) for *D. curvata* Δ*zopPKS* and the Δ*zopPKS* strain fed with alkene **11** 35
Figure S43: Comparison of the ^{1}H spectra (400 MHz, $CDCl_3$) for castaneiolide **10**........ 35
Figure S44: Diode array chromatograms (5-95% MeCN:H_2O gradient) for WT *D. curvata* and the genetically impure Δ*zopR4*† strains........ 36
Figure S45: UV and ES^- spectra of dihydro-zopfiellin **13**. 36
Figure S46: Key HMBC correlations used to aid the assignment of dihydro-zopfiellin **13**. 37
Figure S47: ^{1}H NMR (500 MHz, $CDCl_3$) spectrum of **13**. 38
Figure S48: ^{13}C NMR (125 MHz, $CDCl_3$) spectrum of **13**. 38
Figure S49: Comparison of the ^{13}C spectra ($CDCl_3$, 125 MHz) of zopfiellin **4** (top) and dihydro-zopfiellin **13** (bottom). 39
Figure S50: Comparison of labelled castaneiolide **10** with the previously proposed maleidride numbering system (left) and X-ray structure numbering system (right). 41

Supplementary Tables

Table S1: Predicted function of genes located within the scaffold hypothesised to encode the castaneiolide **10** biosynthetic gene cluster........ 5
Table S2: Genbank and MiBIG accession numbers for BGCs discussed within the manuscript........ 13
Table S3: Percentage identity matrix for AsaB-like αKGDDs in the scytalidin, zopfiellin and castaneiolide BGCs........ 15
Table S4: NMR assignments for dihydro-5-hyroxy-deoxyscytalidin **9** ([a]125MHz, [b]500 MHz in $CDCl_3$). 23
Table S5: Comparison of NMR data for castaneiolide **10** isolated from *M. castaneicola* against castaneiolide **10** isolated from feeding **7** to *D. curvata* Δ*zopPKS* 29
Table S6: NMR assignments for dihydro-zopfiellin **13** ([a]125 MHz, [b]500 MHz in $CDCl_3$)........ 37
Table S7: Crystal data and structure refinement for castaneiolide **10** 40

1. General Procedures

Molecular biology kits were used according to manufacturer's protocols. PCR was carried out using KAPA-Hifi (Roche), DreamTaq (Thermo Fisher Scientific) and BioMix Red (Bioline). Restriction endonucleases were purchased from Thermo Fisher Scientific.

2. NMR

Instruments used: Varian 400-MR (400MHz), Varian VNMRS500 (500MHz), Bruker 500 Cryo (500MHz) or Varian VNMRS600 Cryo (600MHz). Chemical shifts (δ) quoted in parts per million (ppm) and coupling constants (J) in Hertz (Hz), rounded to 0.5 Hz intervals. Two-dimensional NMR techniques (HSQC, COSY, HMBC) were used routinely for the assignment of structures. Use of NOESY and TOCSY techniques is indicated as appropriate, and the identified correlations tabulated. Residual solvent peaks were used as the internal reference for proton and carbon chemical shifts.

3. Optical rotation data collection

Specific rotations ($[\alpha]_D^T$) were measured on a Bellingham and Stanley Ltd. ADP220 polarimeter and are quoted in (° ml) (g dm)$^{-1}$.

4. HRMS

(1) Bruker Daltonics micrOTOF II, (2) Bruker Daltonics Apex IV FT-ICR instruments.

5. LC-MS

5.1. Analytical LC-MS

All crude extracts were prepared to a concentration of 10 mg/ml in HPLC grade acetonitrile and placed in LC-MS vials. 20 µl of the extracts were injected and analysed using Waters 2445 SFO HPLC; Waters 2767 autosampler, Phenomenex Kinetex column (2.6 µ, C_{18}, 100 Å, 4.6 × 100 mm) equipped with a Phenomenex Security Guard precolumn (Luna C5 300 Å) eluted at 1 mL/min. Detection was achieved by Waters 2998 diode array detector between 200 and 400 nm; Waters Quattro Micro ESI mass spectrometer in ES^+ and ES^- modes between 100 *m/z* and 1000 *m/z*; Waters 2424 ELS detector. Solvents were: A, HPLC grade H_2O containing 0.05% formic acid; B, HPLC grade CH_3CN containing 0.05% formic acid. Samples were run on a 5 – 95% or 40 – 95% CH_3CN gradient over 20 minutes comprising: 0 min, 5% B; 2 min, 5% B; 15 min, 95% B; 17 min, 95% B; 18 min, 5% B; 20 min, 5% B; or 0 min, 5% B; 2 min, 40% B; 15 min, 95% B; 17 min, 95% B; 18 min, 5% B; 20 min, 5% B; respectively, flow rate 1 mL/min.

5.2. Preparative LC-MS

Compounds were purified using a Waters time-directed autopurification system compromising Waters 2767 autosampler, Waters 2545 pump system, Phenomenex Kinetex column (5 µ, C_{18}, 100 Å, 250 × 21.20 mm) equipped with Phenomenex Security Guard precolumn (Luna C5 300 Å) eluted at 16 mL/min. Solvents were: A, HPLC grade H_2O containing 0.05% formic acid; B, HPLC grade MeOH containing 0.045% formic acid; and C, HPLC grade CH_3CN containing 0.045% formic acid. The post column was split (100:1) with the minority flow made up with HPLC grade MeOH containing formic acid 0.045% to 1 mL/min for simultaneous analysis by Waters 2998 diode array detector between 200 and 400 nm; Waters Quattro Micro ESI mass spectrometer in ES^+ and ES^- modes between 100 *m/z* and 1000 *m/z*; Waters 2424 ELS detector. Metabolites were collected into glass test tubes. Combined samples were evaporated under N_2 gas, weighed and dissolved in a suitable solvent for future applications.

6. Strains

Diffractella curvata CBS 591.74 was obtained from the CBS collection; *Scytalidium album* strain UAMH 3620 from the University of Alberta Mold Herbarium and culture collection; *Macrophoma castaneicola* M1-48 was obtained from the NARO Genebank collection. *Saccharomyces cerevisiae* (Stratagene) strain YPH499 was used for plasmid assembly by yeast homologous recombination. *Escherichia coli* strain TOP10 (Invitrogen) was used as a host for all plasmids, except where stated otherwise.

7. Fungal nucleic acid preparation

Fungi were grown in an appropriate liquid medium, freeze dried and then ground under liquid nitrogen. DNA extraction buffer (4 mL) and buffered phenol (4 mL) were added to the fungus and ground further. Once liquefied, choloroform:isoamyl alcohol (4 mL) was added, vortexed and centrifuged at 10,000 g for 10 min. An equal volume of 4M LiCl added to the aqueous layer, vortexed and left at 4 °C overnight. After centrifugation, isopropanol (1:1) was added to the supernatant and centrifuged again. The pellet was washed with 70% ethanol (5 mL) and the DNA suspended in TE (100 µL).

8. Genome sequencing and assembly

Short read paired end 250 bp reads were generated using an Illumina MiSeq.

Long read data was generated using Oxford Nanopore Technologies. Sequencing libraries of genomic DNA were prepared using Ligation Sequencing Kit V14 (Oxford Nanopore Technologies, SQK-LSK114) following standard manufacturers protocols. No additional fragmentation steps were included to ensure long-reads were achieved. Libraries were loaded onto a R10.4.1 flow cells (Oxford Nanopore Technologies, FLO-MIN114) and sequencing performed on a MinION Mk1B device using MinKNOW version 22.12.7.[1] Raw nanopore sequencing data in POD5 format was basecalled using dorado version 0.8[2] and the 'dna_r10.4.1_e8.2_400bps_hac@v5.0.0' model. This produced unaligned BAM files that were subsequently converted to FASTQ format using samtools version 1.21[3] for downstream analysis.

Both the short and long read data were used to generate a hybrid assembly with MaSurRCA v4.1.0.[4] Before annotation the repeats in the genome were softmasked with RepeatMasker v4.1.7-p1[5] using a repeat library generated with RepeatModeler v2.0.6.[6] The genome assembly was annotated with Braker v3.0.8,[7] using all reviewed Ascomycota proteins from UniProt (https://www.uniprot.org/, downloaded on 5.12.2024) as protein hints. The completeness of the genome assembly and annotation were assessed using BUSCO v5.8.1[8] with the ascomycota_odb10 gene set (1706 orthologs).

Quality of assembly and annotation

The hybrid assembly with MaSurRCA.[4] resulted in 147 scaffolds with a total size of 55,432,448 base pairs and an N50 of 1,022,105. The Braker3 annotation resulted in 13,846 proteins, with an average length of 483.84 amino acids.

A BUSCO[8] completeness assessment found 99.4% of the ascomycota_odb10 gene set complete in the assembled genome (C:99.4%[S:95.9%,D:3.5%],F:0.1%,M:0.6%) and 99.4% complete in the annotated proteome (C:99.4%[S:93.3%,D:6.2%],F:0.1%,M:0.5%), suggesting a high completeness of both the genome assembly and the annotation.

9. Bioinformatic annotation of castaneiolide BGCs

Co-located maleidride genes were identified from the genome of *M. castaneicola* on a 1.8Mb scaffold by using MultiGeneBlast.[9] The core maleidride *P. fulvus* sequences from the byssochlamic acid BGC

were used as queries.[10] [11] The Braker v3.0.8 annotation was utilised to further functionally annotate the putative maleidride BGC from the predicted translation, using BLAST[12] and Interpro.[13] Where obvious errors in intron/exon boundaries were identified, the boundaries were manually edited. See Figure S1 and Table S1 for predicted functions for each of the genes identified within the BGC, and the identified homologues.

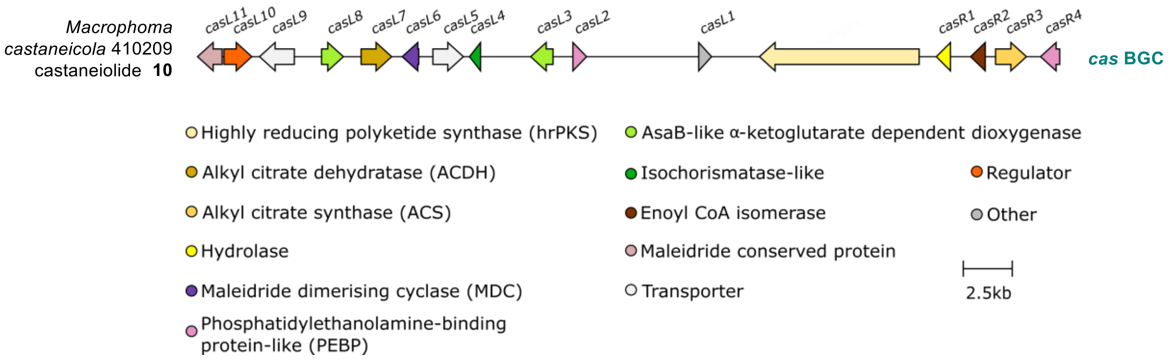

Figure S1: Putative castaneiolide 10 BGC.

Table S1: Predicted function of genes located within the scaffold hypothesised to encode the castaneiolide **10** biosynthetic gene cluster (found at accession number PV656010). Homologues were identified using Swissprot database using each predicted protein sequence as a query (BLASTp). * denotes instances where homologues were identified within the NCBI nr (nonredundant), rather than Swissprot, database. † See reference [11] for an explanation of the distinction between the different types of maleidride PEBP-like encoding genes.

Gene	Accession no.	Putative function	Homologue	Identity	Query coverage	E value
casL11	XUB15300	maleidride conserved protein	* ZopL8 **QTE75984.1**	61.24%	54%	$2e^{-63}$
casL10	XUB15301	transcription factor	phiQ **A0A348HAZ0.1**	27.08%	95%	$2e^{-37}$
casL9	XUB15302	MFS-type transporter	phiD **A0A348HAX9.1**	50.71%	97%	0
casL8	XUB15303	α-ketoglutarate dependent dioxygenase	phiK **A0A348HAY6.1**	50.00%	99%	$4e^{-86}$
casL7	XUB15304	alkylcitrate dehydratase	phiI **A0A348HAY4.1**	57.38%	97%	0
casL6	XUB15305	maleidride dimerising cyclase	tstC **B8MKZ0.1**	44.07%	67%	$2e^{-47}$
casL5	XUB15306	MFS-type transporter	phiL **B8MKZ7.1**	49.90%	95%	$3e^{-159}$
casL4	XUB15307	isochorismatase-like	CA_P0030 **Q97TR7.1**	27.37%	99%	$7e^{-9}$
casL3	XUB15308	α-ketoglutarate dependent dioxygenase	CTB9 **A0A2G5I8W0.1**	34.14%	78%	$4e^{-25}$
casL2	XUB15309	phosphatidyl ethanolamine-binding protein-like type 1 †	phiN **A0A348HAY9.1**	42.52%	59%	$4e^{-26}$
casL1	XUB15310	hypothetical protein	* helix-turn-helix, psq domain-containing protein **XP_044726392.1**	59.12%	100%	$2e^{-57}$
caspks	XUB15311	highly reducing polyketide synthase	tstA **B8MKY6.1**	53.40%	98%	0
casR1	XUB15312	hydrolase	phiM **B8MKZ6.1**	46.72%	100%	$3e^{-63}$
casR2	XUB15313	enoyl coA isomerase-like	dienoyl-CoA isomerase **Q54SS0.1**	26.85%	46%	$5e^{-04}$
casR3	XUB15314	alkylcitrate synthase	phiJ **A0A348HAY5.1**	57.93%	98%	$1e^{-176}$
casR4	XUB15315	phosphatidyl ethanolamine-binding protein-like type 2 †	Phomoidride biosynthesis cluster protein B **B8MKY9.1**	42.86%	74%	$6e^{-53}$
casR5	XUB15316	hypothetical protein	* hypothetical protein **KAK7735897.1**	40.57%	49%	$7e^{-27}$
casR6	XUB15317	oxidoreductase	BDH **H9XP47.1**	31.05%	94%	$2e^{-16}$
casR7	XUB15318	phosphonomutase	CPEP phosphonomutase **P11435.3**	39.22%	93%	$6e^{-56}$

10. Growth and fermentation conditions

10.1. *Diffractella curvata*

D. curvata was maintained on PDA at 25 °C. Agar plugs were used to inoculate 100 mL PDB in 500 mL non-baffled Erlenmeyer flasks and grown at 25 °C with shaking at 200 rpm. After growing for 1 week, the seed culture was homogenised and used to inoculate flasks containing fresh PDB. For metabolite production the cultures were grown for 8 days. The homogenised *D. curvata* seed culture was also used to inoculated static rice cultures (50 g rice and 50 ml water autoclaved in 500 mL Erlenmeyer flasks) which were grown for 21 – 28 days at 25 °C.

10.2. *Scytalidium album*

S. album was maintained on MEA at 25 °C. Agar plugs were used to inoculate 100 mL GNB in 500 mL non-baffled Erlenmeyer flasks and grown at 25 °C with shaking at 200 rpm. After growing for 1 week, the seed culture was homogenised and used to inoculate flasks containing MEB. For metabolite production the cultures were grown for 12 days. The homogenised *S. album* seed culture was also used to inoculate static rice- or oat-based cultures (50 g rice/oats and 50 ml of water autoclaved in 500 mL Erlenmeyer flasks) which were grown for 21 – 28 days at 25 °C.

10.3. *Macrophoma castaneicola*

M. castaneicola was maintained on PDA at 28 °C. For castaneiolide **10** production, a liquid starter culture was produced by inoculation of the mycelia from one PDA culture into 50 mL PDB, in a 250 mL non-baffled conical flask. This was cultured for 6 days at 28 °C with shaking at 200 rpm. The starter culture was homogenised, and 100 mL was inoculated into 500 mL MEB in a 2.5 L baffled shake flask. This flask was then cultured at 28 °C with shaking at 200 rpm for 24 days.

11. Metabolite extraction procedures

11.1. *Diffractella curvata*

For liquid cultures, the cultures were extracted after 8 days. To extract the cultures were acidified to pH 3 using 2 M HCl and the mycelia and broth were blended. The mycelia were removed by filtering and an equal volume of EtOAc was added to the broth. The aqueous layer was washed a further two times with EtOAc. The organic extracts were combined, dried with $MgSO_4$ and concentrated under vacuum. For *D. curvata* grown on rice, after 21 – 28 days rice was soaked in EtOAc (500 mL), blended and acidified using HCl. Water was added and then the organic and aqueous layers separated. The aqueous layer was washed a further two times with EtOAc. The combined organic layers were dried with $MgSO_4$ and then concentrated under reduced pressure. The crude organic extract was dissolved in acetonitrile and defatted with hexane.

11.2. *Scytalidium album*

For liquid cultures, the culture was extracted after 12 days by acidifying and then blending. The broth was extracted using an equal volume of EtOAc. The combined organic extracts were dried with $MgSO_4$ and concentrated under reduced pressure. For *S. album* grown on rice or oats, after 21 – 28 days, the rice or oats were soaked in EtOAc (500 mL), blended and acidified to pH 3 using HCl. Water was added and the organic and aqueous layers separated. The aqueous layer was washed a further two times with EtOAc. The combined organic layers were dried with $MgSO_4$ and then concentrated under reduced pressure. The crude organic extract was dissolved in acetonitrile and defatted by washing with hexane.

11.3. *Macrophoma castaneicola*

The 500 mL culture was mixed with an equal volume of EtOAc under acidic conditions, then blended and filtered, and the organic phase separated. The aqueous phase was re-extracted twice more with 500 mL EtOAc. The combined organic phases were dried with $MgSO_4$ and then concentrated under reduced pressure. 32.6 mg castaneiolide **10** was purified from the resultant crude extract.

12. HPLC data

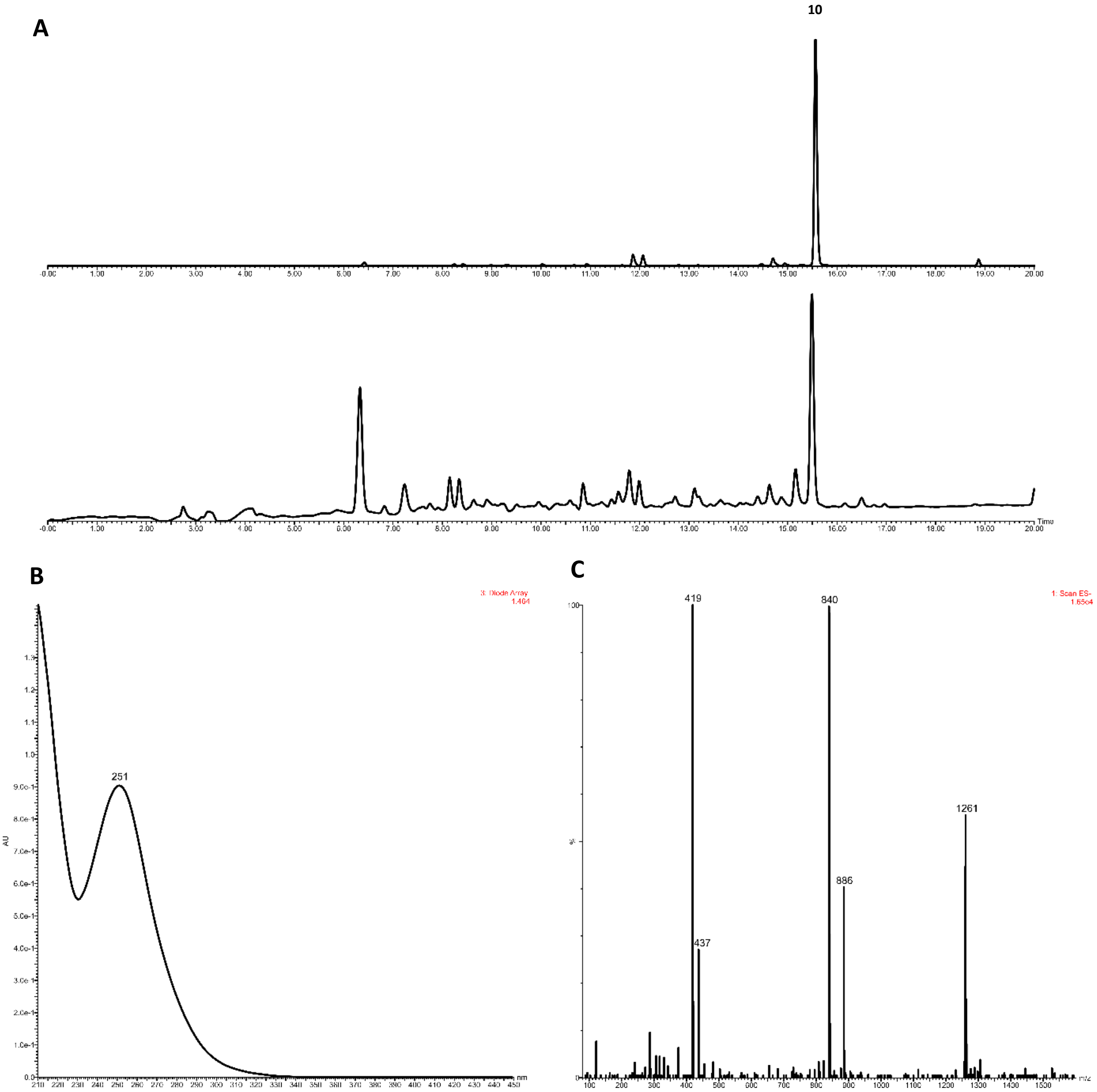

Figure S2: HPLC analysis of crude extract from *M. castaneicola* liquid culture in MEB (500 mL), analysed with an HPLC-UV/MS/ELSD system (gradient 5-95% $CH_3CH:H_2O$). Castaneiolide **10** can be seen eluting at 15.5 minutes. **(A)** Top: ELSD chromatogram, Bottom: Diode array chromatogram; **(B)** UV spectrum; **(C)** ES^- spectrum.

13. Gene deletions

13.1. *Scytalidium album*

13.1.1. ScyR4

The gene *scyR4* which encodes an isochorismatase-like enzyme was deleted using the bipartite KO approach.[14] PCR analysis (Figure S3) showed that six of the seven transformants obtained were genetically pure with confirmed targeting in transformants T1, 2, 3, 5, 6 and 7.

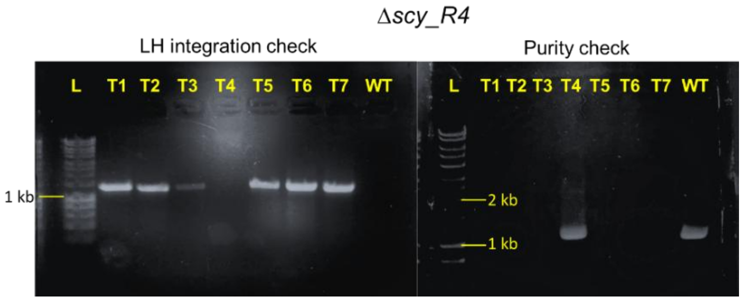

Figure S3: PCR analysis of the Δ*scyR4* transformants. Analysis to confirm the integration of the hygromycin resistance cassette and genetic purity. Expected band size for LH integration check = 1329 bp, expected band size for purity check = 1049 bp.

13.1.2. ScyR2

The gene *scyR2* which encodes an enoyl CoA isomerase-like enzyme was deleted using the bipartite KO approach.[14] PCR analysis showed that of the seven transformants analysed, three (T1, 2 and 3) had both confirmed integration at the correct locus and were free of the non-disrupted allele (Figure S4).

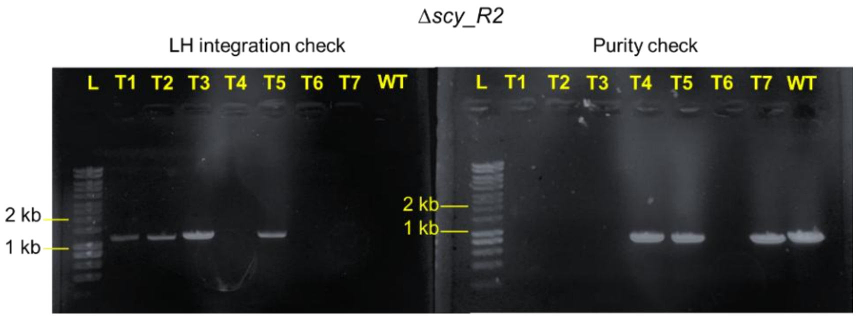

Figure S4: PCR analysis of the Δ*scyR2* transformants. Analysis to confirm the integration of the hygromycin resistance cassette and genetic purity. Expected band size for LH integration check = 1185 bp, expected band size for purity check = 919 bp.

13.1.3. ScyR11

The gene *scyR11* which encodes a maleidride conserved protein was deleted using the bipartite KO approach.[14] PCR analysis showed that five transformants had confirmed integration at the correct locus, and were free of the WT allele (T1, 2, 3, 5, 7 - Figure S5).

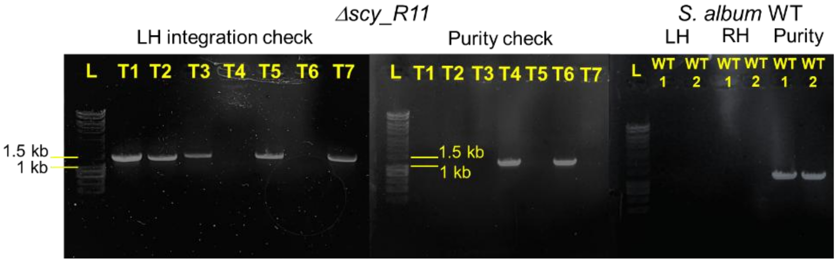

Figure S5: PCR analysis of the Δ*scyR11* transformants. Analysis to confirm the integration of the hygromycin resistance cassette and genetic purity. Expected band size for LH integration check = 1408 bp, expected band size for purity check = 1162 bp.

13.2. *Diffractella curvata*

13.2.1. ZopL2

The gene *zopL2* which encodes an isochorismatase-like enzyme was deleted using the bipartite KO approach.[14] PCR analysis confirmed a genetically pure disruption (Figure S6), subsequently, the transformant was sequenced over the deletion which confirmed that the correct strain (*D. curvata* Δ*zopL2*) had been isolated.

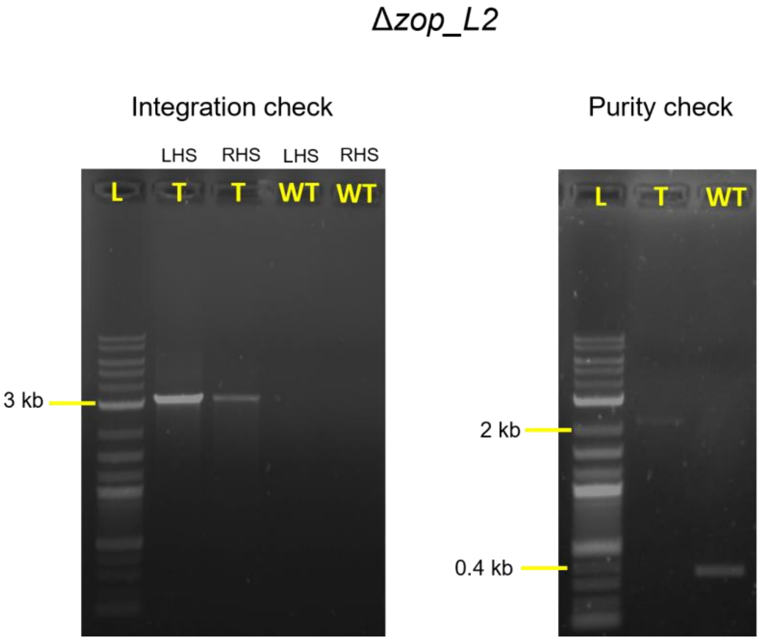

Figure S6: PCR analysis of the single Δ*zopL2* transformant. Analysis to confirm the integration of the hygromycin resistance cassette and genetic purity. Expected band size for LHS integration check = 3000 bp, expected band size for RHS integration check = 3000 bp, expected band size for purity check = 348 bp.

13.2.2. ZopL9

The gene *zopL9* which encodes an αKGDD enzyme was deleted using the bipartite KO approach.[14] PCR analysis of the single transformant confirmed integration at the correct locus and is free of the WT allele (T - Figure S7). The presence of the ~4.5 kb amplification product in the purity check of the transformant indicates amplification over the hygromycin resistance cassette. No ~1.2 kb product was observed, demonstrating genetic purity for this strain. Subsequently, the single transformant was sequenced over the deletion which confirmed that the correct strain (*D. curvata* Δ*zopL9*) had been isolated.

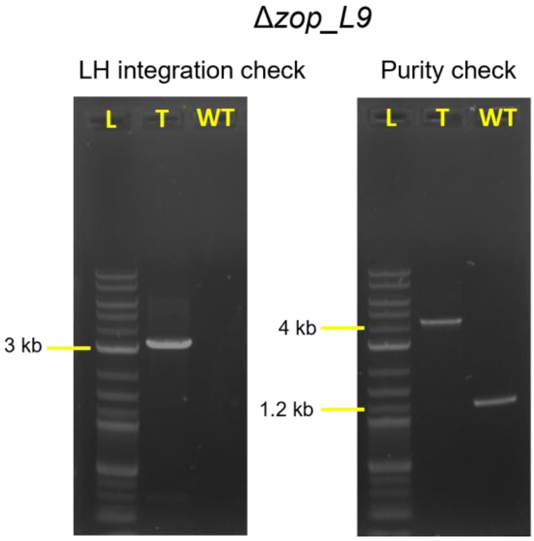

Figure S7: PCR analysis of the single Δ*zopL9* transformant. Analysis to confirm the integration of the hygromycin resistance cassette and genetic purity. Expected band size for LH integration check = 3000 bp, expected band size for purity check = 1242 bp.

13.2.3. ZopR4

Deletion of the *zopR4* gene was attempted using the bipartite KO approach.[14] PCR analysis indicated the two deletion strains generated were not genetically pure as a faint band could be seen in the purity check (Figure S8). Further rounds of sub-culturing on selective plates failed to yield pure deletion strains.

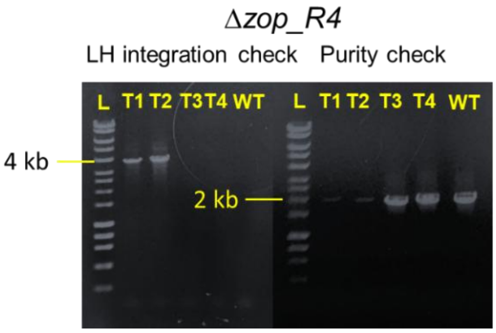

Figure S8: PCR analysis of the Δ*zopR4* transformants. Analysis showed that two transformants contained the hygromycin resistance cassette, however faint bands in the purity check show that these transformants were not genetically pure. Expected band size for LH integration check = 3881 bp, expected band size for purity check = 1969 bp.

14. LC-MS analysis of deletion strains

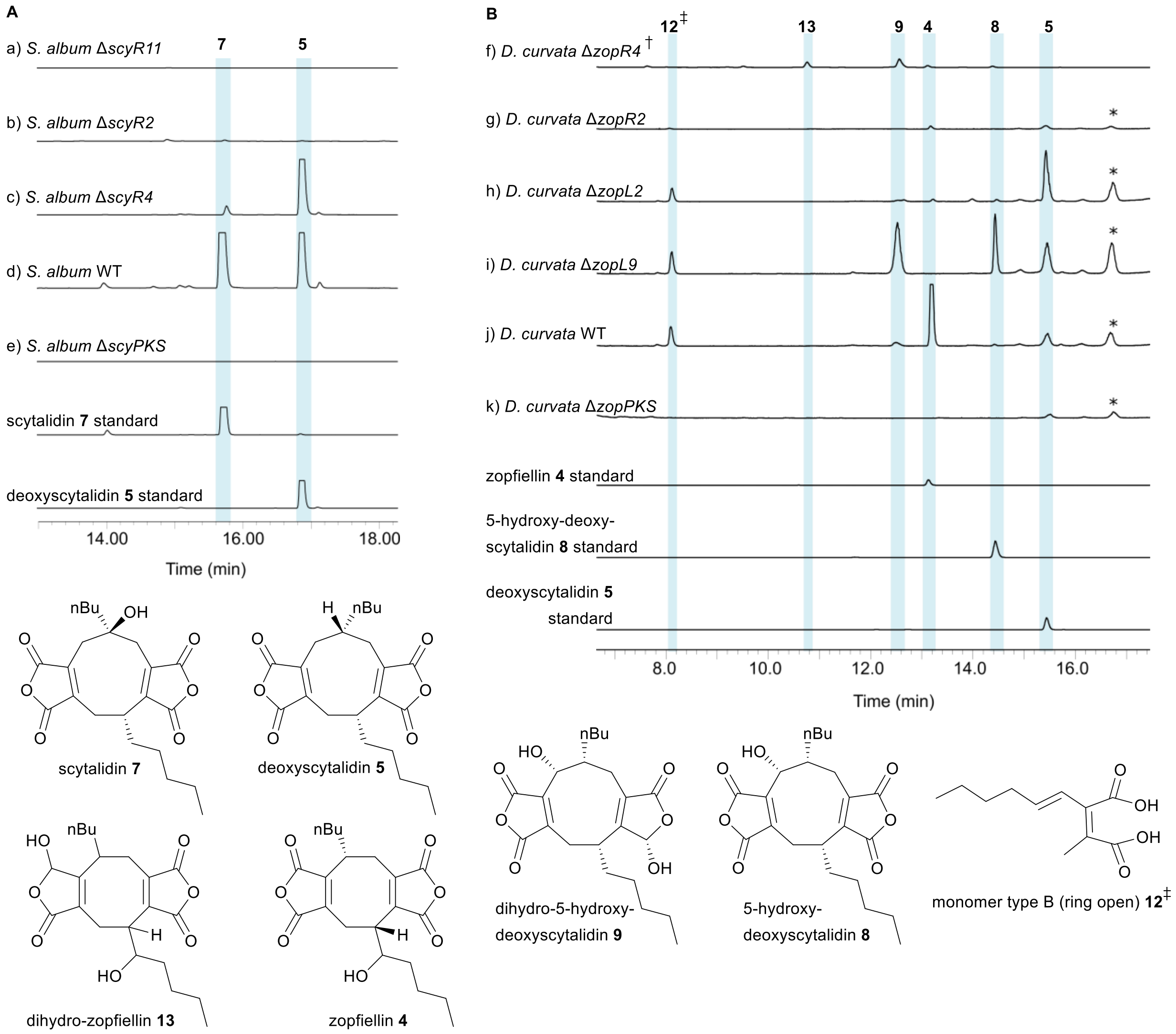

Figure S9: ELSD chromatogram analysis of crude extracts from maleidride BGC gene disruptions. **A)** ELSD chromatograms of gene disruptions in *S. album* (5-95% MeCN:H_2O gradient). Trace **(a)** Deletion of the gene encoding the maleidride conserved protein, *scyR11* appears to completely abolish scytalidin **7** biosynthesis. Trace **(b)** Deletion of the enoyl CoA isomerase, *scyR2* significantly reduces scytalidin **7** biosynthesis. Trace **(c)** Deletion of the ICM-like hydrolase gene, *scyR4* reduces production of the mature maleidride scytalidin **7** and leads to the accumulation of the intermediate, deoxyscytalidin **5**. **B**) ELSD chromatograms of gene disruptions in *D. curvata* (40-95% MeCN:H_2O gradient). Trace **(f)** Deletion of the oxidoreductase encoding gene, *zopR4*† (incomplete genetic purity) reduces production of the mature maleidride zopfiellin **4** leads to accumulation of dihydro-zopfiellin **13**, and dihydro-5-hydroxy-deoxyscytalidin **9**. Trace **(g)** Deletion of the enoyl CoA isomerase, *zopR2* significantly reduces zopfiellin **4** biosynthesis, and also appears to impact production of the likely zopfiellin monomer **12**‡. Trace **(h)** Deletion of the ICM-like hydrolase gene, *zopL2* reduces production of the mature maleidride zopfiellin **4** and leads to the accumulation of the intermediate, deoxyscytalidin **5**. Trace **(i)** Deletion of the αKGDD encoding gene, *zopL9* leads to a loss of zopfiellin **4** production with the accumulation of 5-hydroxy-deoxyscytalidin **8** and the reduced compound **9**. *unrelated metabolite. ‡ putative compound predicted from MS and UV data.

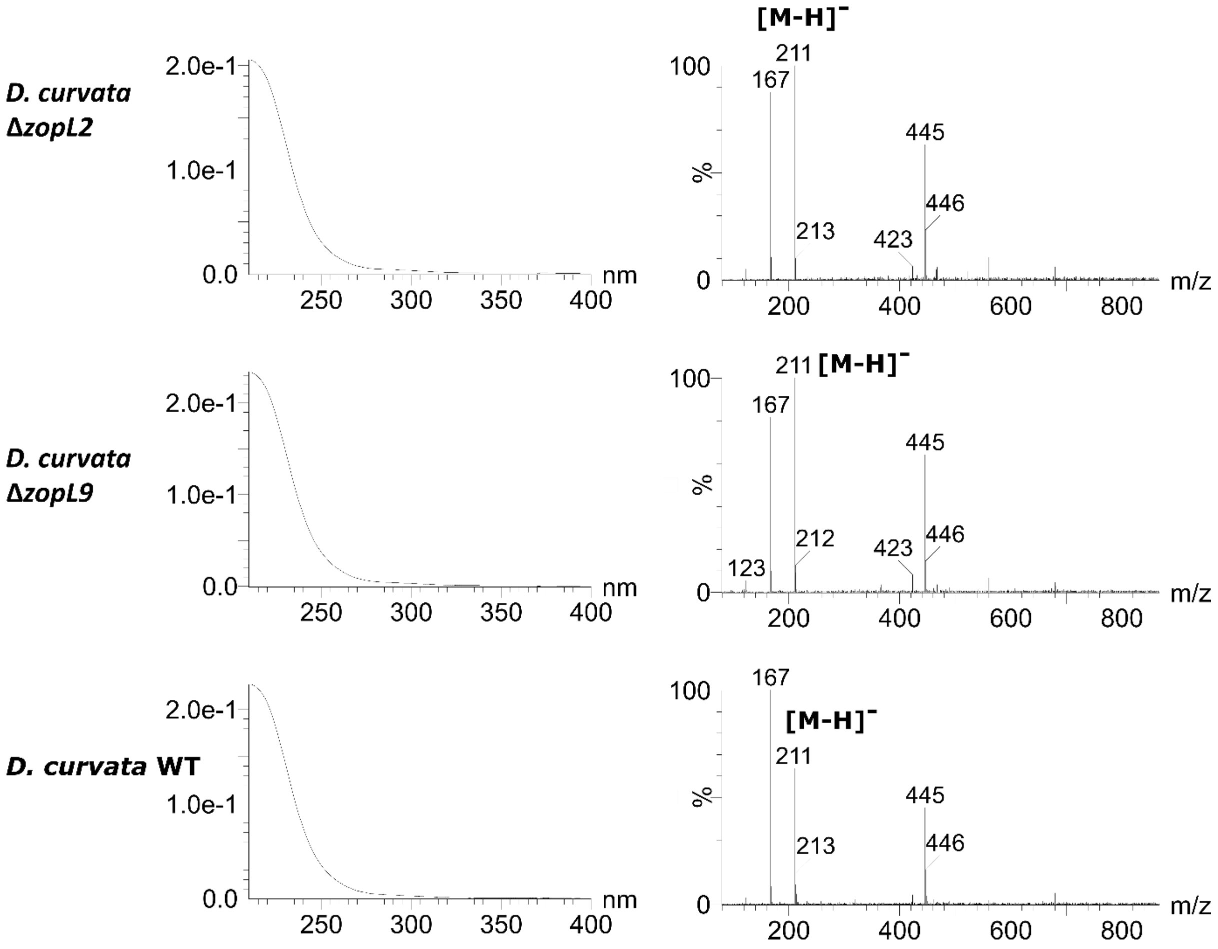

Figure S10: UV spectra (left) and mass spectra (right) of compound eluting at ~8 min RT from various *D. curvata* strains as seen in Figure S9B, traces h, i and j. Compound predicted to be ring open tetraketide monomer 12.

15. Sequences

15.1. Accession numbers for relevant BGCs

Table S2: Genbank and MiBIG accession numbers for BGCs discussed within the manuscript

Biosynthetic gene cluster	Genbank accession number	MiBIG accession number
**Zopfiellin BGC**	LC516887.1	BGC0002222
**Scytalidin BGC**	MT724050.1	BGC0002223
**Castaneiolide BGC**	PV656010	n/a

```
>ZopL2 (His-tag/ linker underlined)

MHHHHHHGKPIPNPLLGLDSTENLYFQGIDPFTMAKTALLVMDVQGAFVPRLAQSSDYLPRLAKTIAA
ARPSVVKVIYTRVAFRPGHPEISPSNPTFSAAVKSKSFVEGSPETLIDPSIAPQEGDVLVDKKRVSAF
SGSGLDIILSSLGIETVVLAGMSTGGVVLSTVLEASDKDFGVVVLKDLCVDADETLHNTLMDKIFTKR
GQVVEAEKWLETLKA
```

Molecular weight 23583.16

```
>DcDO1 (His-tag/ linker underlined)

MHHHHHHGKPIPNPLLGLDSTENLYFQGIDPFTMSMTIDMPPTVIHTTVDYYVPPAGQKEHGIGDDDF
EEQYGNKNVVHYPIKLRDMRTGGFTLEKNGFQLVPYQSKLTAELDLDTIKKVYFPELMAAIKKVTGAS
EVHIILPRFRDTSTEKSGFKKGWNYNNGPVRRSHIDISPSGIEETLIPILGEEYLKSIAGRWKFVNAW
RPTRLVERDPLAVCHKVPEEDLIALERTIPGKALKQGRYLVKAGDHEWYFAPNQQPDELLLFTQYSDF
PDRGLADRVVHASVMLPGTEDKPARTSIEARVIVVW
```

Molecular weight 35266.23

15.2. Alignments

CLUSTAL multiple sequence alignment by MUSCLE (3.8)

```
WYX96522_DcDO1     -MSMTIDMPPTVIHTTVDYYVPPAGQKEHGIGDDDFEEQYGNKNVVHYPIKLRDMRTGGF
QTE75983.1_ZopL9   MATATVTTAPTVVRTTADYYDAPPVLKIHTYTRESYEEQFGNKSVIHHPINLKDIRAANI
                     : *:  .***:.**.*** .*.  * *    :.:***:***.*:*:**:*.*:*:..:

WYX96522_DcDO1     TLEKNGFQLVPYQSKLTAE---LDLDTIKKVYFPELMAAIKKVTGASEVHIILPRFRDTS
QTE75983.1_ZopL9   NLQNNGFQLIKLQSKLTNPDDYLDEETVKRVYIPELAEAVKKLTGATEVRVLNPKVRDSS
                   .*::*****:  *****     ** :*:*.**:***  *:**:***:**.:: *..**:*

WYX96522_DcDO1     TEKSGFKKGWNYNNGPVRRSHIDISPSGIEETLIPILGEEYLKSIAGRWKFVNAWRPTRL
QTE75983.1_ZopL9   TEKDGFENNWGKNNGAVRRIHIDLAPGGVEEALYPIFGEEYMKSIAGRWRLINAWKPTRP
                   ***.**::.*. ***.*** ***::*.*:**:* **:****:*******.::***.***

WYX96522_DcDO1     VERDPLAVCHKVPEEDLIALERTIPGKALKQGRYLVKAG--DHEWYFAPNQQPDELLLFT
QTE75983.1_ZopL9   VERDPLAVCDRVPDEDLVPLQRVVPGKALMEQRYHLKTGKKDHDWYYASNQQPDEVLLFT
                   ********* .**:***:.*:*.:***** : ** :*:*  **:**:*.******:****

WYX96522_DcDO1     QYSDFPDRGLADRVVHASVMLPGTEDKPARTSIEARVIVVW
QTE75983.1_ZopL9   QYSDFPNRNTADRVPHVSVKLPGQEDKPRRTSVDARCLVVW
                   ******:*. **** *.** *** **** ***::** :***
```

Figure S11: Multiple sequence alignment using MUSCLE[15] of ZopL9 (QTE75983.1) compared to the homologue DcDO1 (WYX96522). An asterisk (*) marks completely conserved amino acid residues, whereas a colon (:) identifies highly conserved residues and a period (.) indicates moderately conserved residues. The two sequences show 60% identity.

CLUSTAL multiple sequence alignment by MUSCLE (3.8)

```
QTE75990.1_ZopL2       MAKTALLVMDVQGAFVPRLAQSSDYLPRLAKTIAAARPSVVKVIYTRVAFRPGHPEISPS
EED18834.1mod-TsRbtQ   MSKTALLVMDYQAGIISRLSLPENHLQLLANTIDTARP-YAKIIYVTVAFRPGHPEVSAS
QTE76004.1_ScyR4       MDKTALLVMDVQGGMVSRLPTAQTFIPLLSKTVAAARP-FVKIIYATVSFRPGHPEIAPS
                       * ******** *..::.**. .. .:  *::*: :***  .*:**. *:*******::.*

QTE75990.1_ZopL2       NPTFSAAVKSKSFVEGSPETLIDPSIAPQEGDVLVDKKRVSAFSGSGLDIILSSLGIETV
EED18834.1mod-TsRbtQ   NATFSAAAKSNSFVSGSPETQIDPVIAPKEGDILIEKKRVSAFTGSGLDLVLKGLGVETL
QTE76004.1_ScyR4       NVIFSAALKSGAFVAGSPETVIDPSIAPQEGDILVEKKRVSAFAGSGLDVILRGLGIETL
                       *  **** ** :** ***** *** ***:***:*::*******:*****::* .**:**:

QTE75990.1_ZopL2       VLAGMSTGGVVLSTVLEASDKDFGVVVLKDLCVDADETLHNTLMDKIFTKRGQVVEAEKW
EED18834.1mod-TsRbtQ   VLAGISTGGVVLSTVCEAADKDFKLVVLKDLCVDGDEKLHNGLMSKIFSKRGEVLGAEEW
QTE76004.1_ScyR4       VLTGISTGGVVLSTVCEAADKDFKLVILKDLCADPDQAVHNVLMENVFTKRGEVLGAEEW
                       **:*:********** **:**** :*:*****.* *: :** **.::*:***:*: **:*

QTE75990.1_ZopL2       LETLKA
EED18834.1mod-TsRbtQ   LQKLKA
QTE76004.1_ScyR4       LEKLKA
                       *:.***
```

Figure S12: Multiple sequence alignment using MUSCLE[15] of ZopL2 (QTE75990.1), ScyR4 (QTE76004.1), and the modified sequence of TsRbtQ (EED18834.1mod, a read-through intron has been removed – see [11] for details). An asterisk (*) marks completely conserved amino acid residues, whereas a colon (:) identifies highly conserved residues and a period (.) indicates moderately conserved residues. TsRbtQ shows 68.65% identity with ZopL2, and 71.35% identity with ScyR4.

CLUSTAL multiple sequence alignment by MUSCLE (3.8)

```
QTE75998.1_ScyL2      MATETVTIHQPKDALAKFTYLEWHDHYRTERPFQALDILHNAVDKREGNVSFKEGGKEVV
CasL3                 MATTTIT---SQDAPAEFTYLEWRDHYKTERPFQAIDILEDAIDKRPGNVFFKQGQPEVV
QTE75983.1_ZopL9      MATATVT---TAPTVVRTT----ADYYDAPPVLKIHTYTRESYEEQFGNKSVIH-HPINL
CasL8                 ----------MSLQVINTT----VDYYVPPATHGLHGISDSDFEEQYGDKCVDH-HPIKL
                                        *     *:* .           .  ::. *:  .       :

QTE75998.1_ScyL2      HDVRGHEQDFTLDKHGFLFANAPTSLS-PSDFQDDEKIKEKYLPECETYLKQYFDNVDQV
CasL3                 RDIRGHEKEFTLDGHGFLFADAPTSLS-PADFKDEDKIKSVYLPECKSYLKKYFDNVDQV
QTE75983.1_ZopL9      KDIRA--ANINLQNNGFQLIKLQSKLTNPDDYLDEETVKRVYIPELAEAVKKL-TGATEV
CasL8                 RDMRA--GDFSLEKNGFQLVQHHSK---PIDFSDLEAVKNNYFPGVAETIKKH-TGASEV
                      .*:*.   ::.*: :** : .  :.   * *: * : :*  *:*     :*:   .. :*

QTE75998.1_ScyL2      HIIHYRVRCTNSSDP------NSPTGPAKVVHVDQSGPHVTERIHKAFPDCADFLLRGHV
CasL3                 HIIHHRVRCTDTTDP------NSPTGPARTVHVDQSGPHVEERIRKAFPDDADFLLRGHV
QTE75983.1_ZopL9      RVLNPKVRDSSTEKDGFENNWGKNNGAVRRIHIDLAPGGVEEALYPIFGEEYMKSIAGRW
CasL8                 HVVLPKIRNTSTEKSGFAKGWNNNNGPIRRSHIDVAPGGIVEGLGPVFGEEYLRSIAGRW
                      .::  .:* :.: .       .. .*. .  *:* :   : * :   * :     : *.

QTE75998.1_ScyL2      RLINLWRPINGPIQNWPLAVCDANSLPEENLIETDRIRKAQK-GNTRFVIQA--PSMKWY
CasL3                 RLINLWRPINGPIQNWPLAVCDANSLPEDNLVETDRIRKAQK-GNTRFVVQV--PAMRWY
QTE75983.1_ZopL9      RLINAWKPTR-PVERDPLAVCD--RVPDEDLVPLQRVVPGKALMEQRYHLKTGKKDHDWY
CasL8                 KLVNAWKPLS-LVERDPLAVID--RIPEEDLITLERAIPGKTMKQERYLIKAGQEEHDWY
                      .*:* *.*    ::. **** *   :*:::*:  :*   .:   : *: ::.      **

QTE75998.1_ScyL2      YQSKMEDNTLLVFKSY--ESQDGVAKYASHCSFPLPTAGPMTPPRESIELRAFVFTYPRD
CasL3                 YQSGMEDNTLLVFKSY-DSTQAAVAKYAAHCSFPLPTAGPATPPRESIELRAFVFTYPRD
QTE75983.1_ZopL9      YASNQQPDEVLLFTQYSDFPNRNTADRVPHVSVKLPGQEDK-PRRTSVDARCLVVW----
CasL8                 FAPLQRPDELLLFTQYSDMPNRGPADRVAHASVRLPDTDDK-PPRTSIEVRVLVVW----
                      : .    : :*:*..*   .:   *. ..* *. **      * * *:: * :*.

QTE75998.1_ScyL2      EST
CasL3                 L--
QTE75983.1_ZopL9      ---
CasL8                 ---
```

Figure S13: Multiple sequence alignment using MUSCLE[15] of ScyL2 (QTE75998.1), ZopL9 (QTE75983.1), CasL3 and CasL8. An asterisk (*) marks completely conserved amino acid residues, whereas a colon (:) identifies highly conserved residues and a period (.) indicates moderately conserved residues. CasL3 shows 80.77% identity with ScyL2, and 28.89 % identity with ZopL9. CasL8 shows 27.69% identity with ScyL2, and 56.46 % identity with ZopL9.

15.3. Percentage identity matrices

Table S3: Percentage identity matrix for AsaB-like αKGDDs in the scytalidin, zopfiellin and castaneiolide BGCs, generated using MUSCLE[15]

```
             Percent Identity  Matrix - created by Clustal2.1

        1: QTE75998.1_ScyL2  100.00   80.77   26.77   27.69
        2: CasL3              80.77  100.00   28.89   27.20
        3: QTE75983.1_ZopL9   26.77   28.89  100.00   56.46
        4: CasL8              27.69   27.20   56.46  100.00
```

16. Enzyme expression and purification

16.1. General procedures

pET151 plasmids containing *E. coli* codon optimised and His-tagged genes were transformed into Agilent BL21 gold cells according to the manufacturer's protocol. A single colony was used to inoculate LB media (4 x 800 mL flasks) supplemented with 100 µg/mL ampicillin. At OD600 0.5-0.7 protein expression was induced by the addition of IPTG (100 µL, 1 M stock). The cells were incubated overnight, then collected and resuspended in column buffer A (20 mM Tris pH 7.9, 10 mM imidazole, 500 mM NaCl, 10% glycerol). Sonication was used to disrupt the cells before centrifugation at 15,000 rpm for 30 minutes. The supernatant was loaded onto a Nickel column (GE Healthcare HiTrap 5 mL) and eluted with column buffer A. The target protein was collected by eluting with column buffer B (20 mM Tris-HCl pH 7.9, 500 mM imidazole, 500 mM NaCl, 10% glycerol). The proteins were purified using size exclusion chromatography and eluted into storage buffer (50 mM Tris-HCl pH 8, 20% glycerol). SDS page analysis and/or ESMS confirmed the proteins had the expected mass.

16.2. ZopL9

Conducted as previously.[16]

16.3. ZopL2

The sequence for ZopL2 was codon optimised for *E. coli* and purchased as a synthetic gene from ThermoFisher Scientific. The sequence included an N-terminal 6xHis-tag (>ZopL2 (His-tag/ linker underlined)) and was cloned into the expression vector pET151 to give pET151-ZopL2. This was expressed in *E. coli* BL21 (DE3) cells and the protein was purified using IMAC and SEC chromatography. SDS PAGE (Figure S14, A) showed the presence of ZopL2 (expected mass 23583.16 Da) and further confirmation that the correct protein had been obtained was shown by ESMS (Figure S14, B).

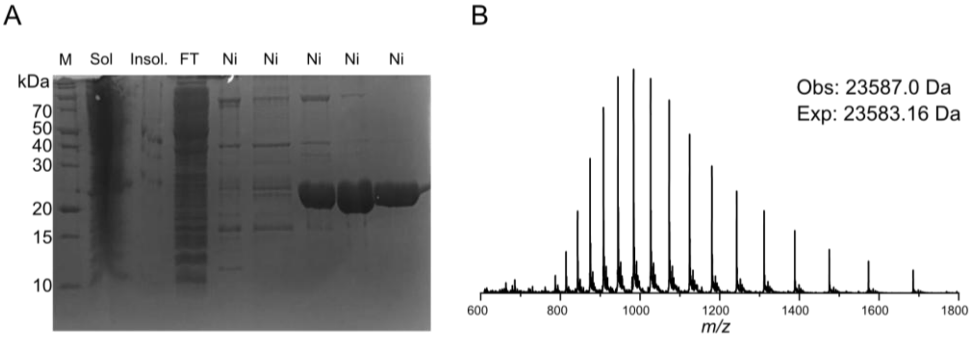

Figure S14:**A)** SDS-PAGE and **B)** MS characterisation of ZopL2. Abbreviations: M = protein marker, Sol. = soluble cell fraction, insol. = insoluble cell fraction, FT = IMAC flow through, Ni = fractions collected from the IMAC column.

16.4. Analytical size exclusion experiment with ZopL9 and ZopL2

Samples of ZopL2, ZopL9 and an equimolar mixture of ZopL2 and ZopL9 were analysed by analytical Size Exclusion Chromatography (SEC). ZopL2 and ZopL9 did not appear to form a higher-order complex, as the elution volume is unchanged (Figure S15).

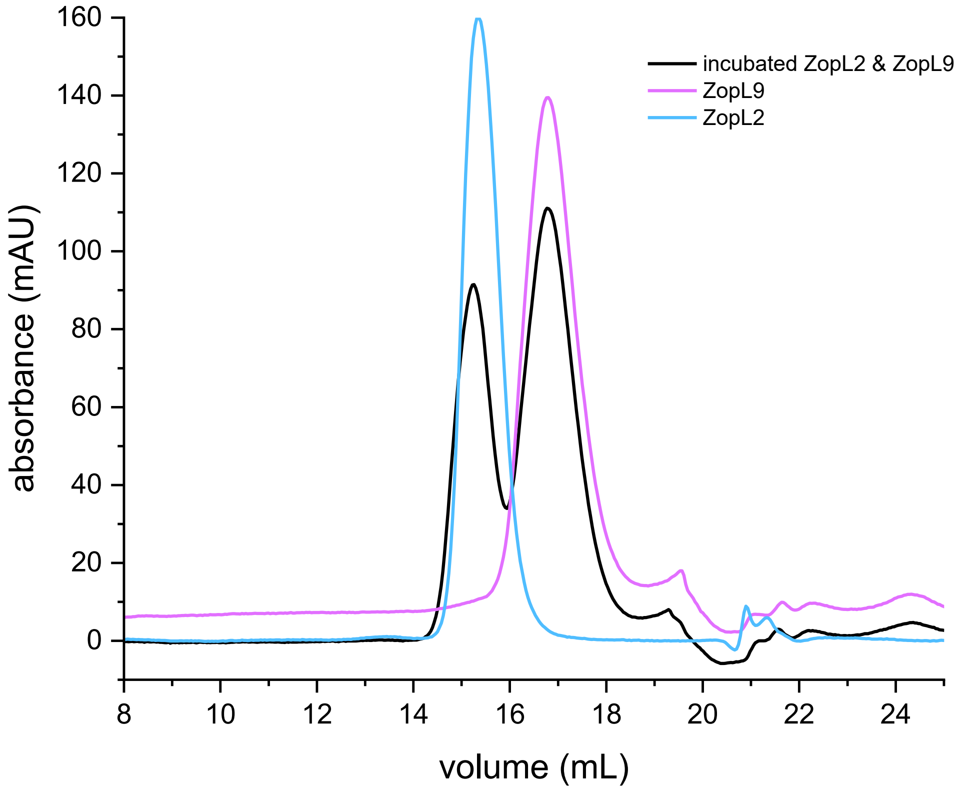

Figure S15: Size Exclusion analysis of purified ZopL9 (pink), ZopL2 (blue), and an equimolar mixture of both proteins (black). The elution volume of the mixture does not shift compared to the individual proteins.

16.5. DcDO1

DcDO1 sequence was codon optimised for *E. coli* and an N-terminal 6xHis-tag added (>DcDO1 (His-tag/ linker underlined)), and purchased from ThermoFisher Scientific in the expression vector pET151. The protein was expressed in *E. coli* BL21 (DE3) and purified by IMAC and SEC. SDS-PAGE (Figure S16) showed the presence of DcDO1 (expected mass 35266.23 Da)

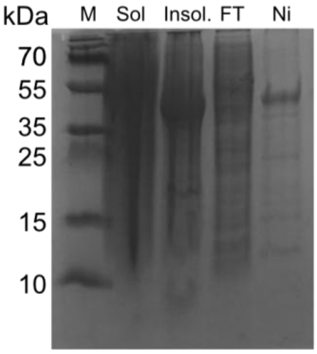

Figure S16: SDS-PAGE analysis of DcDO1. Abbreviations: M = protein marker, Sol. = soluble cell fraction, insol. = insoluble cell fraction, FT = IMAC flow through, Ni = fractions collected from the IMAC column.

17. Enzyme assays

In vitro assays with ZopL9, ZopL2 and DcDO1 were conducted as previously.[16] Assays with a total volume of 200 µL were prepared, containing 50 mM PBS buffer (pH 7.8), 0.1 mM of substrate (deoxyscytalidin **5**), 1.0 mM $(NH_4)_2Fe(SO_4)_2$, 10 mM α-ketoglutarate, 8 mM ascorbic acid and 10 µM enzyme(s). After incubation at 30 °C for 18 hours, 200 µL of MeOH was added to terminate the reaction. Protein was removed by centrifugation and the supernatant was extracted with EtOAc (2 x 400 µL). The extract was dried and dissolved in MeCN prior to LC-MS analysis.

18. LC-MS analysis of enzyme assays

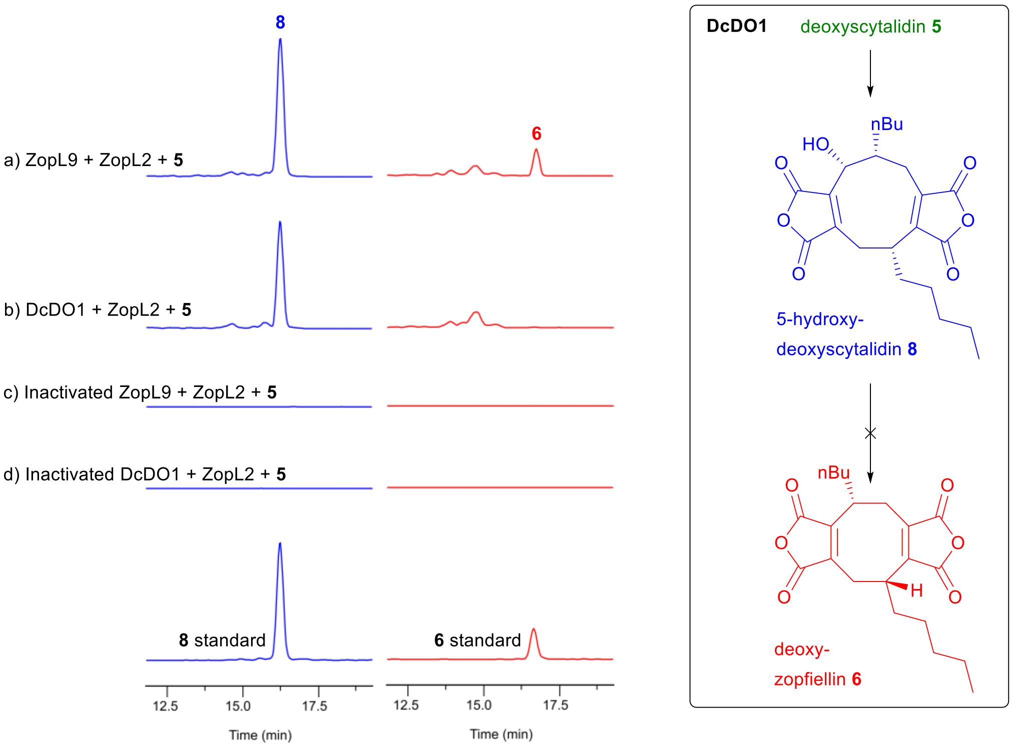

Figure S17:EIC ES^{-} (5-95% MeCN:H_2O gradient) from enzyme assays with ZopL9, ZopL2, DcDO1 and deoxyscytalidin **5**. Traces **(a)** *D. curvata* ZopL9 plus ZopL2 and deoxyscytalidin **5**. Turnover to 5-hydroxy-deoxyscytalidin **8** and deoxyzopfiellin **6** observed. Traces **(b)** *D. curvata* DcDO1 plus ZopL2 and deoxyscytalidin **5**. Turnover to 5-hydroxy-deoxyscytalidin **8**.Traces **(c)** Inactivated *D. curvata* ZopL9 plus ZopL2 and deoxyscytalidin **5**. No turnover observed. Traces **(d)** Inactivated *D. curvata* DcDO1 plus ZopL2 and deoxyscytalidin **5**. No turnover observed.

19. Synthesis

All reagents were sourced from commercial suppliers and were used without further purification unless stated otherwise. All reactions using anhydrous solvents were performed using standard Schlenk syringe-septa techniques, with flame dried glassware under a positive pressure of nitrogen. Anhydrous DCM was dried by passing through a modified Grubbs system of alumina columns, manufactured by Anhydrous Engineering. Flash column chromatography was performed according to the procedures used by Still *et al.*[17] using silica gel (Fisher Scientific or Aldrich) and a suitable eluent.

19.1. Synthesis of alkene 11

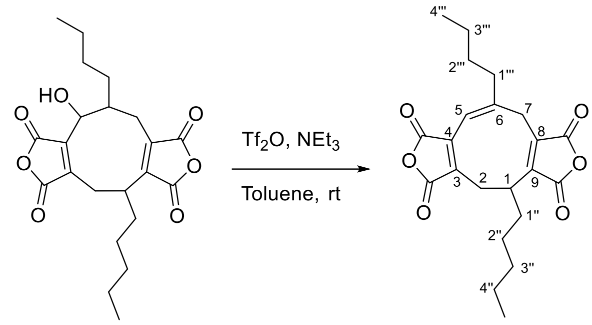

5-hydroxy-deoxyscytalidin **8** alkene **11**

To a solution of alcohol **8** (30.0 mg, 74.2 µmol) in toluene (0.5 mL) was added Tf_2O (0.06 mL, 0.357 mmol) and triethylamine (0.05 mL, 0.359 mmol) dropwise. The reaction was stirred for 4 hours before quenching with water (5 mL) and diluting with DCM (5 mL). The resulting layers were separated and the aqueous phase was further extracted with DCM (2 × 5 mL). The combined organic layers were dried over $MgSO_4$, filtered, and concentrated *in vacuo*. The crude material was purified by column chromatography (5-10% EtOAc in petroleum ether) to give alkene **11** (16.6 mg, 58%) as a yellow solid. $[\alpha]_D^{23}$= +247 (*c* = 1.00, $CHCl_3$); ^1H NMR ($CDCl_3$, 400 MHz) 6.27 (1H, s, 5-H), 3.85 (1H, br. s, 7-*H*H), 3.32 (1H, d, *J* 15.5, 7-H*H*), 3.23 (1H, m, 1-H), 3.06-2.90 (2H, m, 2-H_2), 2.51-2.37 (2H, m, 1‴-H_2), 1.79-1.65 (2H, m, 1″-H_2), 1.64-1.57 (2H, m, 2‴-H_2), 1.45-1.35 (3H, m, 2″-*H*H, 4″-H_2), 1.34-1.27 (5H, m, 2″-H*H*, 3″-H_2, 3‴-H_2), 0.97 (3H, t, *J* 7.5, 5″-H_3), 0.89 (3H, t, *J* 7.0, 4‴-H_3);^{13}C NMR ($CDCl_3$, 125 MHz) 165.7, 165.3, 165.2, 165.1 (C=O × 4), 149.0, 146.5, 144.2, 143.3 (C-3, C-4, C-8, and C-9), 138.4 (C-6), 115.8 (C-5), 43.0 (C-1‴), 36.6 (C-1″), 36.3 (C-1), 31.5 (C-3″), 29.7 (C-2‴), 29.0 (C-7), 28.4 (C-2), 27.0 (C-2″), 22.5 (C-3‴), 22.4 (C-4″), 14.1 (C-5″), 14.0 (C-4‴); HRMS (ESI) calc. for $C_{22}H_{26}O_6Na$ 409.1622 $[M+Na]^+$, found 409.1636; IR (ν_{max}/cm^{-1}) (neat): 2931, 1770.

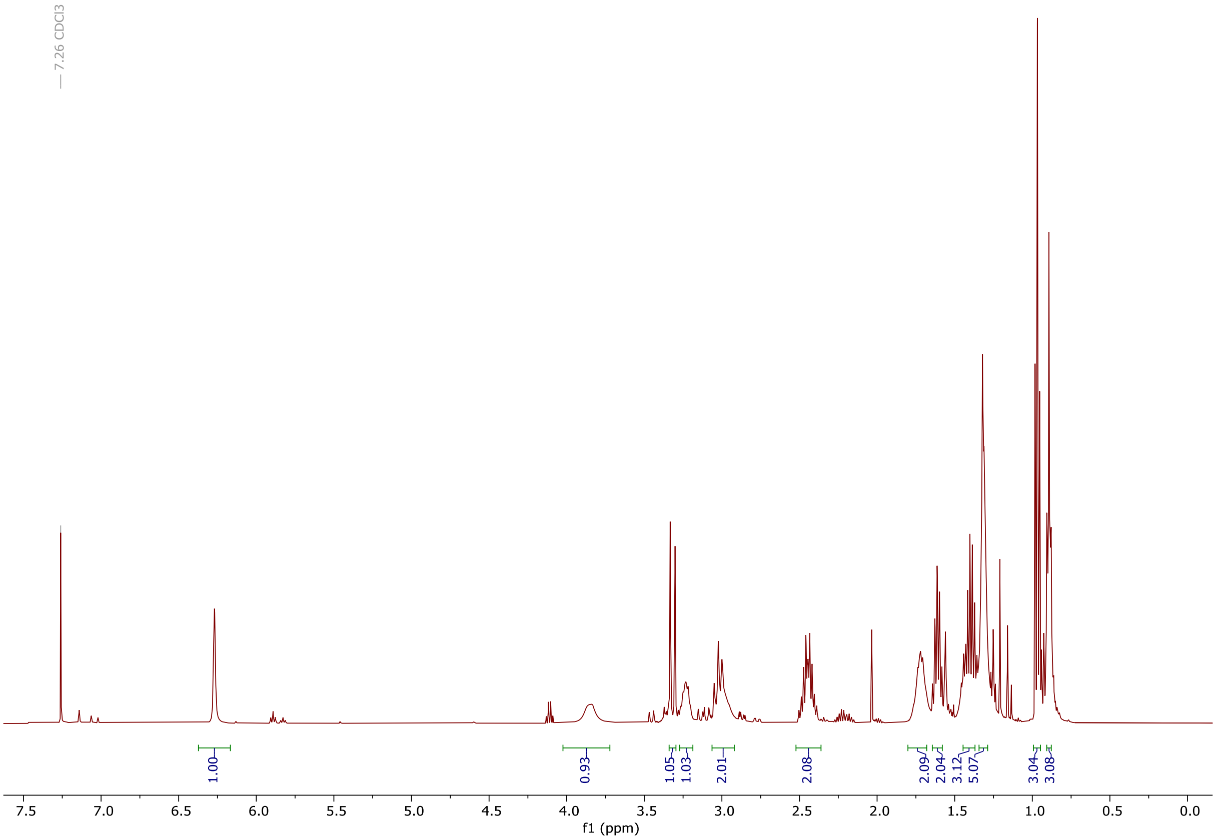

Figure S18: ^{1}H NMR spectrum (400 MHz, $CDCl_3$) of **11**.

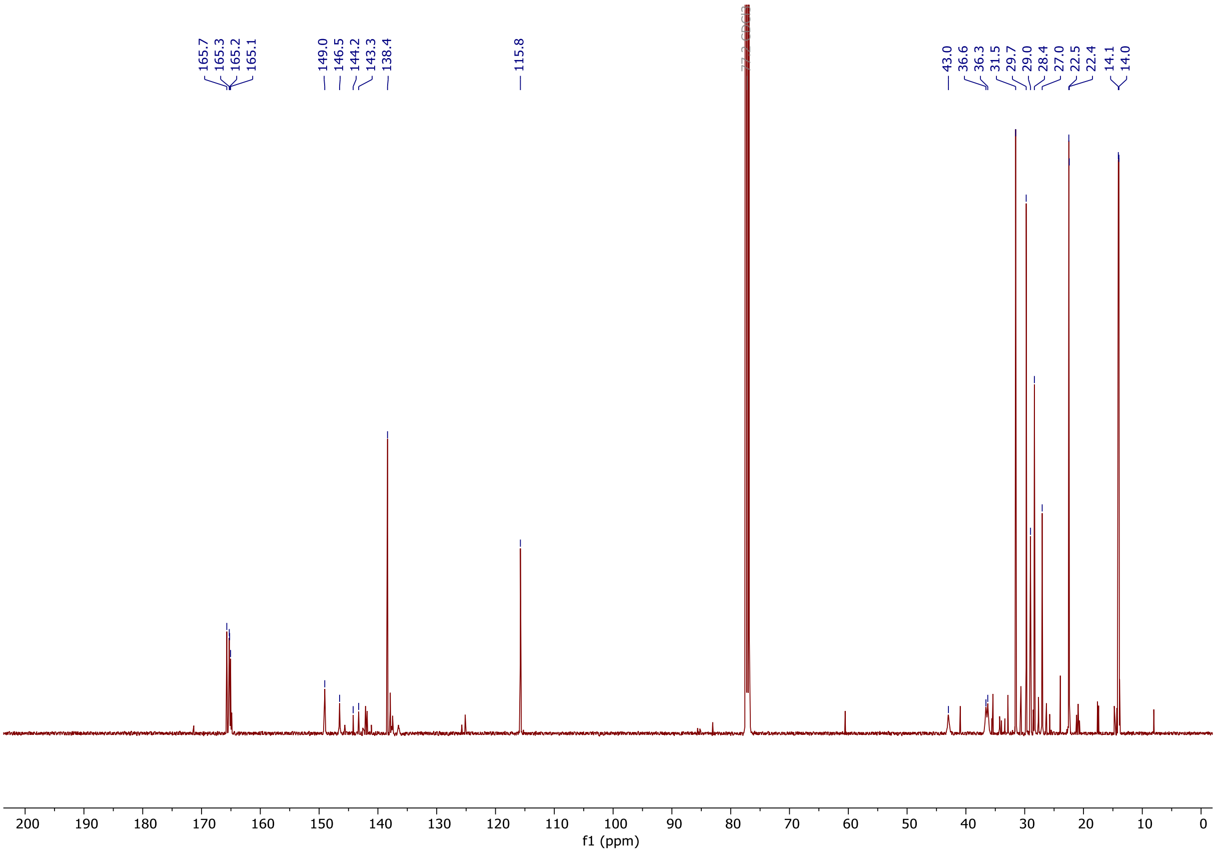

Figure S19: ^{13}C NMR spectrum (125 MHz, $CDCl_3$) of **11**.

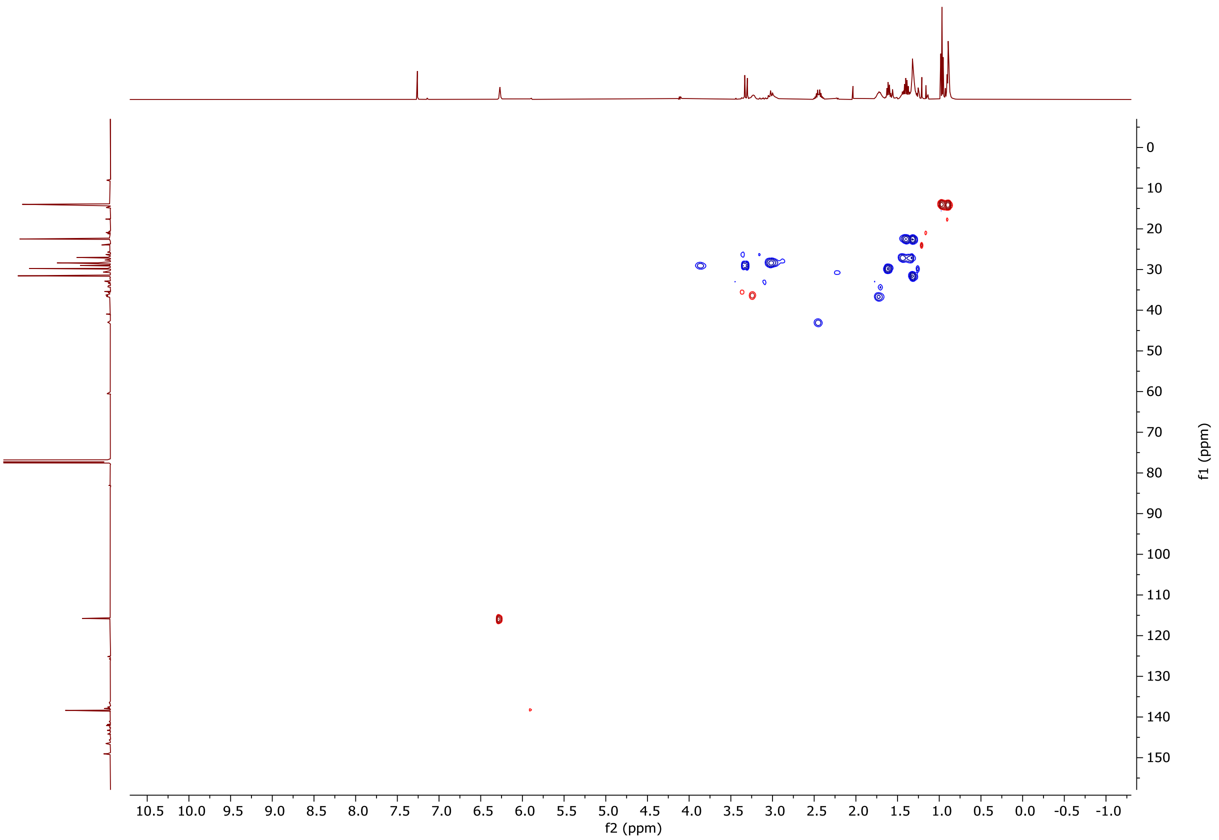

Figure S20: HSQC spectrum (400 MHz, $CDCl_3$) of **11**.

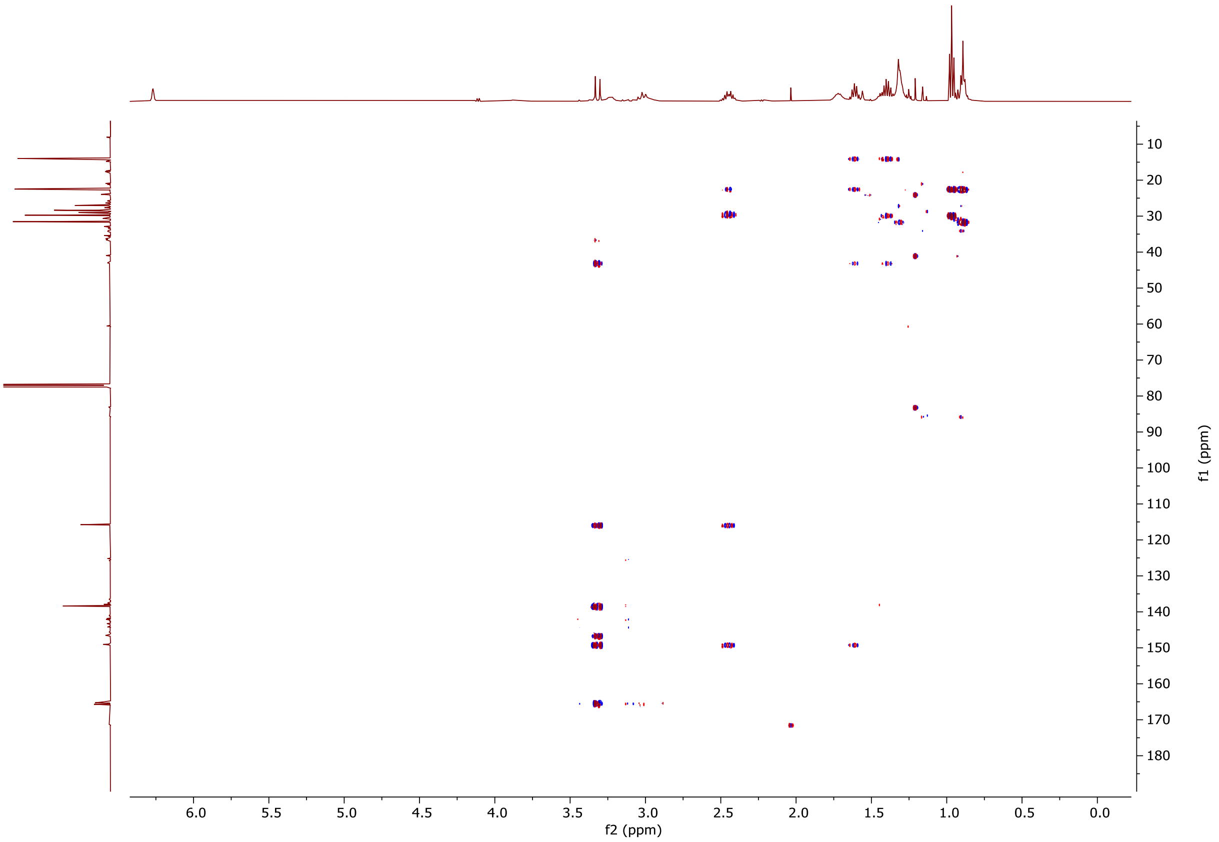

Figure S21: HMBC spectrum (400 MHz, $CDCl_3$) of **11**.

20. Isolated metabolites

20.1. Dihydro-5-hydroxy-deoxyscytalidin 9

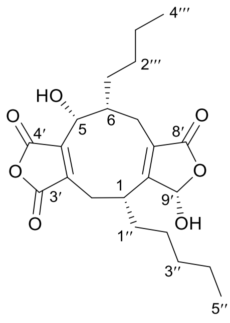

dihydro-5-hydroxy-deoxyscytalidin **9**

The working transformant from *D. curvata* DC-L9 was cultured for seven days in PDB at 25 °C. The starter culture was used to inoculate sterilised media containing PDB (96 g) dissolved in water (4 L). The flasks were shaken at 180 rpm at 25 °C for four weeks. The broths were acidified with 1M HCl and extracted with ethyl acetate (3 x 4 L). The organic layers were concentrated under reduced pressure to give crude extracts (970 mg and 880 mg, respectively). The crude extracts were subjected to column chromatography over Sephadex LH-20 (MeOH) and further purified by preparative LC-MS (Luna 5µ C18 100 Å, 250 x 10 mm, 8 mL/min) using a 20 min 10-95% acetonitrile in water (+ 0.1% formic acid) programme to afford compound **9** (from DC-L9, tR = 11.0 min, 35.0 mg) as a yellowish solid. $[\alpha]_D^{25}$ – 78.2° (*c* 0.3, $CHCl_3$); UV/vis (DAD) λ_{max} nm: 252; IR (KBr) ν_{max} 3446, 2929, 2859, 1846 (C=O), 1760 (C=O), 1459, 1256 cm^{-1}; ^1H NMR(500 MHz, $CDCl_3$) δ 5.72 (1H, s, 9′-H), 4.78 (1H, s, 5-H), 3.50 (1H, m, 2-*H*H), 2.97 (1H, m, 1-H), 2.94 (1H, m, 2-H*H*), 2.88 (1H, m, 7-*H*H), 2.08 (1H, d, *J* 14.0, 7-H*H*), 1.77 (1H, m, 6-H), 1.65 (2H, m, 1″-H_2), 1.55 (2H, m, 1‴-H_2), 1.48 (2H, m, 2‴-H_2), 1.38 (2H, m, 2″-H_2), 1.34 (2H, m, 3‴-H_2), 1.30 (2H, m, 4″-H_2), 1.24 (2H, m, 3″-H_2), 0.93 (3H, t, *J* 7.5, 4‴-H_3), 0.88 (3H, t, *J* 7.0, 5″-H_3);^{13}C NMR (125 MHz, $CDCl_3$) δ 171.6 (C-8′), 164.8 (C-4′), 164.5 (C-3′), 159.0 (C-9), 145.1 (C-4), 144.4 (C-3), 130.0 (C-8), 96.6 (C-9′), 67.0 (C-5), 41.4 (C-6), 36.6 (C-1), 36.0 (C-1″), 34.5 (C-1‴), 31.6 (C-3″), 29.2 (C-2‴), 28.3 (C-2), 27.3 (C-2″), 22.6 (C-3‴), 22.49 (C-4″), 22.46 (C-7), 13.98 (C-4‴), 13.97 (C-5″); HRESIMS *m/z* 429.1892 $[M + Na]^+$ (calcd for $C_{22}H_{30}O_7Na$, 429.1889).

The α configuration of these hydroxyl groups established by the singlet coupling constant between vicinal protons H-5 and H-6, which revealed their perpendicular direction, and NOE correlations between H-2a/H-5, H-2a/H-9′ and H-1/H-9′, in the ^1H NMR and 2D NOESY spectra (Table S4 and Figure S23). Finally, the absolute configuration of **9** was determined from the negative sign of its specific optical rotation $[\alpha]_D^{25}$ – 78.2° (*c* 0.3, $CHCl_3$), the same as in the case of deoxyscytalidin **5**[16] and (-)-byssochlamic acid,[18] and from the above evidence the structure of compound **9** was therefore assigned as shown.

Table S4: NMR assignments for dihydro-5-hyroxy-deoxyscytalidin **9** ([a]125MHz, [b]500 MHz in $CDCl_3$)

Position	**Dihydro-5-hydroxy-deoxyscytalidin 9**		
	**δ_C[a]**	**δ_H[b], mult. (*J* in Hz)**	**HMBC**
**1**	36.8	2.97, m	2, 3, 8, 9, 13, 1′
**2**	28.4	3.50, m<br>2.94, m	3, 10, 1′
**3**	144.4	-	1,4, 10
**4**	145.1	-	
**5**	67.0	4.78, s	
**6**	41.4	1.77, m	4, 6, 7, 4′, 1′′′
**7**	22.46	2.88, m<br>2.08, d (14.0)	
**8**	130.0	-	9
**9**	159.0	-	
**3′**	164.5	-	
**4′**	164.8	-	
**8′**	171.6	-	
**9′**	96.6	5.72, s	
**1″**	36.0	1.65, m	
**2″**	27.3	1.38, m	1, 8, 9, 8′
**3″**	31.6	1.24, m	1, 2, 9, 3′′
**4″**	22.49	1.30, m	
**5″**	13.97	0.88, t (7.0)	
**1‴**	34.5	1.55, m	2′′
**2‴**	29.2	1.48, m	2′′, 3′′, 5′′
**3‴**	22.6	1.34, m	3′′, 4′′
**4‴**	13.98	0.93, t (7.5)	

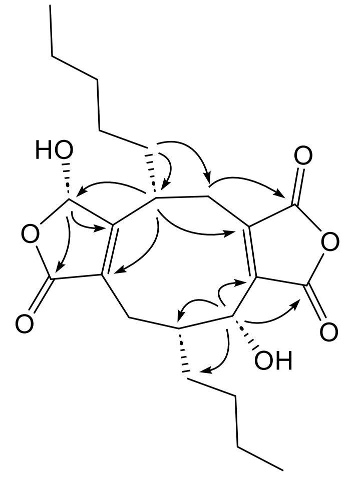

dihydro-5-hydroxy-deoxyscytalidin **9**

Figure S22: Key HMBC correlations of **9**.

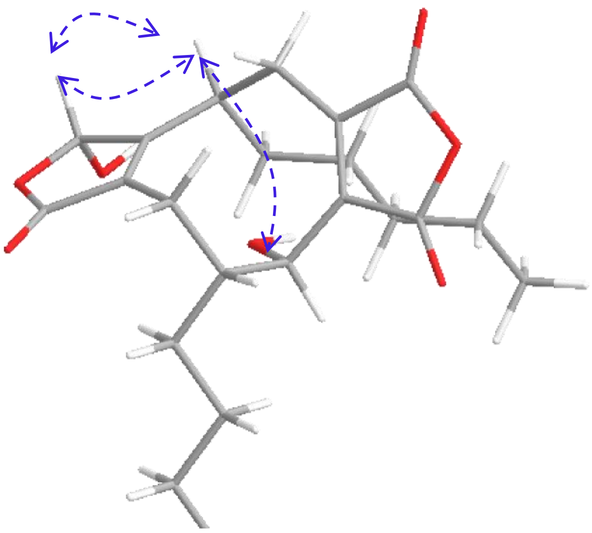

9

Figure S23: Key NOESY correlations of **9**.

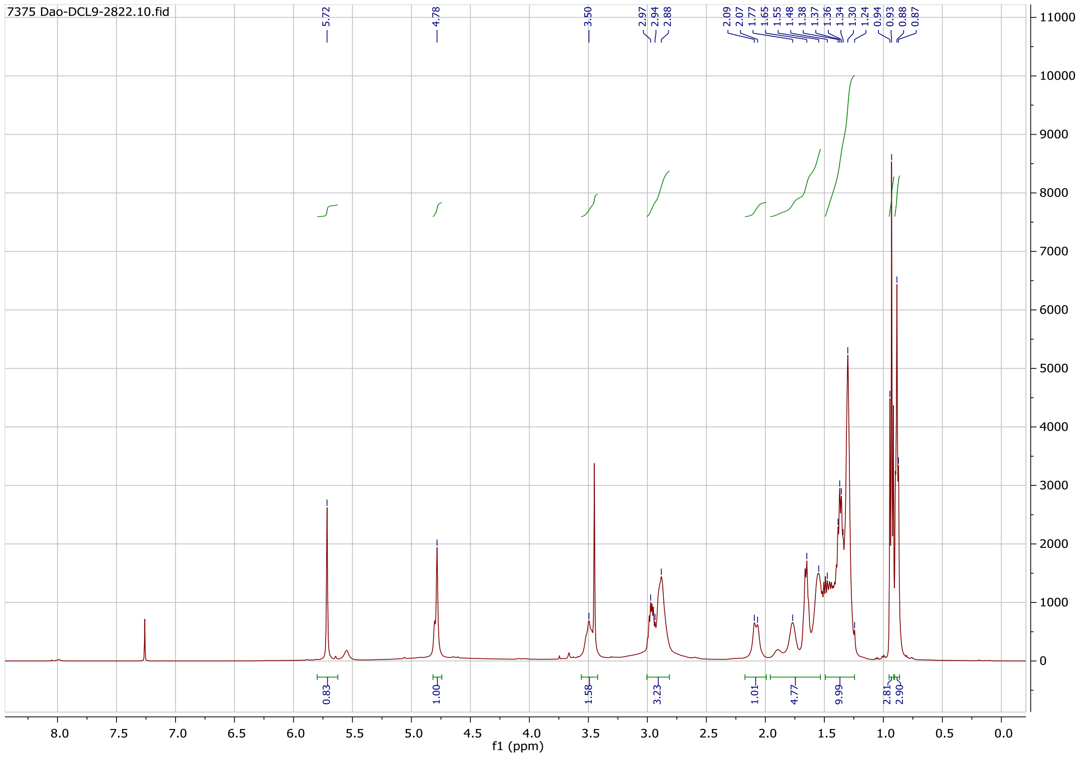

Figure S24: ^{1}H NMR spectrum (500 MHz, $CDCl_3$) of **9**.

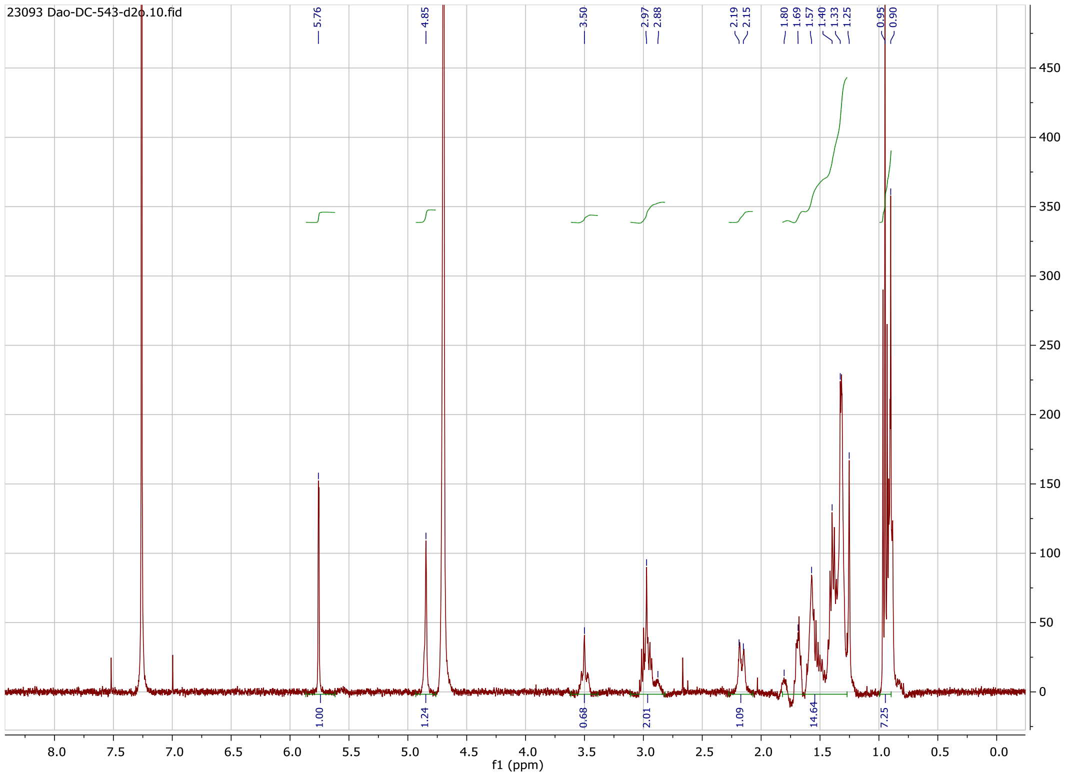

Figure S25: ^{1}H NMR spectrum (500 MHz, $CDCl_3$, drop D_2O) of **9**.

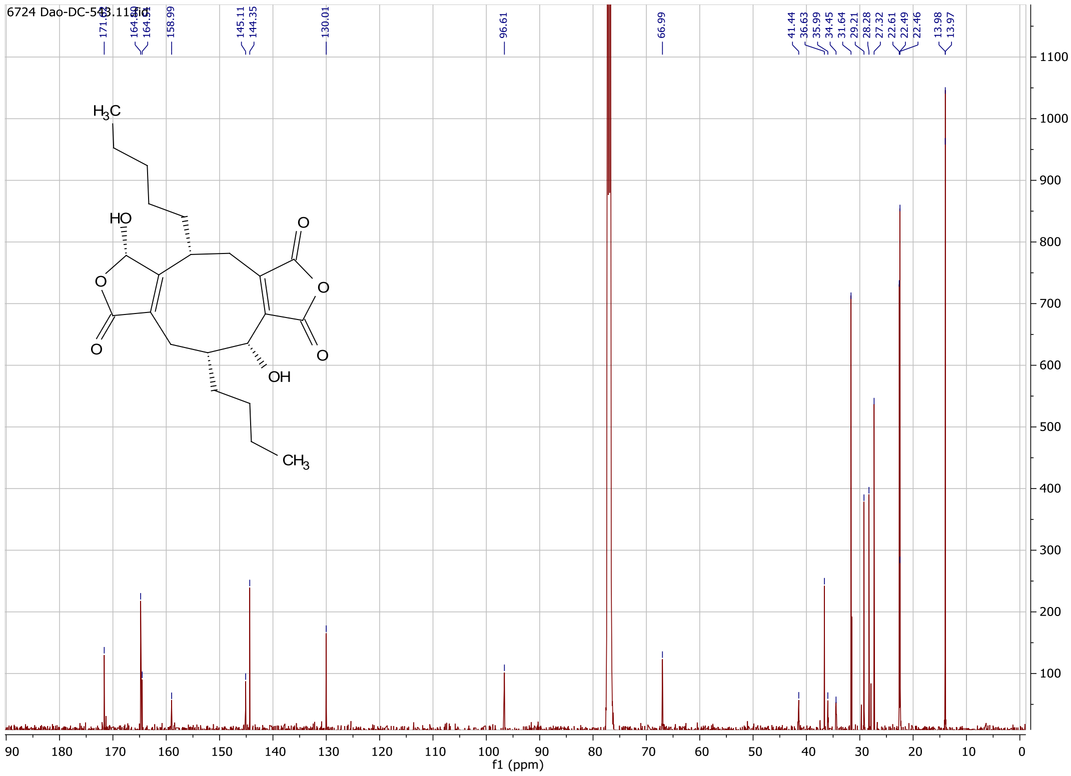

Figure S26: ^{13}C NMR spectrum (125 MHz, $CDCl_3$) of **9**.

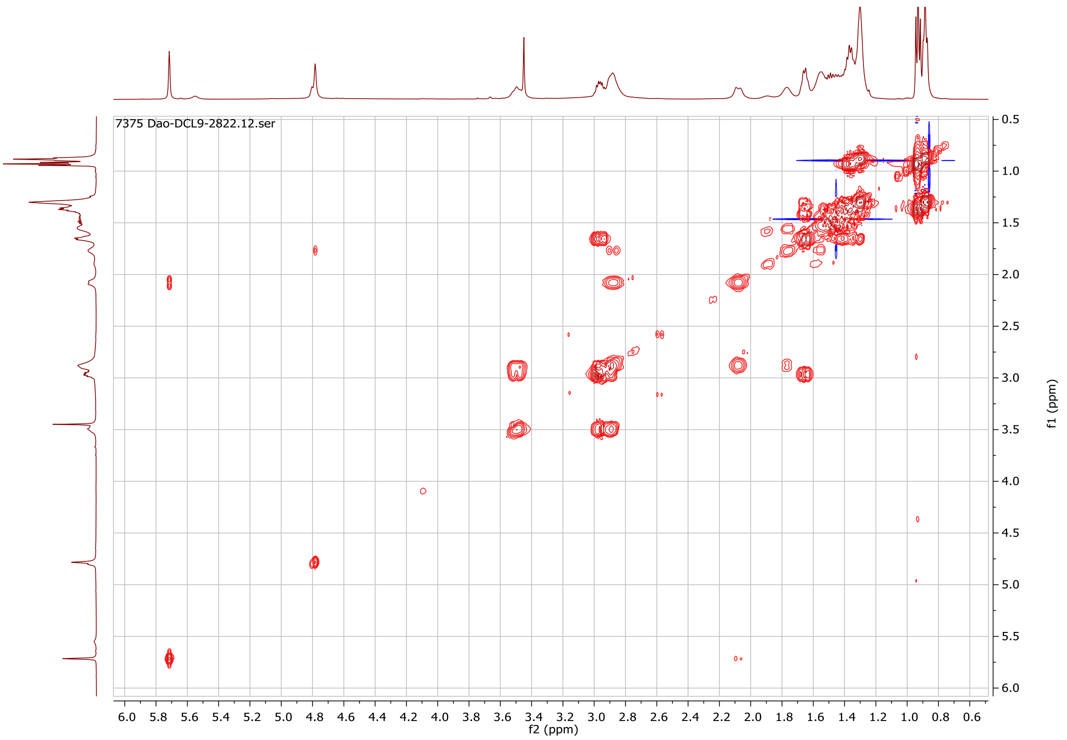

Figure S27: COSY spectrum of **9**.

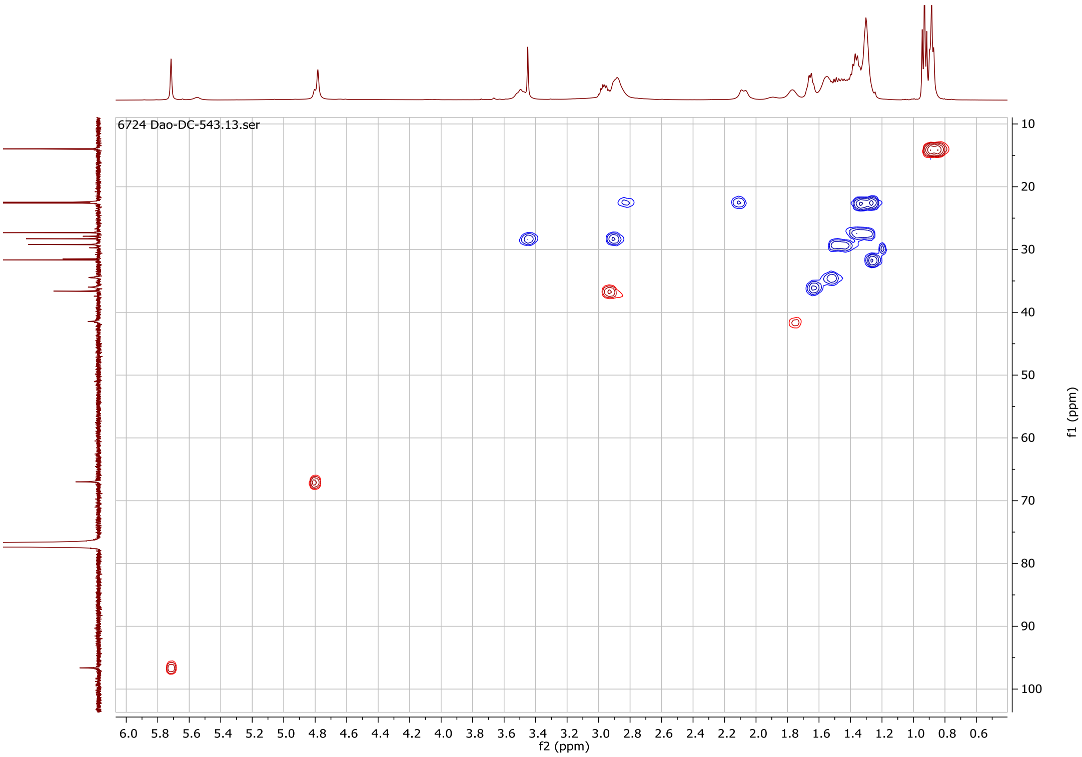

Figure S28: HSQC spectrum of **9**.

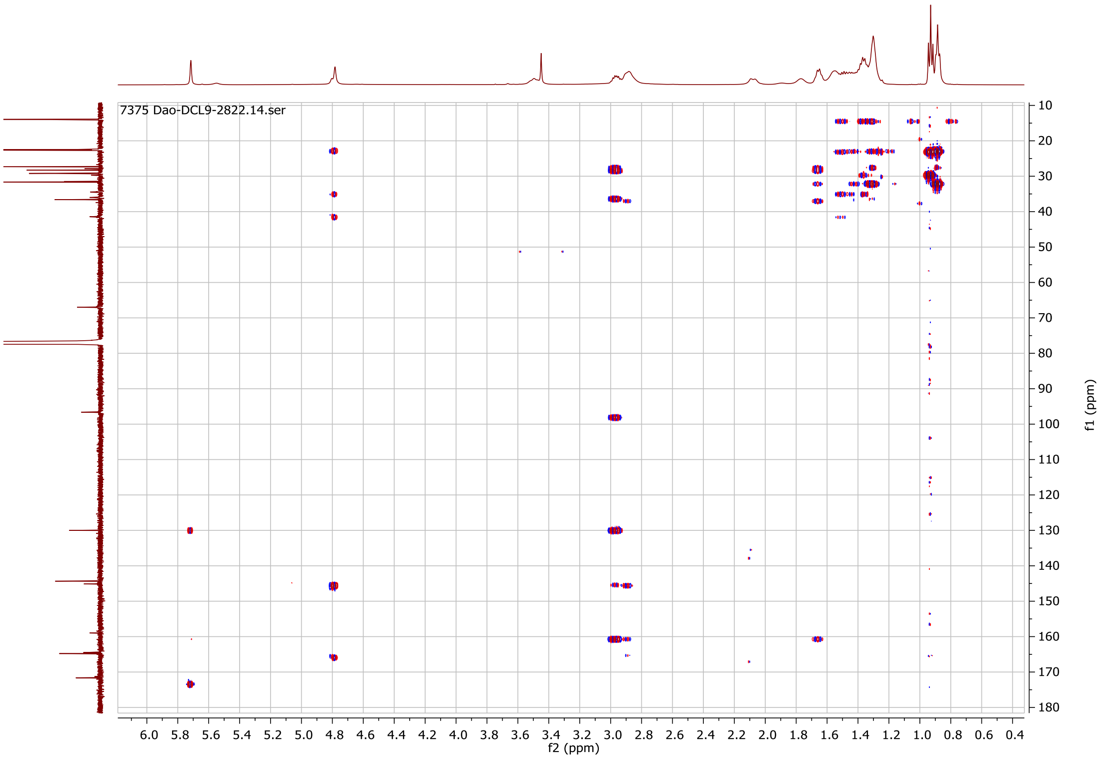

Figure S29: HMBC spectrum of **9**.

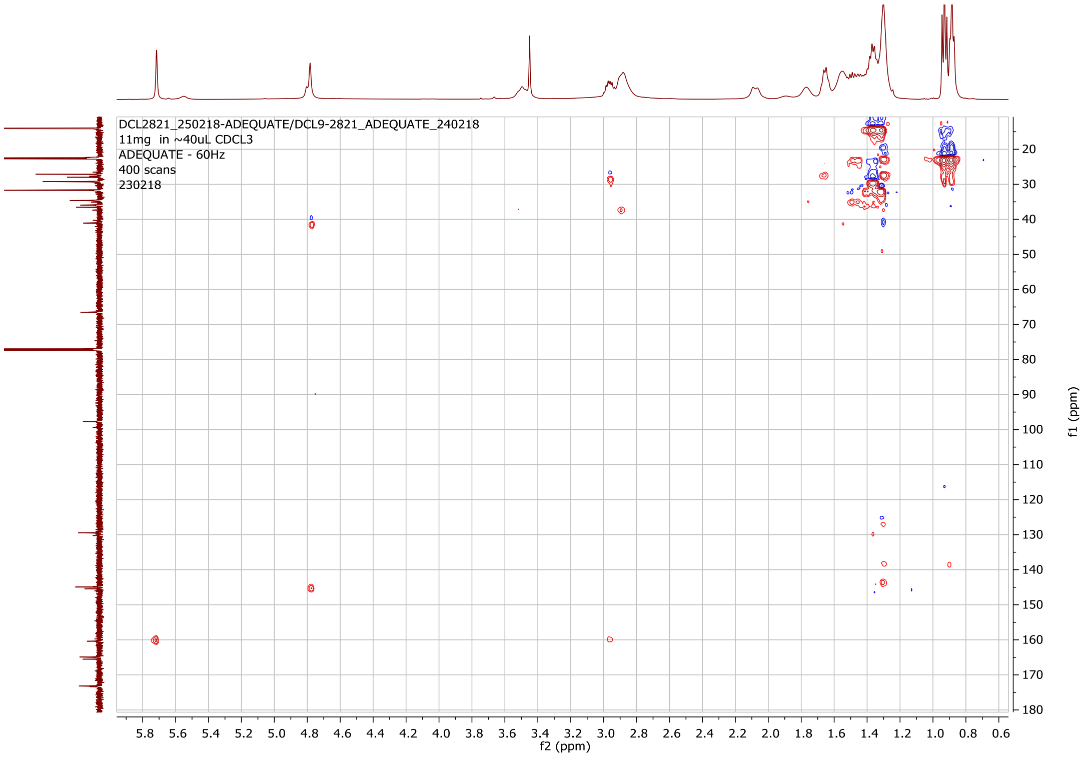

Figure S30: ADEQUATE spectrum of **9**.

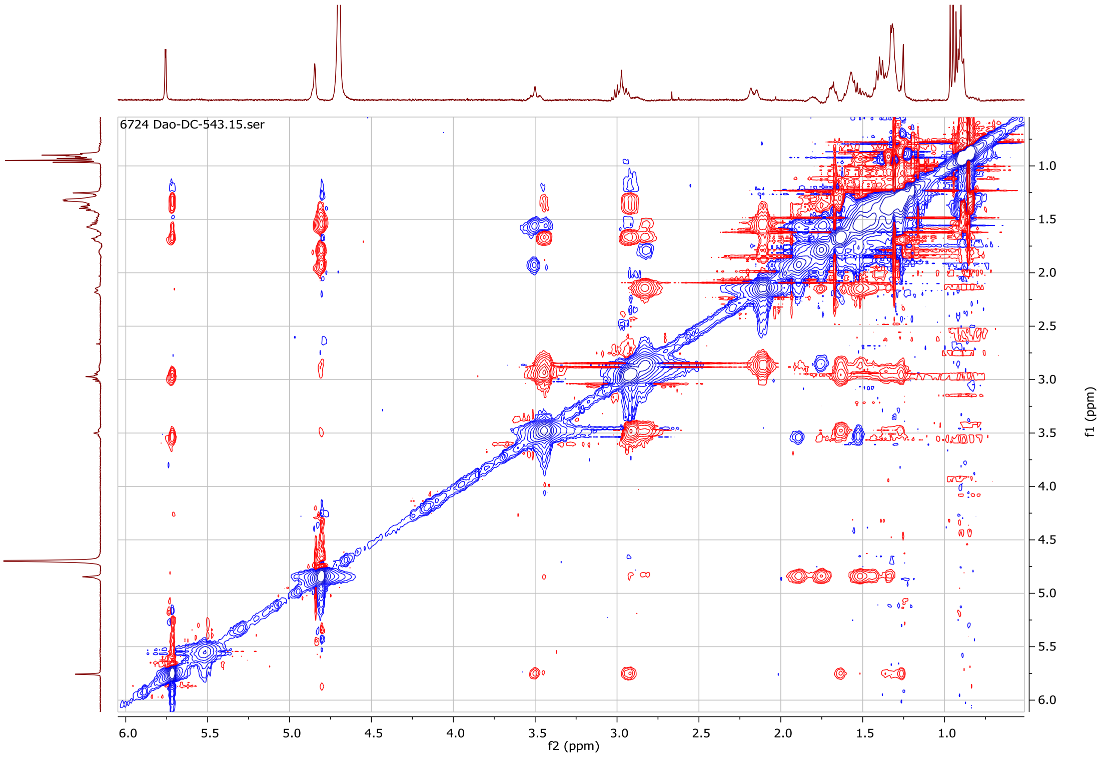

Figure S31: NOESY spectrum of **9**.

20.2. Castaneiolide 10

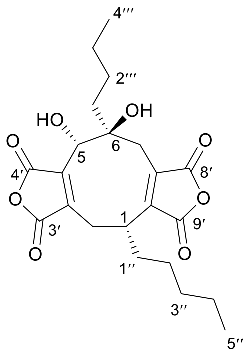

castaneiolide **10**

An agar plug of *M. castaneicola* was used to inoculate a 250 mL non-baffled flask containing PDB (2 x 50 mL) and incubated at 28 °C, 200 rpm shaking for 6 days. A fermentation culture in a 2.5 L baffled flask containing MEB (500 mL) was inoculated with homogenised seed culture (100 mL) and incubated at 28 °C, 200 rpm shaking for 24 days. The culture was acidified to ~ pH 3 with 2 M HCl and then homogenised with an equal volume of ethyl acetate. The homogenised culture was then filtered under vacuum and the filtrate extracted with ethyl acetate. The aqueous layer was washed a further two times with ethyl acetate. The organic extracts were combined, dried over anhydrous magnesium sulphate, filtered and concentrated under vacuum to yield the crude extract as a dark red oil (159.3 mg). The crude material was purified by flash column chromatography (DCM loading, 20-40% ethyl acetate in petroleum ether, 1% acetic acid) to afford castaneiolide **10** as a yellow solid (32.6 mg). R_f = 0.32; α_D^{24} -28.6 (*c* 0.35, EtOH); ^{1}H NMR (600 MHz, $(CD_3)_2CO$) δ 5.40 (1H, s, 5-OH), 4.72 (1H, s, 5-H), 3.76 (1H, s, 6-OH), 3.71 (1H, app. t, *J* 13.2, 2-*H*H), 3.50 (1H, d, *J* 14.4, 7-*H*H), 3.37 – 3.31 (1H, m, 1-H), 3.01 (1H, dd, *J* 13.4, 5.6, 2-H*H*), 2.69 (1H, d, *J* 14.4, 7-H*H*), 1.89 (2H, t, *J* 8.3, 1'''-H_2), 1.81 – 1.72 (2H, m, 1''-

H_2), 1.70 – 1.63 (1H, m, 2'''-*H*H), 1.63 – 1.57 (1H, m, 2'''-H*H*), 1.55 – 1.47 (1H, m, 2''-*H*H), 1.48 – 1.43 (1H, m, 2''-H*H*), 1.41 – 1.37 (2H, m, 3'''-H_2), 1.36 – 1.33 (4H, m, 3''-H_2 and 4''-H_2), 0.95 (3H, t, *J* 7.4, 4'''-H_3), 0.89 (3H, app. t, *J* 7.0 5''-H_3); ^{13}C NMR (313 K, 151 MHz, $(CD_3)_2CO$) δ 167.9 (C-8'), 167.3 (C-9'), 166.3 (C-3'), 165.9 (C-4'), 147.0 (C-3/4), 145.4 (C-9), 144.7 (C-3/4), 142.9 (C-8), 78.5 (C-6), 70.1 (C-5), 42.5 (C-1'''), 36.4 (C-1''), 35.6 (C-1), 32.3 (C-3''), 28.9 (C-7), 27.7 (C-2), 27.2 (C-2''), 25.6 (C-2'''), 23.8 (C-3'''), 23.1 (C-4''), 14.4 (C-4'''), 14.3 (C-5''); HRMS (Nanospray) calculated for $C_{22}H_{28}O_8$ 443.1682 $[M+Na]^+$, found 443.1670.

Table S5: Comparison of NMR data for castaneiolide **10** isolated from *M. castaneicola* against castaneiolide **10** isolated from feeding **7** to *D. curvata* Δ*zopPKS* values in $(CD_3)_2CO$, [a]313 K, 151 MHz, [b]600 MHz. [a),b)] signals can be interchanged.

Position	Castaneiolide 10 (isolated from *M. castaneicola*)			Castaneiolide 10 (isolated from feeding of 7 to *D. curvata* Δ*zopPKS*)	
	δ_C[a]	δ_H[b], mult. (*J* in Hz)	HMBC	δ_C	δ_H, mult. (*J* in Hz)
**1**	35.6	3.31-3.37, m	2, 8, 9, 9′, 1″	35.7	3.36, m
**2**	27.7	3.01, dd (13.4, 5.6)<br>3.71, app. t (13.2)	1, 3, 4, 3′	27.7	3.02, dd (13.5, 5.5)<br>3.71, t (13.5)
**3**[a)]	144.7	-	-	144.9	-
**4**[a)]	147.0	-	-	147.0	-
**5**	70.1	4.72, s<br>5.40 (OH)	3, 4, 6, 7, 4′, 1‴	70.1	4.72, s<br>5.40 (OH)
**6**	78.5	3.76 (OH)	-	78.5	
**7**	28.9	2.69, d (14.4)<br>3.50, d (14.4)	5, 6, 8, 9, 8′, 1‴	29.0	2.70, d (14.6)<br>3.52, d (14.6)
**8**	142.9	-	-	142.8	-
**9**	145.4	-	-	145.4	-
**3′**	166.3	-	-	166.3	-
**4′**	165.9	-	-	166.0	-
**8′**	167.9	-	-	168.0	-
**9′**	167.3	-	-	167.4	-
**1″**	36.4	1.72-1.81, m	1, 2, 9, 3″	36.5	1.78, m
**2″**	27.2	1.43-1.48, m<br>1.47-1.55, m	3″, 4″	27.3	1.30-1.56, m
**3″**	32.3	1.33-1.36, m	1″, 2″, 4″	32.4	1.30-1.56, m
**4″**	23.1	1.33-1.36, m	2″, 3″	23.2	1.30-1.56, m
**5″**	14.3	0.89, app. t (7.0)	3″, 4″	14.4	0.91, t (8.2)
**1‴**	42.5	1.89, t (8.3)	5, 6, 7, 2‴, 3‴	42.6	1.90, t (8.2)
**2‴**	25.6	1.57-1.63, m<br>1.63-1.70, m	6, 1‴, 3‴, 4‴	25.7	1.64, m
**3‴**	23.8	1.37-1.41, m	1‴, 2‴, 4‴	23.9	1.41, m
**4‴**	14.4	0.95, t (7.4)	2‴, 3‴	14.5	0.97, t (7.3)

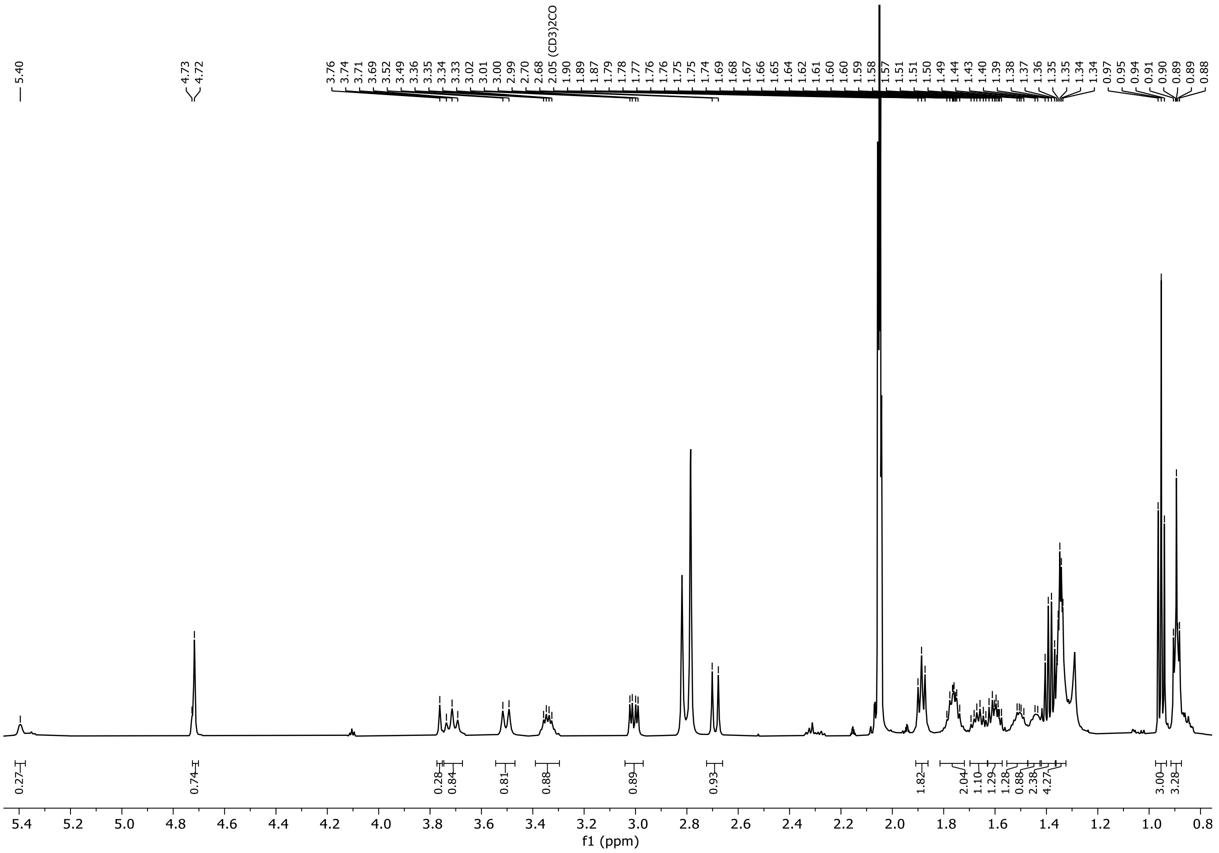

Figure S32: ^{1}H NMR spectrum (600 MHz, $(CD_3)_2CO$) of **10.**

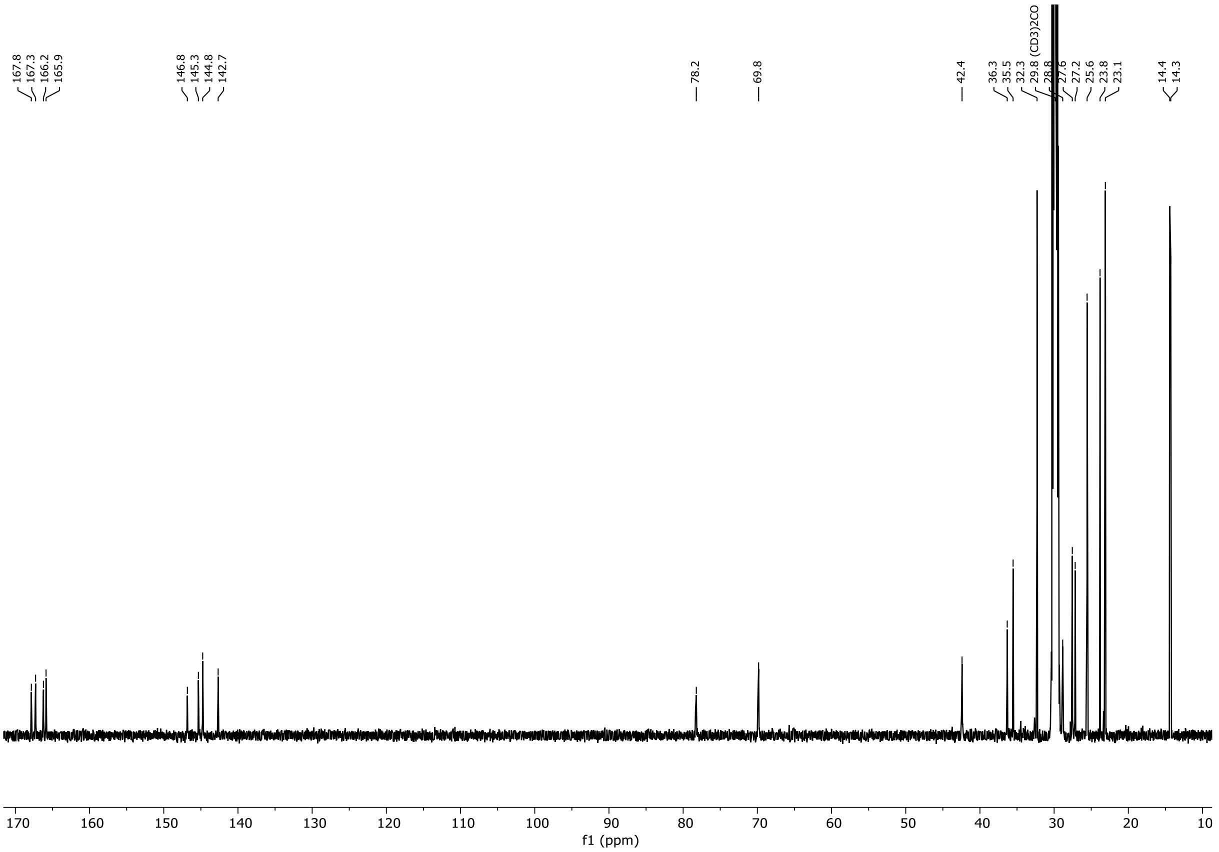

Figure S33: ^{13}C NMR spectrum (313 K, 151 MHz, $(CD_3)_2CO$) of **10.**

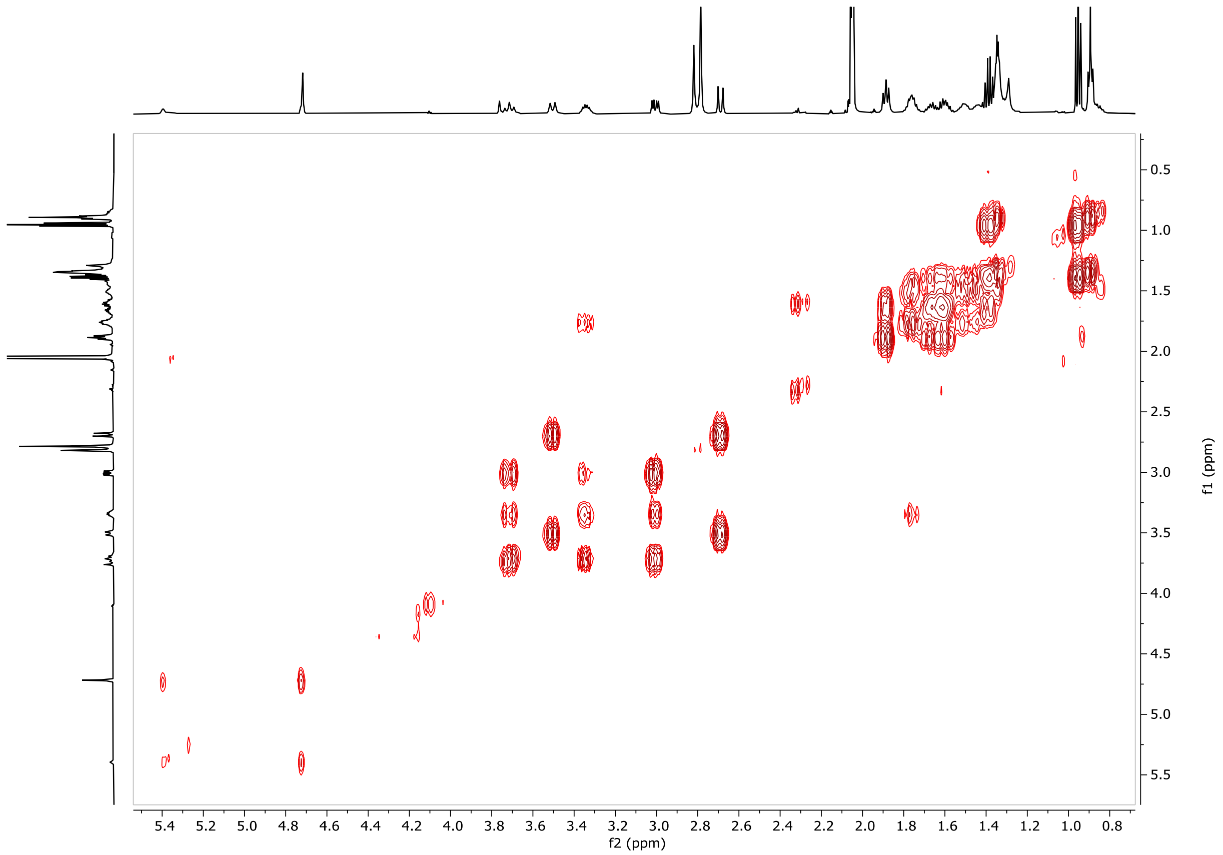

Figure S34: COSY spectrum of **10**.

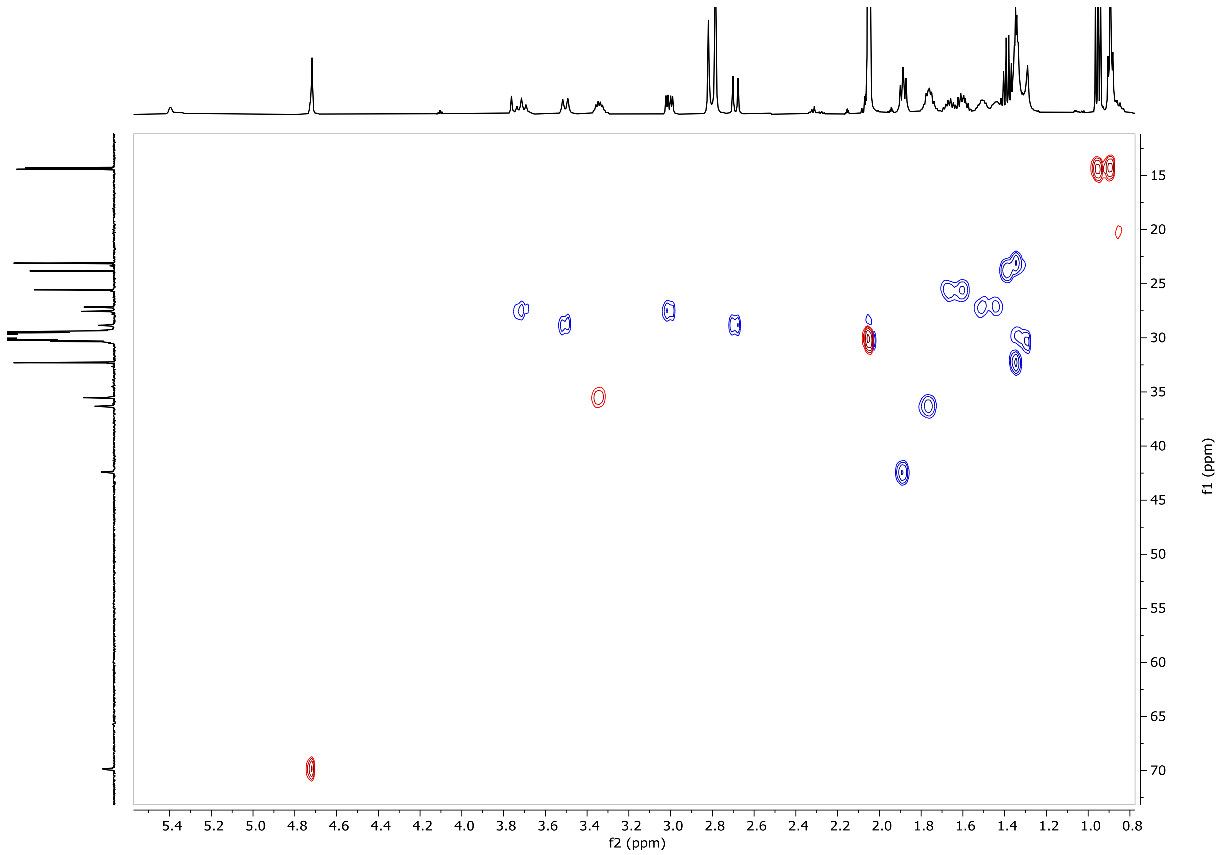

Figure S35: HSQC spectrum of **10**.

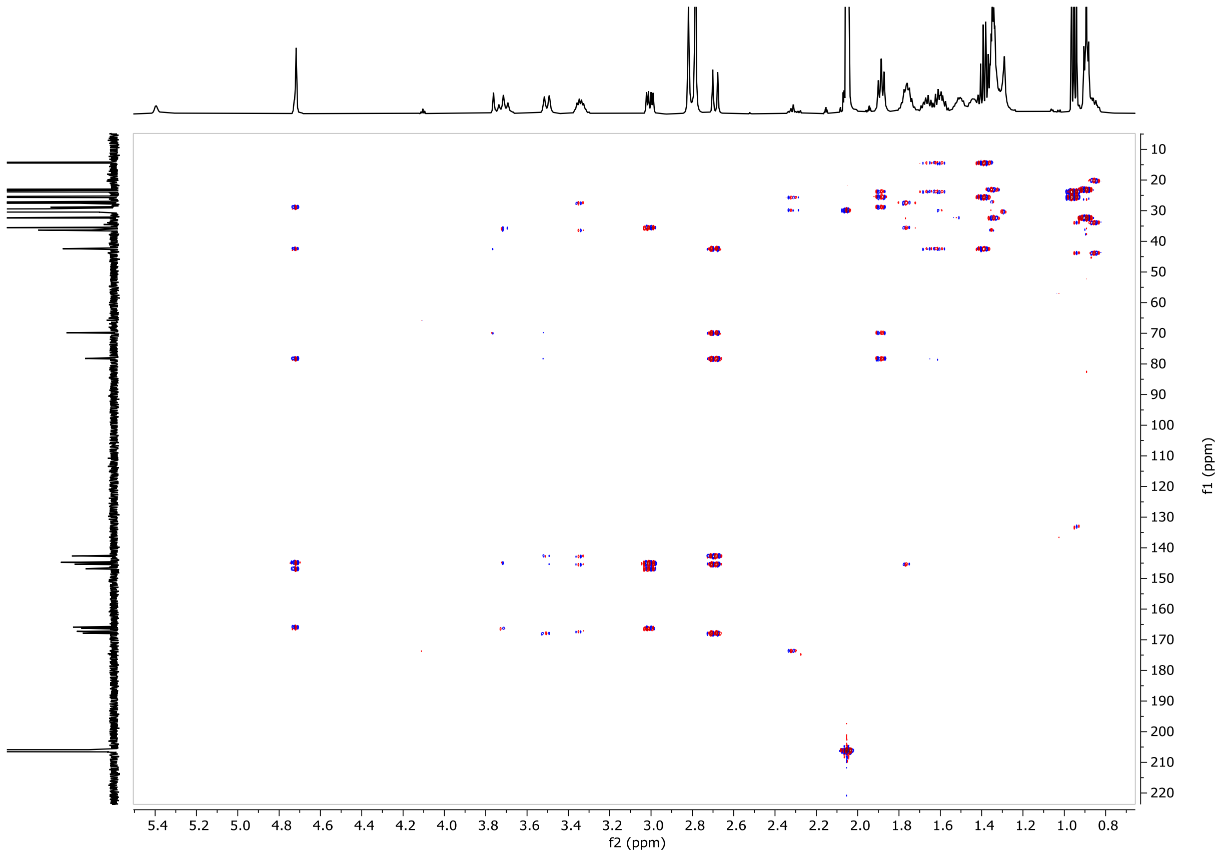

Figure S36: HMBC spectrum of **10**.

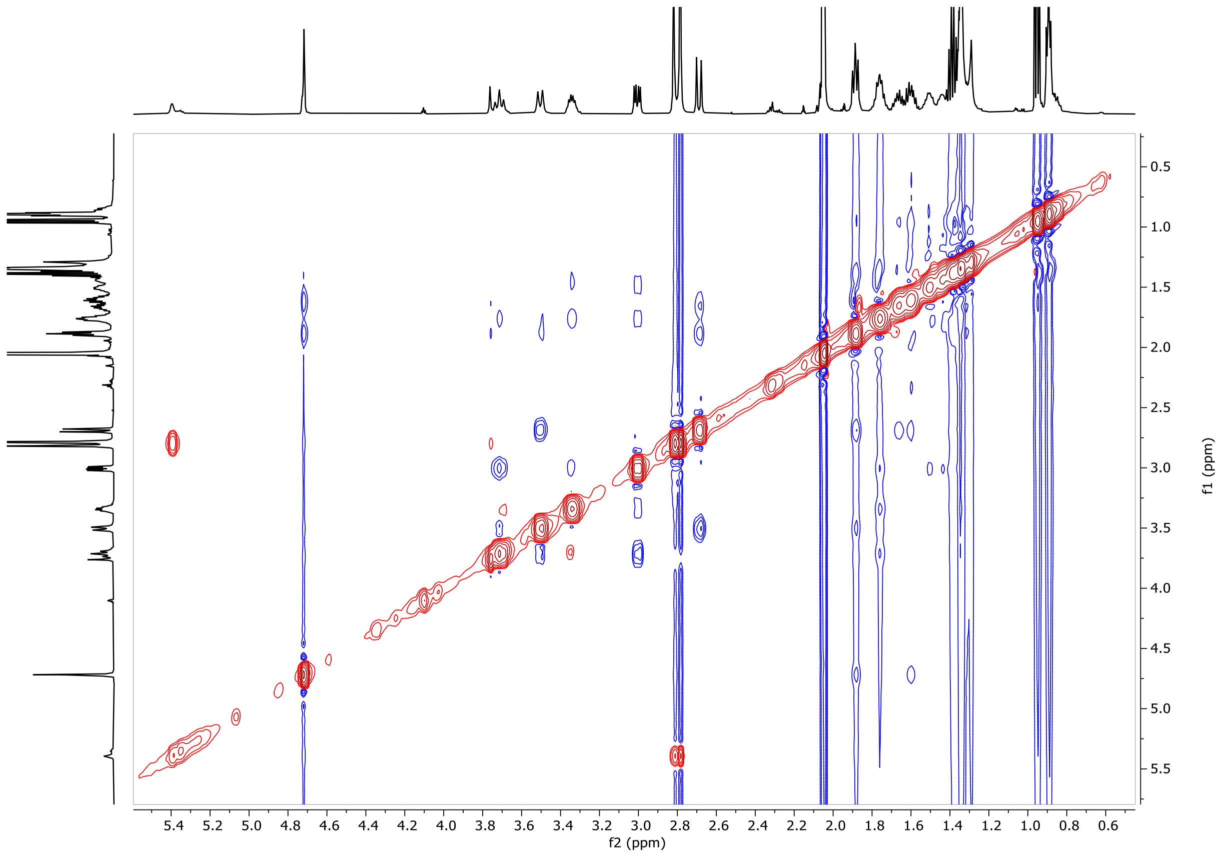

Figure S37: NOESY spectrum of **10**.

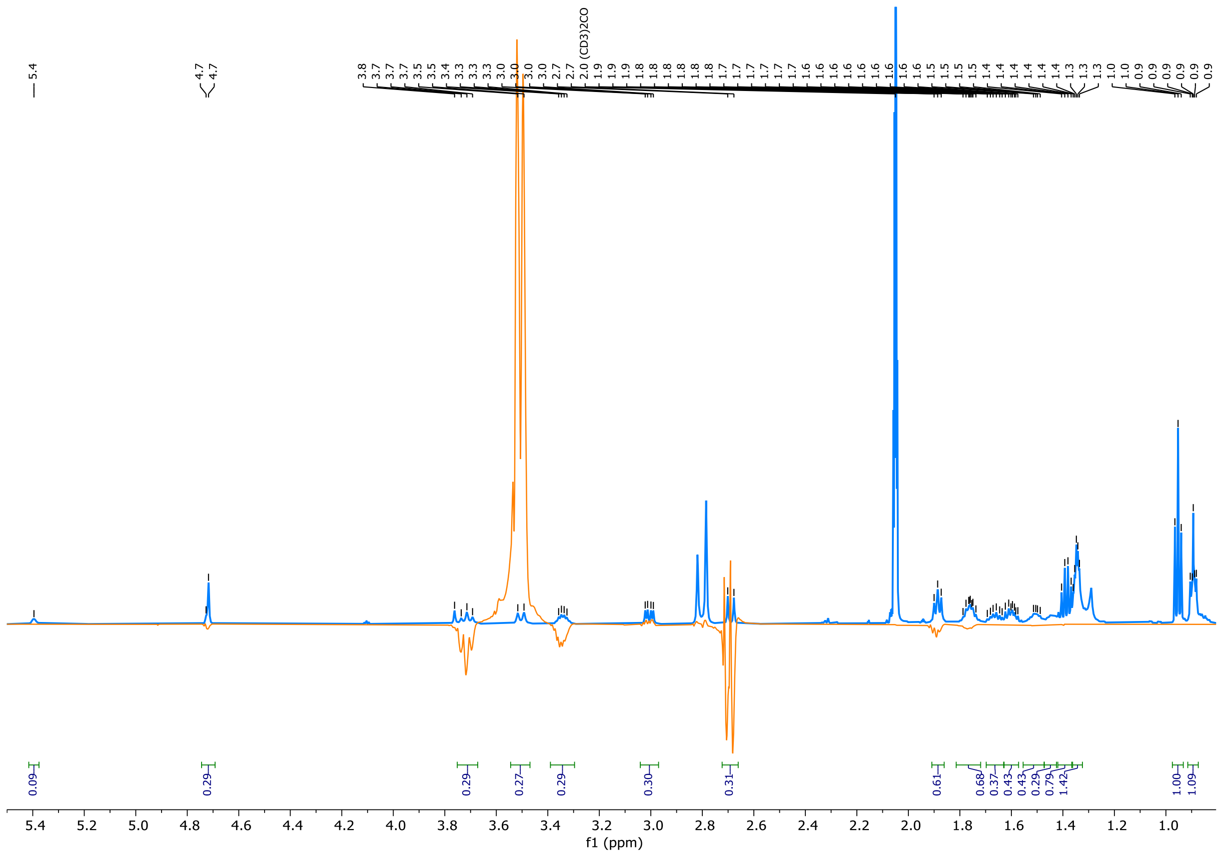

Figure S38: Irradiated NOESY spectrum of **10**.

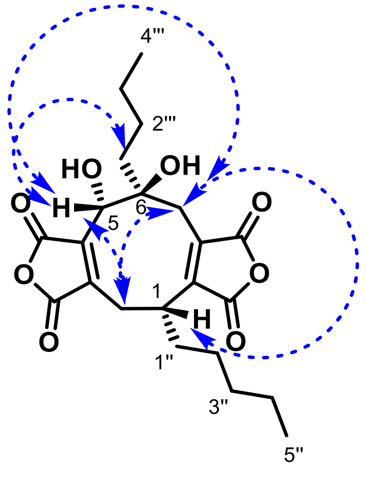

castaneiolide 10

Figure S39: Key NOESY correlations of **10**.

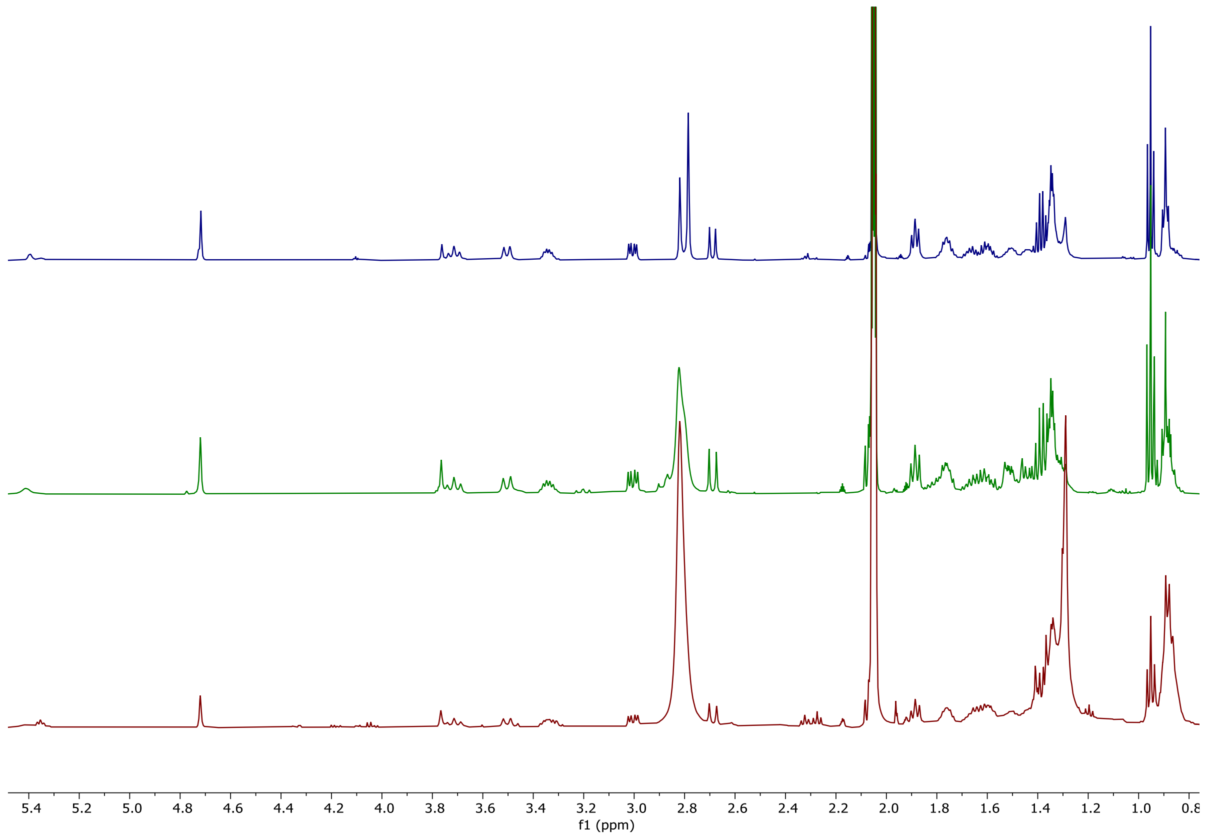

Figure S40: Stacked ^{1}H NMR spectra of **10** isolated from either *M. castaneicola* (blue), scytalidin **7** fed to *D. curvata* Δ*PKS* (green) or alkene **11** fed to *D. curvata* Δ*PKS* (red).

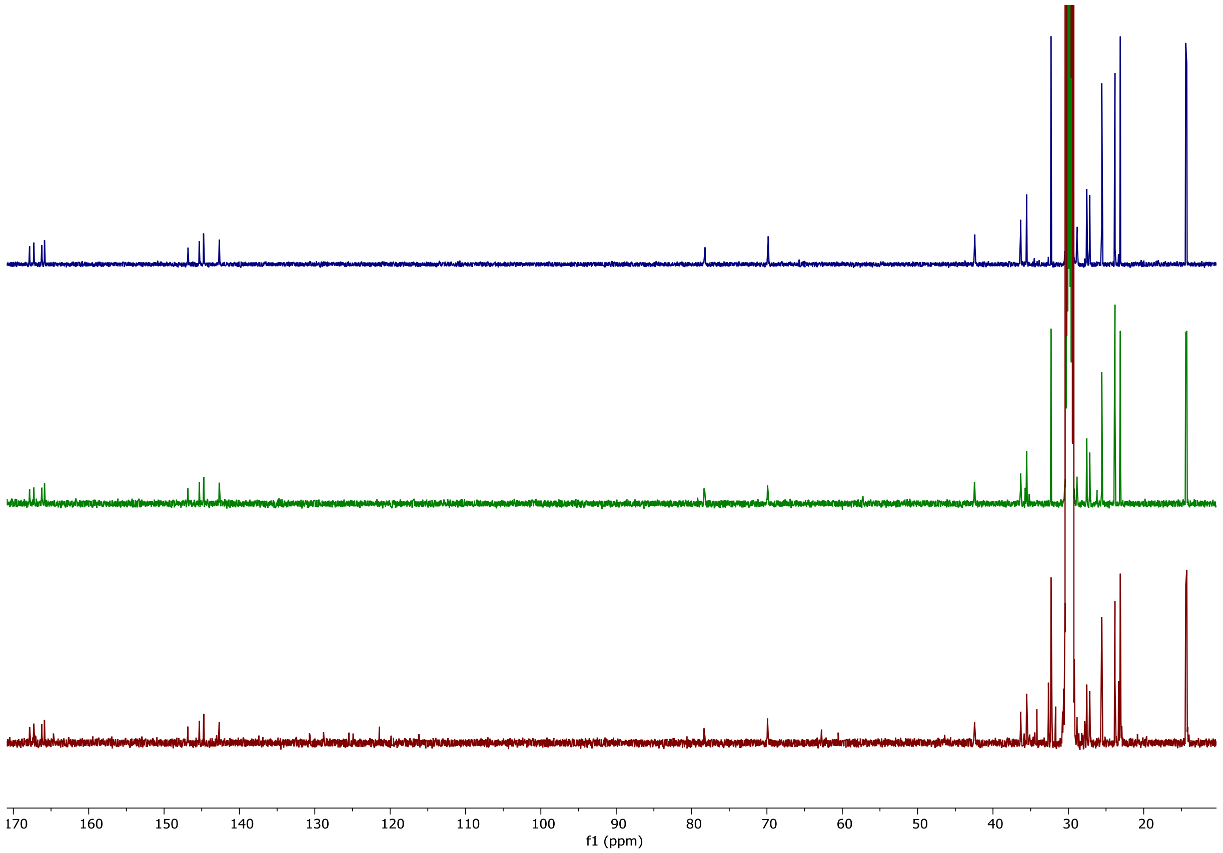

Figure S41: Stacked ^{13}C NMR spectra of **10** isolated from either *M. castaneicola* (blue), scytalidin **7** fed to *D. curvata* Δ*PKS* (green) or alkene **11** fed to *D. curvata* Δ*PKS* (red).

20.3. Impure castaneiolide 10 from feeding alkene 11 to *D. curvata* Δ*zopPKS*

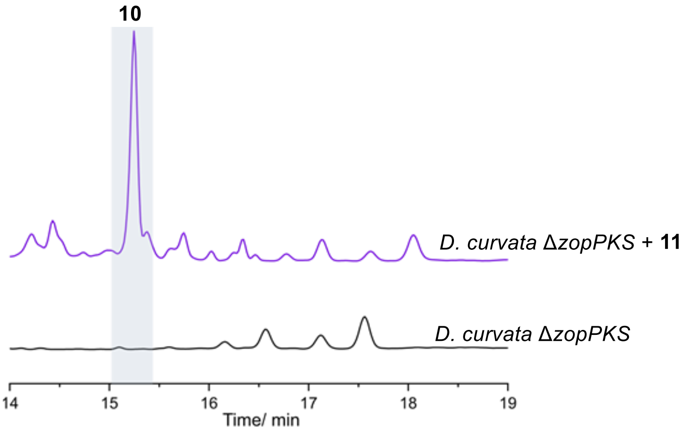

Figure S42: Diode array traces (5-95% MeCN:H_2O gradient) for *D. curvata* Δ*zopPKS* and the Δ*zopPKS* strain fed with alkene **11**, which showed the formation of castaneiolide **10**.

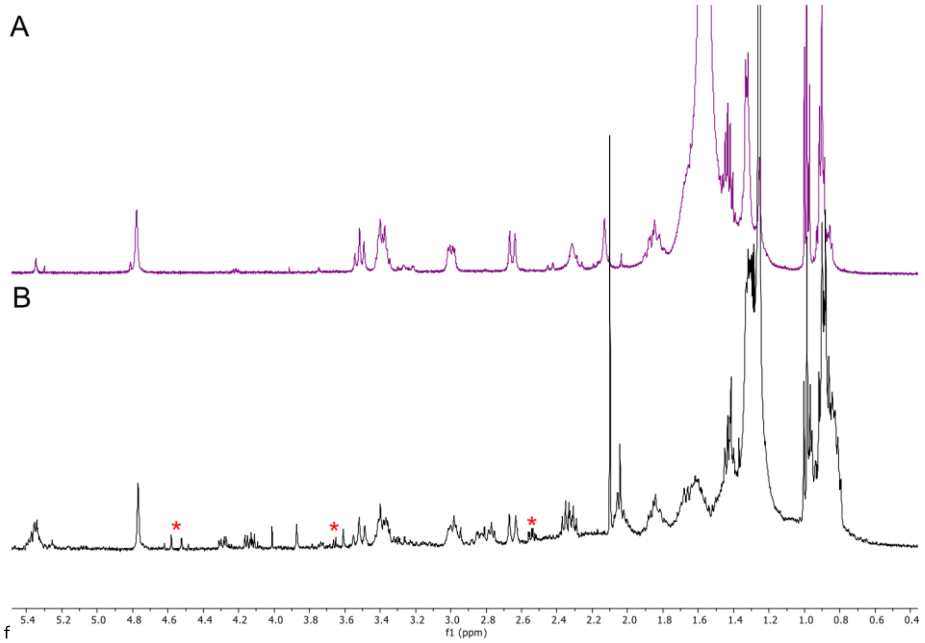

Figure S43: Comparison of the ^1H spectra (400 MHz, $CDCl_3$) for castaneiolide **10** obtained from feeding scytalidin **7** to Δ*zopPKS* (A) and from feeding alkene **11** to Δ*zopPKS*. A clean spectrum could not be obtained for castaneiolide **10** isolated from the alkene feed and several characteristic signals (indicated by asterisks) of the ring open alcohol we which had previously isolated[16] can be seen in the spectra (B).

20.4. Dihydro-zopfiellin **13**

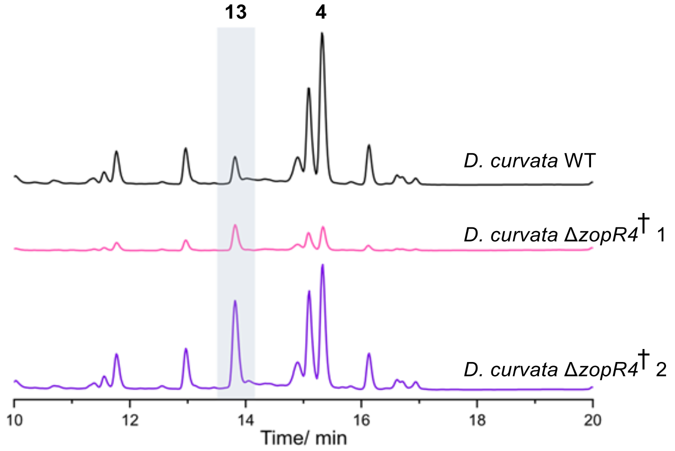

Figure S44: Diode array chromatograms (5-95% MeCN:H_2O gradient) for WT *D. curvata* and the genetically impure Δ*zopR4*† strains, showing the same major products in all traces.

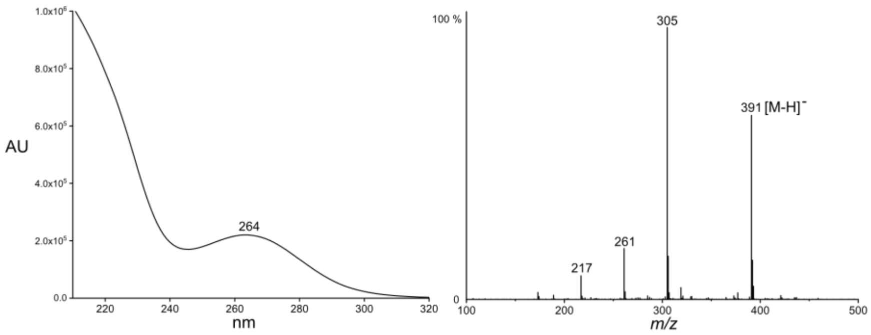

Figure S45: UV and ES⁻ spectra of dihydro-zopfiellin **13**.

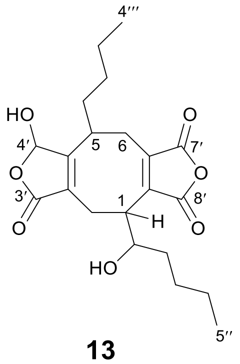

13

Dihydro-zopfiellin **13** was purified from crude extract of the *D. curvata* Δ*zop_R4* strain grown on PDA using preparative HPLC (method 2, table 34). UV λ_{max} = 264 nm; δ_H (500 MHz, $CDCl_3$) 5.91 (1H, s, 4'-H), 3.92 (1H, t, *J* 6.3, 1''-H), 3.17 (1H, *app*. t, *J* 12.2, 6-*H*H), 2.99 - 3. 06 (2H, m, 2-H*H*, 1-H), 2.83- 2.92 (2H, m, 6-*H*H, 5-H), 2.67 (1H, d, *J* 9.1, 2-H*H*), 1.57 – 1.69 (4H, m, 2''-H_2, 2'''-H_2), 1.30 – 1.45 (8H, m, 3''-H_2, 4''-H_2, 1'''-H_2, 3'''-H_2), 0.95 (3H, m, 5''-H_3), 0.93 (3H, m, 4'''-H_3); δ_C (125 MHz, $CDCl_3$) 172.7(C-3'), 166.3 (C-8'), 165.7 (C-7'),163,1 (C-4), 146.0 (C-8), 143.5 (C-7), 127.3 (C-3), 97.3 (C-4'), 71.9 (C-1''), 37.4 (C-5), 34.9 (C-2''), 34.1 (C-2'''), 28.4 (C-1'''), 28.3 (C-3''), 25.5 (C-6), 22.7 (C-4''), 22.6 (C-3''), 18.2 (C-2), 14.1 (C-5''), 14.0 (C-4'''); HRMS (ESI) calc for $C_{21}H_{28}O_7Na$ $[M+Na]^+$ 415.1727, found 415.1720.

Table S6: NMR assignments for dihydro-zopfiellin **13** ([a]125 MHz, [b]500 MHz in $CDCl_3$)

**Position**	**Dihydro-zopfiellin 13**	
	**δ_C[a]**	**δ_H[b], mult. (*J* in Hz)**
**1**	41.7	2.99 – 3.06, m
**2**	18.2	2.99 – 3.06, m
**3**	127.3	2.67, d, (9.1)
**4**	163.1	-
**5**	37.4	-
**6**	25.5	2.83 – 2.92, m
**7**	143.5	3.17, app. t (12.2)
**8**	146.0	2.83 – 2.92, m
**3′**	172.7	-
**4′**	97.3	-
**7′**	165.7	-
**8′**	166.3	5.91, s
**1″**	71.9	-
**2″**	34.9	-
**3″**	28.3	3.92, t (6.3)
**4″**	22.7	1.57 – 1.69, m
**5″**	14.1	1.30 – 1.45, m
**1‴**	28.4	1.30 – 1.45, m
**2‴**	34.1	0.95, m
**3‴**	22.6	1.30 – 1.45, m
**4‴**	14.0	1.57 – 1.59, m

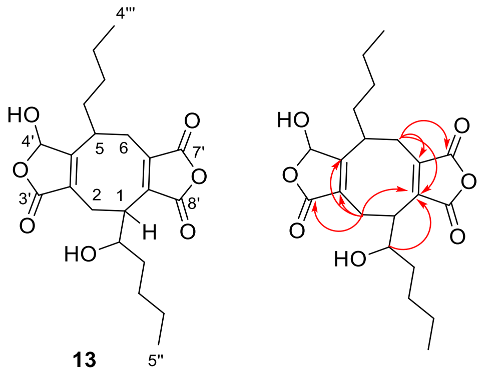

13

Figure S46: Key HMBC correlations used to aid the assignment of dihydro-zopfiellin **13**.

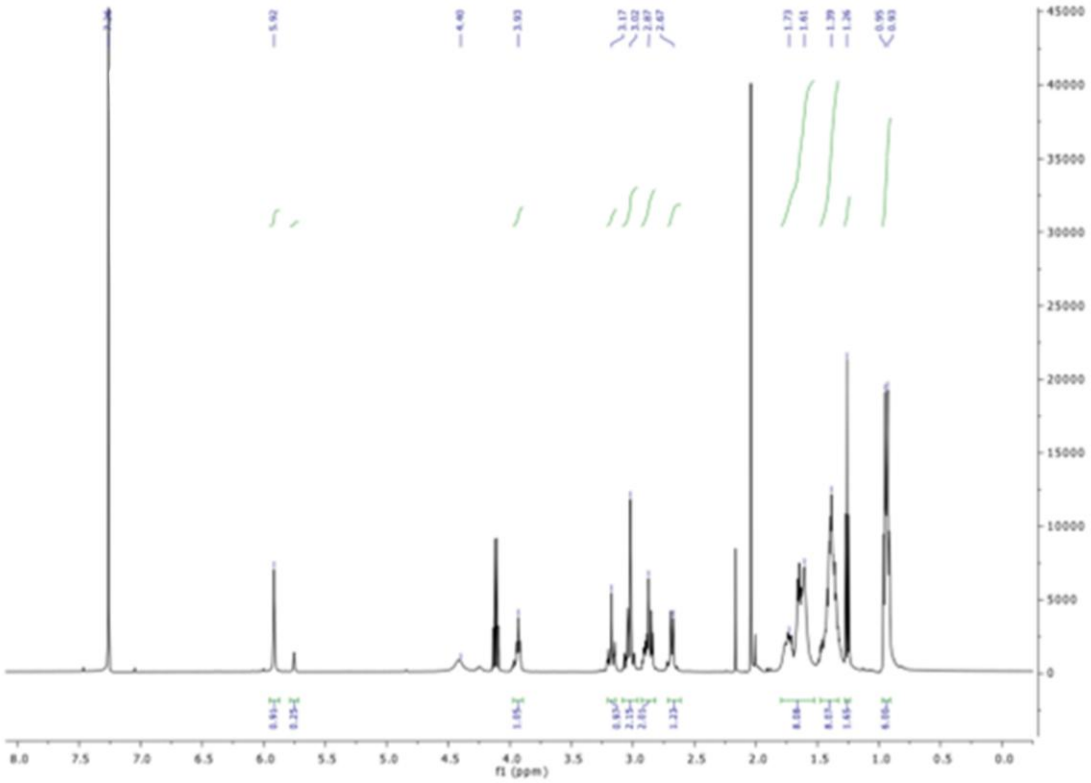

Figure S47: ^{1}H NMR (500 MHz, $CDCl_3$) spectrum of **13**.

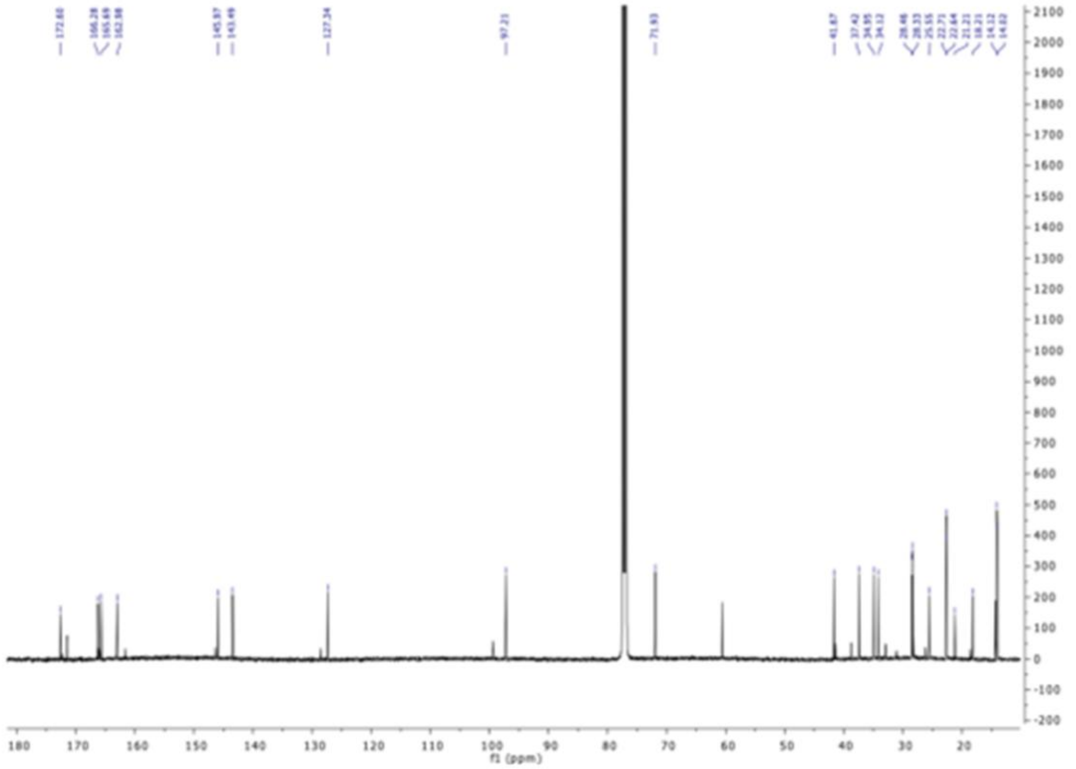

Figure S48: ^{13}C NMR (125 MHz, $CDCl_3$) spectrum of **13**.

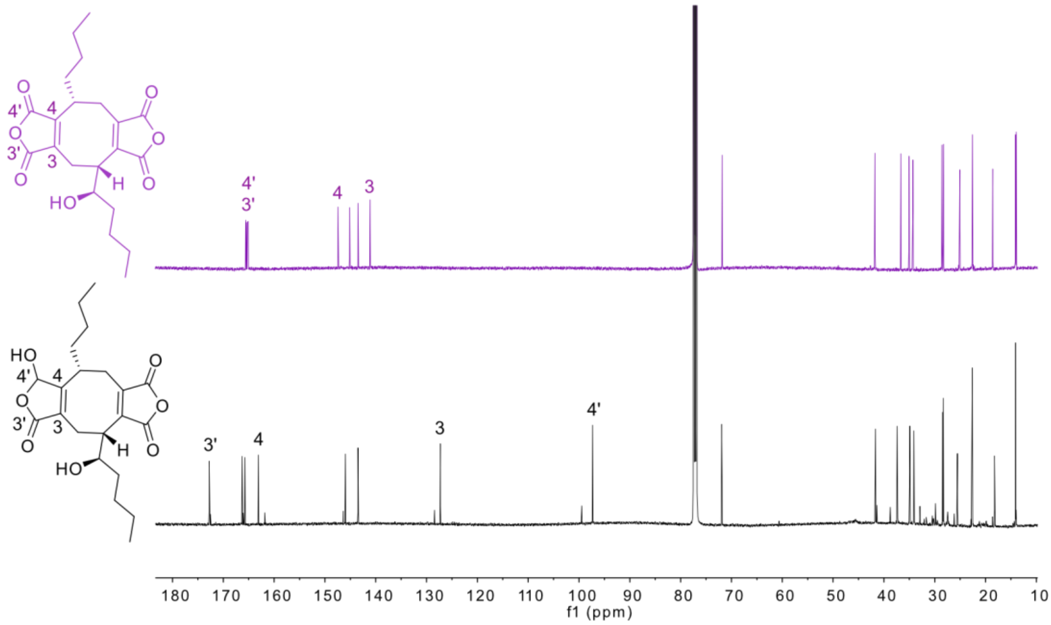

Figure S49: Comparison of the ^{13}C spectra ($CDCl_3$, 125 MHz) of zopfiellin **4** (top) and dihydro-zopfiellin **13** (bottom).

21. X-ray crystallography

X-ray diffraction experiments on castaneiolide **10** were carried out at 200(2) K on a Bruker D8 Venture using Cu-K_α (λ = 1.54178 Å) radiation. Intensities were integrated in SAINT[19] and absorption corrections based on equivalent reflections were applied using SADABS.[20] The structure was solved using ShelXT[21] and refined by full matrix least squares against F^2 in ShelXL[22] using Olex2[23]. All of the non-hydrogen atoms were refined anisotropically. While all of the hydrogen atoms were located geometrically and refined using a riding model. The molecule displayed disorder in one of the chains and the occupancies of the fragments was determined by refining them against a free variable with the sum of the two sites set to equal 1, restraints and constraints were used to maintain sensible geometries and thermal parameters. The absolute structure was not able to be determined. Crystal structure and refinement data are given in Table S7. Crystallographic data for compound **10** has been deposited with the Cambridge Crystallographic Data Centre as supplementary publication CCDC 2366244. Copies of the data can be obtained free of charge on application to CCDC, 12 Union Road, Cambridge CB2 1EZ, UK [fax(+44) 1223 336033, e-mail: deposit@ccdc.cam.ac.uk].

Table S7: Crystal data and structure refinement for castaneiolide **10**

Identification code	**10**
Empirical formula	$C_{22}H_{28}O_8$
Formula weight	420.44
Temperature/K	200(2)
Crystal system	orthorhombic
Space group	$P2_12_12_1$
a/Å	8.25833(10)
b/Å	12.69524(15)
c/Å	20.3354(3)
α/°	90
β/°	90
γ/°	90
Volume/Å^3	2131.99(4)
Z	4
ρ_{calc}g/cm^3	1.310
μ/mm^{-1}	0.831
F(000)	896.0
Crystal size/mm^3	0.342 × 0.224 × 0.187
Radiation	CuKα (λ = 1.54178)
2θ range for data collection/°	11.148 to 137.37
Index ranges	-9 ≤ h ≤ 9, -15 ≤ k ≤ 15, -24 ≤ l ≤ 23
Reflections collected	22105
Independent reflections	3875 [R_{int} = 0.0532, R_{sigma} = 0.0398]
Data/restraints/parameters	3875/28/307
Goodness-of-fit on F^2	1.114
Final R indexes [I>=2σ (I)]	R_1 = 0.0642, wR_2 = 0.2048
Final R indexes [all data]	R_1 = 0.0673, wR_2 = 0.2102
Largest diff. peak/hole / e Å^{-3}	0.34/-0.43

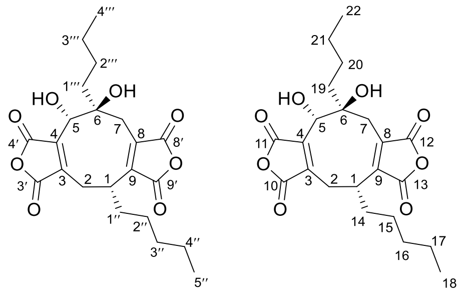

Figure S50: Comparison of labelled castaneiolide **10** with the previously proposed maleidride numbering system (left) and X-ray structure numbering system (right).

22. References

[1] O. N. Technologies, version 22.12.7 ed., **2024**.

[2] O. N. Technologies, v 0.8 ed., **2024**.

[3] P. Danecek, J. Bonfield, J. Liddle, J. Marshall, V. Ohan, M. Pollard, A. Whitwham, T. Keane, S. McCarthy, R. Davies, H. Li, *GIGASCIENCE* **2021**, *10*.

[4] A. Zimin, G. Marçais, D. Puiu, M. Roberts, S. Salzberg, J. Yorke, *BIOINFORMATICS* **2013**, *29*, 2669-2677.

[5] A. F. A. Smit, R. Hubley, P. Green, **2013-2015**.

[6] R. Hubley, A. Smit*, Vol. 2024*, 2.0.6 ed., **2024**.

[7] K. Hoff, S. Lange, A. Lomsadze, M. Borodovsky, M. Stanke, *BIOINFORMATICS* **2016**, *32*, 767-769.

[8] M. Manni, M. Berkeley, M. Seppey, F. Simao, E. Zdobnov, *MOLECULAR BIOLOGY AND EVOLUTION* **2021**, *38*, 4647-4654.

[9] M. H. Medema, E. Takano, R. Breitling, *Molecular Biology and Evolution* **2013**, *30*, 1218-1223.

[10] K. Williams, A. J. Szwalbe, N. P. Mulholland, J. L. Vincent, A. M. Bailey, C. L. Willis, T. J. Simpson, R. J. Cox, *Angewandte Chemie-International Edition* **2016**, *55*, 6783-6787.

[11] K. Williams, K. de Mattos-Shipley, C. Willis, A. Bailey, *Fungal Biology and Biotechnology* **2022**, *9*, 2.

[12] S. Altschul, T. Madden, A. Schaffer, J. Zhang, Z. Zhang, W. Miller, D. Lipman, *Nucleic Acids Res* **1997**, *25*, 3389 - 3402.

[13] S. Hunter, P. Jones, A. Mitchell, R. Apweiler, T. K. Attwood, A. Bateman, T. Bernard, D. Binns, P. Bork, S. Burge, E. de Castro, P. Coggill, M. Corbett, U. Das, L. Daugherty, L. Duquenne, R. D. Finn, M. Fraser, J. Gough, D. Haft, N. Hulo, D. Kahn, E. Kelly, I. Letunic, D. Lonsdale, R. Lopez, M. Madera, J. Maslen, C. McAnulla, J. McDowall, C. McMenamin, H. Mi, P. Mutowo-Muellenet, N. Mulder, D. Natale, C. Orengo, S. Pesseat, M. Punta, A. F. Quinn, C. Rivoire, A. Sangrador-Vegas, J. D. Selengut, C. J. A. Sigrist, M. Scheremetjew, J. Tate, M. Thimmajanarthanan, P. D. Thomas, C. H. Wu, C. Yeats, S.-Y. Yong, *Nucleic Acids Research* **2012**, *40*, D306-D312.

[14] M. L. Nielsen, L. Albertsen, G. Lettier, J. B. Nielsen, U. H. Mortensen, *Fungal Genetics and Biology* **2006**, *43*, 54-64.

[15] R. C. Edgar, *Nucleic Acids Research* **2004**, *32*, 1792-1797.

[16] K. M. J. de Mattos-Shipley, C. E. Spencer, C. Greco, D. M. Heard, D. E. O'Flynn, T. T. Dao, Z. Song, N. P. Mulholland, J. L. Vincent, T. J. Simpson, R. J. Cox, A. M. Bailey, C. L. Willis, *Chemical Science* **2020**, *11*, 11570-11578.

[17] W. C. Still, M. Kahn, A. Mitra, in *JOURNAL OF ORGANIC CHEMISTRY, Vol. 43*, **1978**, pp. 2923-2925.

[18] C. Li, R. Yang, Y. Lin, S. Zhou, *Chemistry of Natural Compounds* **2006**, *42*, 290-293.

[19] Bruker, *SAINT+ v8.38A Integration Engine, Data Reduction Software, Bruker Analytical X-ray Instruments Inc., Madison, WI, USA* **2015**.

[20] Bruker, *SADABS 2014/5, Bruker AXS area detector scaling and absorption correction, Bruker Analytical X-ray Instruments Inc., Madison, Wisconsin, USA* **2014/5**.

[21] G. M. Sheldrick, *Acta Crystallographica a-Foundation and Advances* **2015**, *71*, 3-8.

[22] G. M. Sheldrick, *Acta Crystallographica Section A* **2008**, *64*, 112-122; G. M. Sheldrick, *Acta Crystallographica Section C-Structural Chemistry* **2015**, *71*, 3-8.

[23] O. V. Dolomanov, L. J. Bourhis, R. J. Gildea, J. A. K. Howard, H. Puschmann, *Journal of Applied Crystallography* **2009**, *42*, 339-341.
